# Electrostatic Anchoring in RNA-Ligand Design—Dissecting the Effects of Positive Charges on Affinity, Selectivity, Binding Kinetics, and Thermodynamics

**DOI:** 10.1021/acs.jmedchem.5c00339

**Published:** 2025-04-07

**Authors:** Laura Almena Rodriguez, Elisabeth Kallert, Jan-Åke Husmann, Kirsten Schaubruch, Katherina I. S. Meisel, Marvin Schwickert, Sabrina N. Hoba, Ralf Heermann, Christian Kersten

**Affiliations:** † Institute of Pharmaceutical and Biomedical Sciences, Johannes Gutenberg-University, Staudingerweg 5, 55128 Mainz, Germany; ‡ Institute of Molecular Physiology, Microbiology and Biotechnology, Johannes Gutenberg-University, Hanns-DieterHüsch-Weg 17, 55128 Mainz, Germany; § Institute for Quantitative and Computational Biosciences, Johannes Gutenberg-University, BioZentrum I, Hanns-Dieter-Hüsch Weg 15, 55128 Mainz, Germany

## Abstract

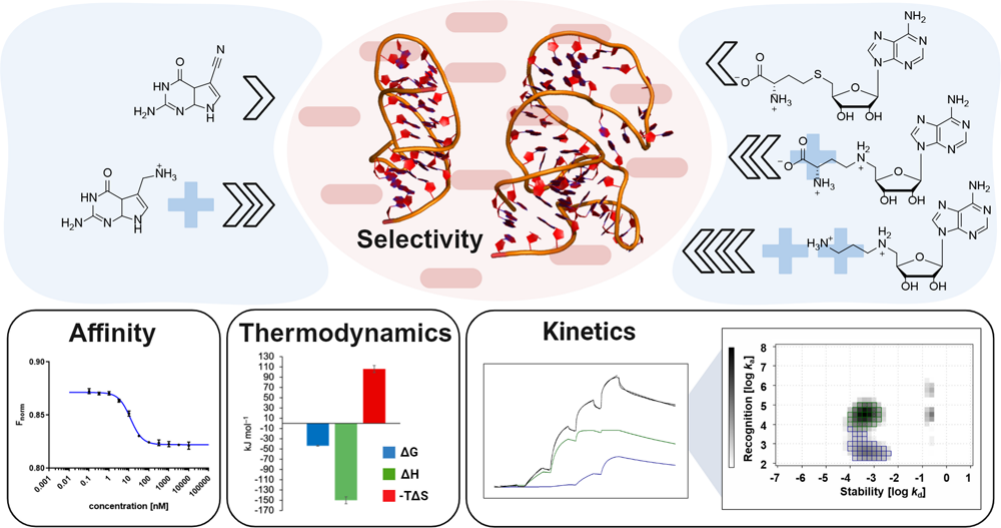

Targeting RNA with small molecules is an emerging field in medicinal chemistry. However, highly potent ligands are often challenging to achieve. One intuitive strategy to enhance ligand’s potency is the implementation of positively charged moieties to interact with the negatively charged RNA phosphate backbone. We investigated the effect of such “electrostatic anchors” on binding affinity, kinetics, thermodynamics, and selectivity by MST, SPR, and ITC experiments, respectively, with the *Ba* SAM-VI riboswitch and the *Tte* preQ_1_ riboswitch aptamer model systems. RNA-ligand interactions were dominated by enthalpy, and electrostatic anchors had moderate effects on binding affinity driven by faster association rates for higher charged ligands. Despite the observations of loose binding interactions in SPR experiments with multibasic ligands, selectivity over structurally unrelated RNA off-targets was maintained. Therefore, the addition of positively charged moieties is no universal RNA-ligand design principle, but a purposefully implemented ionic RNA-ligand interaction can enhance potency without impairing selectivity.

## Introduction

In recent years, interest in the use of ribonucleic acids (RNAs) as a therapeutic agent, as well as their potential as a drug target has been growing. This has been reflected in the development of new vaccines, antisense oligonucleotides (ASOs), and small interfering RNAs (siRNAs). Further, targeting bacterial riboswitches, viral RNA, or human dysregulated RNA elements holds the potential for novel antibiotics, antivirals, and treatments for previously untreatable diseases.^1−3^ However, oligonucleotide-based therapeutic agents encounter several challenges, including delivery issues and the occurrence of adverse effects.^4,5^ In contrast, small molecules offer superior pharmacokinetic properties and the potential for chemical optimization campaigns.^1,5^ Moreover, the development of RNA-binding small molecules would significantly expand the number of potential drug targets. At present, approximately 3,000 disease-related human proteins are known, many of them considered undruggable. However, targeting those proteins at the messenger RNA (mRNA) level with small molecules represents a promising approach.^6,7^ In 2020, risdiplam, the first small molecule to target RNA other than the bacterial ribosome, received FDA approval. Risdiplam binds the survival motor neuron 2 (SMN2) pre-mRNA as a splicing modulator for the therapy of spinal muscular atrophy (SMA) and was identified in a phenotypic screening.^8−10^ While several examples of RNA-binding small molecules were reported, optimization often proofed to be difficult.^7^ One general challenge in RNA hit optimization and affinity enhancement is the requirement of a high information content at the target site arising from unpaired bases and a subsequent complex, druggable tertiary structure.^7,11^ Based on this theory, high information content RNAs tend to be more likely to accommodate more potent ligands. Likewise, these regions are rare which also should result in selectivity, particularly in the cellular context where numerous low information content RNA motifs are present, but a structurally similar high information content RNA off-target is unlikely. By acquiring knowledge about the structural properties and potential unique binding sites of a target RNA, ligands can be rationally optimized.^7,11^ However, there is currently no guideline on how to design high-affinity, selective, drug-like^12,13^ RNA-targeting small molecules, especially for lower information content targets. Affinity optimization was addressed through a variety of different strategies in these cases, but no universal approach has been identified.^1−3^

**Figure 1:**
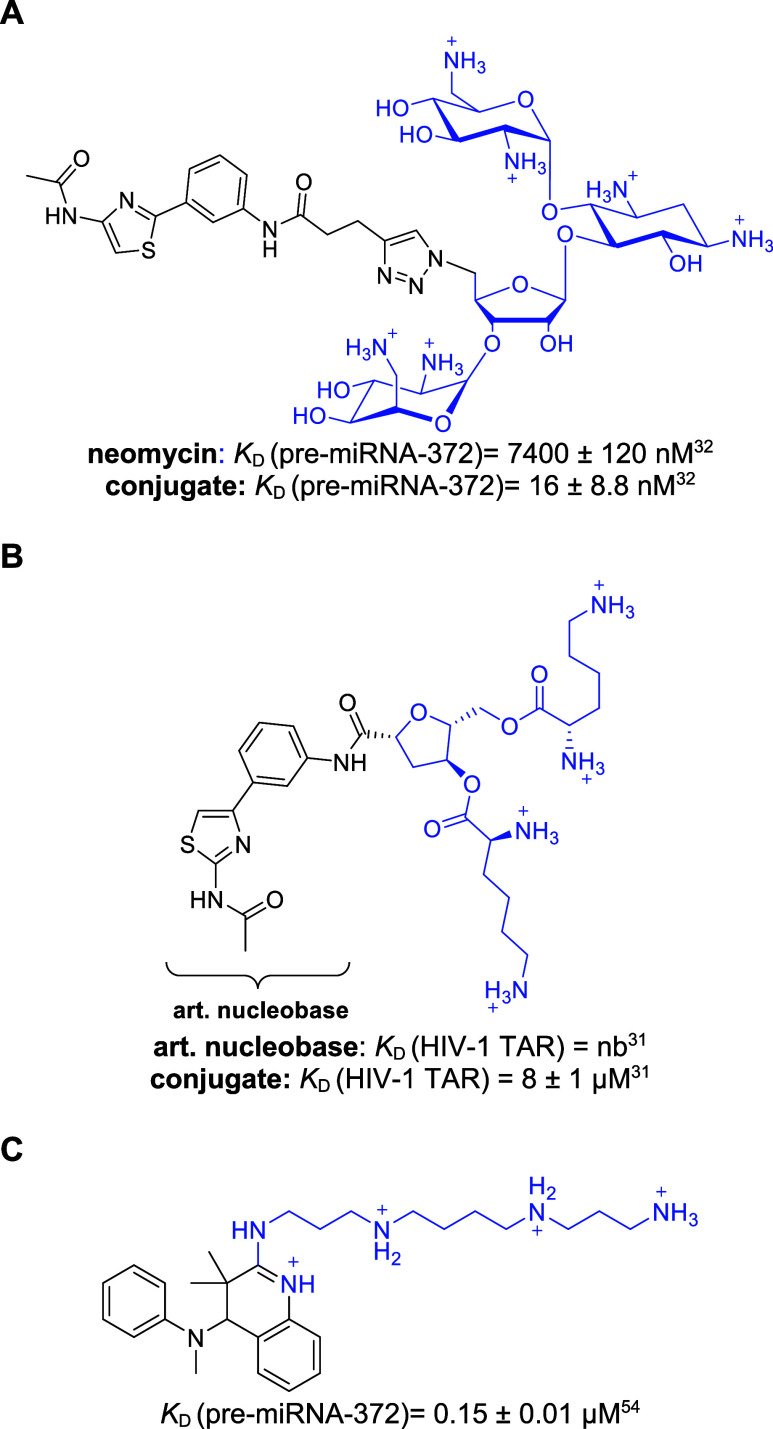
Molecular structures and binding constants *K*_D_ of (A) neomycin-nucleobase conjugate binding to pre-miRNA-372,^32^ (B) artificial (art.) nucleobase - basic amino acid conjugate binding to HIV-1 TAR RNA,^31^ and (C) a polyamine binding to pre-miRNA-372.^54^ Polycationic moieties are highlighted in blue.

Different strategies are under elucidation to overcome limited affinity. Ribonucleic targeting chimeras (RiboTACs)^5,14−19^ and proximity-induced nucleic acid degraders (PINADs)^20^ aim for the degradation of the target RNA requiring only substoichiometric amounts of ligands like it is established for proteins by proteolysis targeting chimeras (PROTACs).^21−23^ An intuitive concept to increase RNA-ligand affinity, is the conjugation of RNA-binding motifs with positively charged groups to interact with the negatively charged RNA backbone. Plain attractive forces of ionic interactions are masked by a diffuse ion atmosphere around nucleic acids.^24−26^ Still, statistical evaluation of RNA-binding small molecules indicated beneficial effects arising from positively charged groups.^27,28^ This concept was further explored by Maria Duca and co-workers using aminoglycoside-conjugates.^29−32^ Aminoglycosides are known antibiotics targeting the bacterial ribosome. Polar interactions between their amino and hydroxyl groups with the RNA backbone or nucleobases can likely occur^29^ and play a leading role in short-range interactions,^33−35^ but also in the diffusion toward the ribosomal A-site.^36,37^ A correlation between higher charge and improved affinity or activity to its target was shown using computational^34,38^ and experimental^35,39^ methods. The polycationic character of aminoglycosides enhances binding but reduces their specificity causing severe side-effects like nephrotoxicity or ototoxicity.^40−42^ This is also reflected in the reoccurrence of aminoglycosides as ligands for multiple unrelated RNA targets.^30,32,43−47^ In the attempts to target other RNAs and to exploit their beneficial electrostatic interactions, aminoglycosides have been conjugated with various specific RNA-ligands to increase their selectivity and affinity.^29−32,44,48−51^ Exemplarily, neomycin was linked to an aminophenyl-thiazole-based artificial nucleobase resulting in an improved binder of an oncogenic pre-microRNA (miRNA, Figure 1A).^32^ This artificial nucleobase was designed to specifically interact with T-A pairs.^52,53^ Aminoglycoside conjugates were also found to target viral^31,44,48^ and oncogenic^30,32,49−51^ RNAs and to be sufficiently selective in cellular assays.^30,32,49,50^ The implementation of basic amino acids showed similar beneficial effects.^30,31^ For example, basic amino acids were linked to an artificial nucleobase to bind to the human immunodeficiency virus trans-activation response elements (HIV TAR, Figure 1B).^31^ Likewise, functionalized polyamines were identified to inhibit biogenesis of oncogenic pre-miRNAs in a screening. The highly active polyamine (Figure 1C) affected only a small set of oncogenic miRNAs and showed specific inhibition of cancer cell proliferation.^54^ Implementing cationic moieties, like spermidines,^54,55^ within RNA-targeting ligands can thus be used to increase affinity while not necessarily resulting in unselective, promiscuous RNA-binders.^3^

RNA-small molecule interactions are commonly described by binding affinities or biological activity. However, the additional determination of kinetic and thermodynamic parameters provides insights into fundamental principles of molecular recognition between RNA and small molecules.^56^ Binding of some polycationic molecules, like the aminoglycoside neomycin^57^ or polyamide amino acids,^58^ to their target RNA were found to be enthalpy-driven. It was suggested that their polycationic moieties are involved in specific noncovalent interactions.^56,58^ Polyamines were found to modulate RNA functions via specific interactions and to play a structural role distinct from divalent cations.^59^ Comparing the thermodynamic binding profiles of similar compounds can give insight into the energetic contributions of small structural ligand changes, which can guide rational design.^60^ The binding profiles of RNA ligands for on- and off-targets can help to explain selectivity patterns.^61^ In addition, for bacterial riboswitches that are under kinetic control, a fast-associating ligand will likely be more efficient, since it has to compete with the natural ligand.^62^

**Figure 2:**
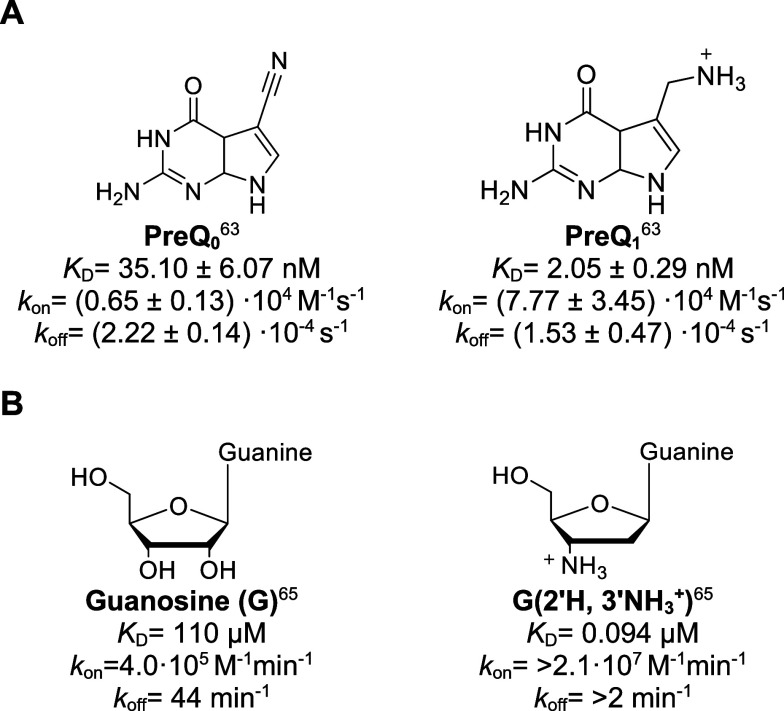
Molecular structures, binding constants *K*_D_ and kinetic parameters *k*_on_/*k*_off_ of (A) preQ_1_ compared to preQ_0_ binding to the *Tte* preQ_1_ riboswitch^63^ and (B) guanosine and its amine derivative binding to *tetrahymena* group I ribozyme.^65^

The influence of cationic moieties within nonaminoglycoside RNA-binding small molecules on their kinetic profiles was evaluated in a few studies.^63−65^ Wedekind and co-workers compared the natural ligands prequeuosine-1 (preQ_1_) and preQ_0_ (Figure 2A) binding to the *Thermoanaerobacter tengcongensis* (*Tte*) preQ_1_ riboswitch aptamer. PreQ_1_ bears a methylamine group, which is replaced by a nitrile group in the case of preQ_0_. Thus, preQ_1_ is positively charged under physiological pH in contrast to preQ_0_ which is neutral. The association rate of preQ_1_ was found to be 12-fold faster than preQ_0_ while dissociation rates were similar. This results in the higher affinity observed for preQ_1_.^63^ Micura and co-workers synthesized amino- and azido-functionalized derivatives of preQ_1_ and tested them in in vitro and in vivo assays. Amino-functionalized derivatives showed enhanced *k*_on_-values and affinities in agreement with the expectation of adding electrostatic interactions.^64^

For the *tetrahymena* group I ribozyme, one hydroxy group of the cofactor guanosine was exchanged with an amino moiety (Figure 2B). It was hypothesized that the introduced basic group replaces a magnesium ion from the binding site. This led to a faster association rate and longer residence times of an early binding complex, which were necessary to enhance productive binding. Herschlag and co-workers termed this cationic group a “molecular anchor.” The strategy was proposed to be a universal principle to enhance association rates for well-placed anchors.^65^ In a more poetical way, this effect of electrostatic anchoring can be regarded as a type of “Circe effect.”^66^ This effect describes attractive long-range interactions to conduct a substrate into the active site of an enzyme, where it will be converted. It was defined by William P. Jencks and is named after Circe, a goddess in Greek mythology, luring men and turning them into pigs. In case of RNA-binding small molecules this effect applies to the association toward the binding site without the following conversion.

Consequently, introduction of positive charges into RNA-ligands might be a promising design strategy to improve affinity by enhanced association rates. However, conjugation of RNA-ligands to polycationic moieties like aminoglycosides may not hold up as a universal RNA-ligand design concept due to toxicity, selectivity or pharmacokinetic issues. A deeper understanding of how positively charged moieties impact RNA-ligand binding affinity, selectivity, kinetics and thermodynamics is required to improve current design and development concepts for RNA-targeting small molecules. Therefore, we used the preQ_1_ riboswitch and SAM-VI riboswitch aptamers as model systems to investigate the impact of ligand charges on affinity, binding kinetics, thermodynamics and selectivity. Surface plasmon resonance (SPR) spectroscopy, isothermal titration calorimetry (ITC) and microscale thermophoresis (MST) were used to examine the effects of electrostatic interactions on binding behavior. We aim to answer the questions: (1) Are positively charged groups generally leading to more potent RNA-ligands, (2) do these additional charges impair selectivity, (3) (how much) do positive charges enhance association rates, (4) what are the resulting thermodynamic binding profiles and (5) can general design principles around charged moieties be deduced for the development of RNA-binding small molecules?

## Results and Discussion

### Rational Design of Charged Ligands

**Figure 3:**
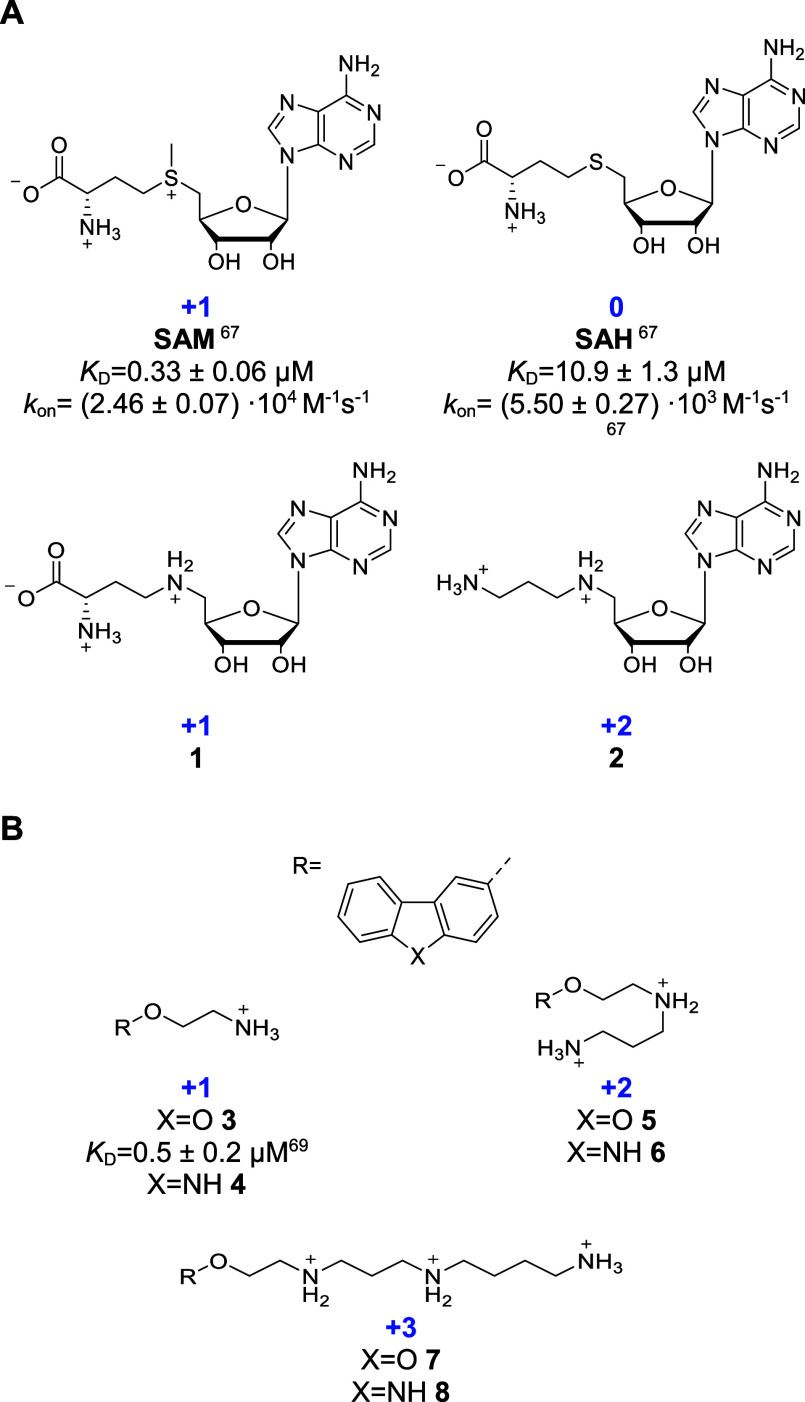
Polycationic small molecules under elucidation. (A) Ligands of the *Ba* SAM-VI riboswitch. (B) Reported and putative synthetic ligands of the *Tte* preQ_1_ riboswitch. The number of formal charges is shown in blue.

**Figure 4:**
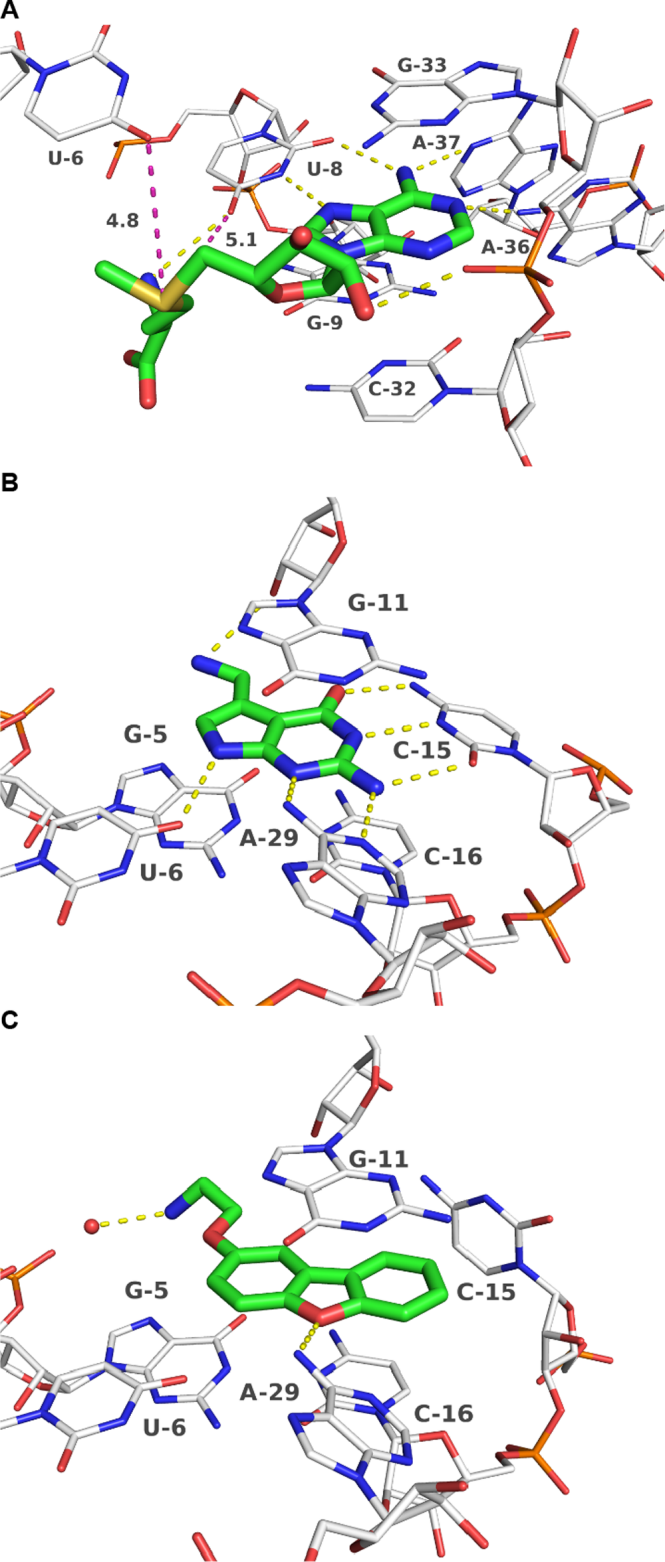
(A) *Ba* SAM-VI riboswitch in complex with SAM (PDB-ID: 6LAS). Distances between sulfonium ion and oxygen atoms (O4) of U6 and U8, respectively, are depicted as magenta dashed lines with distances in Å.^67^ (B) *Tte* preQ_1_ riboswitch in complex with preQ_1_ (PDB-ID: 3Q50).^63^ (C) preQ_1_ riboswitch in complex with synthetic ligand **3** (PDB-ID: 6E1S).^69^ Ligands are depicted with green, RNA with white carbon atoms. Polar interactions are shown as yellow dashed lines.

Several RNA motifs are capable to discriminate between charged and uncharged natural ligands, including preQ_1_ and SAM riboswitches, which strongly discriminate between preQ_1_ and preQ_0_ (Figure 2A),^63^ and *S*-adenosyl methionine (SAM) and *S*-adenosyl homocysteine (SAH), respectively (Figures 3 and 4A).^67,68^ Therefore, these RNAs can serve as ideal model systems for the investigation of charge effects on binding behavior. Particularly, the enhancement of association rates as a direct consequence of increased positive ligand charges was investigated. In this study, we selected two well-studied riboswitches for analysis: the *Tte* preQ_1_ and the *Bifidobacterium angulatum* (*Ba*) SAM-VI riboswitch aptamer domains. We modified reported ligands of the respective riboswitches (Figure 3) to elucidate the impact of charges on binding affinity, selectivity, kinetics and thermodynamics. Basic and under physiological conditions positively charged groups were added at ligand moieties oriented out of the binding site to minimize effects via direct ionic interactions with binding site residues and to better describe long-range attraction and solvent-exposed interactions.

The SAM-VI riboswitch belongs to the widespread family of SAM-sensing riboswitches, which are involved in the regulation of SAM metabolism.^67,70^ SAM is a ubiquitous methylation cofactor, and these riboswitches are found in the majority of known bacterial species.^71,72^ SAM riboswitches strongly discriminate between the positively charged SAM and its neutral methylation side product SAH. For example, the *Ba* SAM-VI riboswitch has a 33-fold higher affinity to SAM compared to SAH.^67,68^ Previously, it was demonstrated that the positively charged sulfonium moiety of SAM is the primary factor responsible for its selective binding to the associated riboswitches.^67,73−75^ The SAM-binding site of the SAM-VI riboswitch lies at the junction between stems P1 and P2. The binding pocket consists of the canonical Watson–Crick base pair G9–C32 and the noncanonical *trans* Watson–Crick base pair G7–G33. Additionally, the four continuously stacked bases A34, A36, A37, and G38 and the junctional U8 form a unique binding pocket. SAM adopts an extend conformation with the methionine tail exceeding the binding site. The adenine moiety forms hydrogen bonds with the Watson–Crick edge of U8, A36 and A37 as well as stacking interactions with G9–C32 and G33.^67^ Notably, the positively charged sulfonium moiety seems to not directly interact with binding site residues (Figure 4A).^67^ The closest potential interacting atoms are oxygens (O4) of U6 and U8 which might occasionally interact by charge-assisted chalcogen bonds as described for SAM-I riboswitches.^76^ To investigate the influence of charges on binding behavior in detail, the overall neutral, zwitterionic SAH, SAM and its aza-analogue compound **1** carrying one positive net charge, and **2** which carries two positive net charges due to removal of the carboxylic acid were tested (Figure 3A). Even though the SAM-VI riboswitch strongly discriminates between SAM and SAH, none of the modified groups directly interacts with the riboswitch’s buried binding site (Figure 4A).

**Scheme 1:**
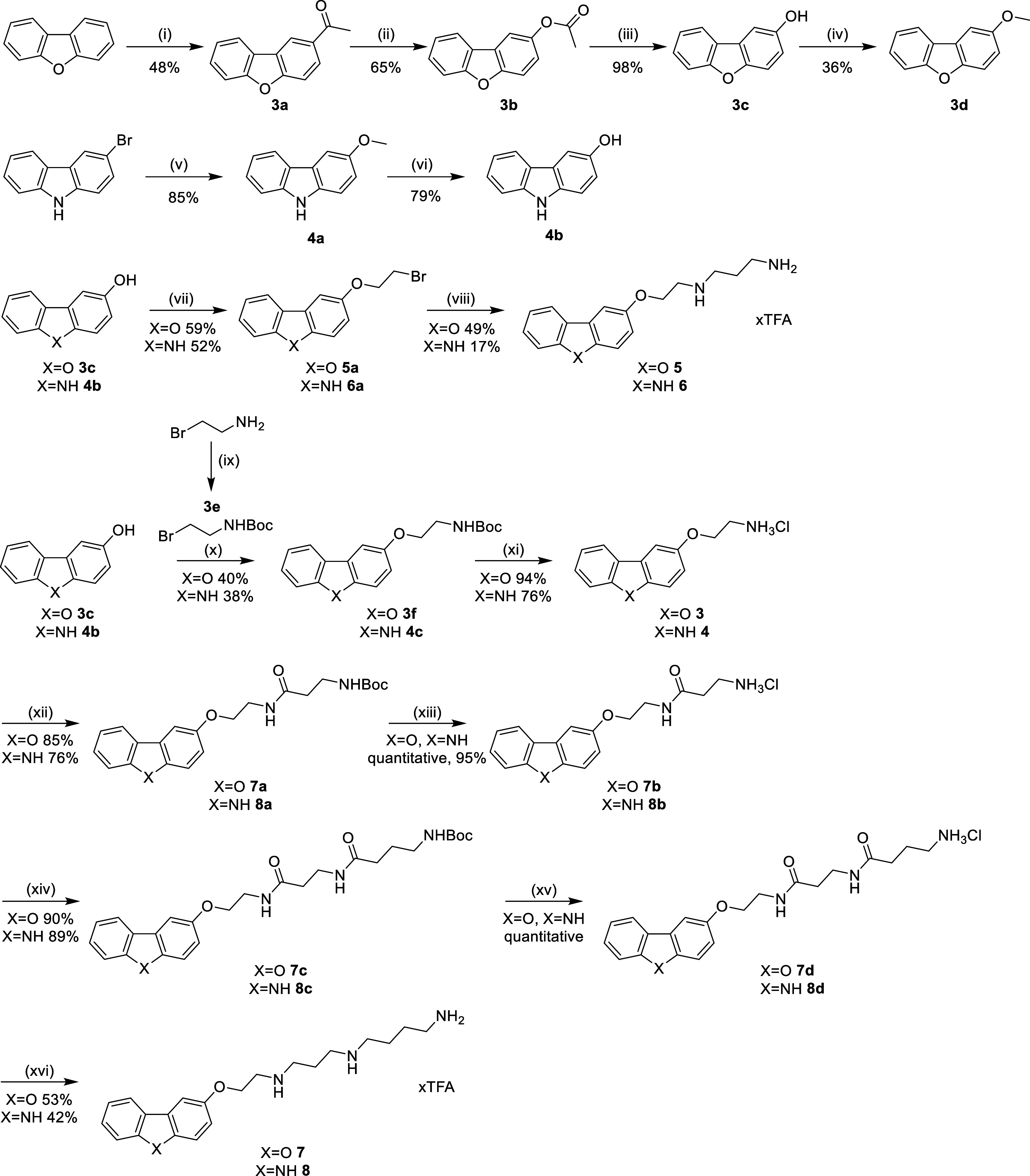
Synthesis of Compounds **3**–**8** Reagents and conditions: (i) acetyl chloride, AlCl_3_, chloroform, rt, 4 h; (ii) 2,2,2-trifluoroacetic acid, 3-chloroperoxybenzoic acid, DCM, 0 °C → rt, 3 d; (iii) sodium methanolate, methanol, 0 °C → rt, 1 h; (iv) NaH, THF, 0 °C, 10 min, then iodomethane, rt, 1 h; (v) sodium methanolate, CuI, DMF, rt → 120 °C; (vi) 47% HBr, acetic acid glacial, reflux, 6 h; (vii) 1,2-dibromoethane, K_2_CO_3_, acetonitrile, 80 °C, 22–24 h; (viii) propane-1,3-diamine, acetonitrile, 80 °C, 3 h; (ix) Boc_2_O, triethylamine, DCM, 0 °C → rt, 2 h; (x) K_2_CO_3_, acetonitrile, 80 °C, 18–20 h; (xi) 4 M HCl in 1,4-dioxane, rt, 3–20 h; (xii) 3-(*tert*-butoxycarbonylamino)propionic acid, TBTU, DIPEA, DCM, 0 °C → rt, 23 h; (xiii) 4 M HCl in 1,4-dioxane, rt, 1.5–20 h; (xiv) 4-(*tert*-butoxycarbonylamino)butyric acid, TBTU, DIPEA, DCM, 0 °C → rt, 23 h; (xv) 4 M HCl in 1,4-dioxane, rt, 1–20 h; (xvi) 1 M THF-BH_3_, THF, 0 °C → 60 °C, 3 d.

The second model system is the *Tte* preQ_1_ riboswitch aptamer. Additional to its discrimination of the natural ligands preQ_1_ (one positive net charge) and preQ_0_ (neutral),^63^ Connelly et al. reported the selective binding of dibenzofuran- and carbazole-based ligands with submicromolar potency (compound **3**, Figure 3B).^69^ PreQ_1_ and preQ_0_ were used for assay establishment. The high affinity and association-rate of preQ_1_ (*K*_D_ = 2.1 nM, *k*_on_ = 7.8 × 10^4^ 1/Ms)^63^ limits further elucidation of additional charges as higher affinities and association rates are at the resolution limit of the assays used.^77,78^ Therefore, polycationic derivatives of the lower-affinity, synthetic ligands were obtained and tested (**3**–**8**, Figure 3B, Scheme 1). The *Tte* preQ_1_ riboswitch binding pocket consists of the base quartets A28–C16–G5–A27 forming the “floor” and A14–G11–C30–C7 forming the “ceiling.” PreQ_1_ stacks in between these residues and interacts via additional hydrogen bonds to U6, C15 and A29 (Figure 4B). PreQ_0_ differs from preQ_1_ in having a nitrile moiety instead of an amino group. This amino group contributes to the stability of the RNA-preQ_1_ complex by hydrogen bonds with G11. The loss of this charged group results in a reduced affinity and association rate.^63,69^ Dibenzofuran ligand **3** is positioned similar to preQ_1_ in the binding pocket (Figure 4C). The dibenzofuran ring is stacked between the G11 “ceiling” and G5–C16 “floor.” The furan oxygen atom forms a hydrogen bond to A29.^69^ Different from the native ligand’s binding mode, C15 is rotated to form a face-to-edge π-stacking interaction with compound **3** instead of the Watson–Crick-like pair found in the preQ_1_-complex. Like for the SAM-VI riboswitch, the side chain of ligand **3** is oriented out of the binding site (Figure 4C) making it an ideal attachment point for additional basic centers (ligands **5**–**8**, Figure 3B).

### SAM-VI Riboswitch Binding Studies

#### Binding Affinity

SPR, MST, and ITC experiments of SAM, SAH, compounds **1** and **2** with the *Ba* SAM-VI riboswitch aptamer domain were performed (Table 1). Results for the reference ligands SAM and SAH are in good agreement with literature reported values (Figure 3A) and variations between SPR, MST, and ITC are within the usual range when comparing different assay methods.^79−81^ In contrast to the zwitterionic, net neutral SAH, SAM carries an additional methyl group at the sulfur atom forming a sulfonium ion with a formal charge of +1. Strong discrimination between SAM and SAH was observed across the different assays. Discrimination factors for the binding affinities showed that SAH binds between 21-fold and 48-fold weaker than SAM. These results are in good agreement with previously reported ITC data showing a discrimination factor of 33^67^ in *K*_D_-values. Lim *et al.* reported previously, that the positively charged sulfonium moiety is the key factor of SAM selectivity in SAM-I riboswitches as binding modes of SAM and SAH are highly similar.^73^ Likewise, similar ligand conformations for SAM and SAH are also found for the SAM-VI riboswitch (Figures 3A and S25). The aza-derivative of SAH, **1**, carries one positive formal charge like SAM, but lacks the sulfonium methyl group. The binding affinity of compound **1** with a *K*_D_-value of 33.3 μM was comparable to SAH (34.4 μM) in MST. For ITC (3.31 μM) and in SPR a higher affinity (12.3 μM) than SAH (*K*_D_ = 14.0 μM in ITC and 150 μM in SPR, respectively) was observed. The exchange of sulfur for nitrogen and with it introducing an additional positive charge compared to SAH, did still result in lower binding affinities compared to SAM (*K*_D_-values between 0.7 μM in MST and ITC, and 3.7 μM in SPR). A charge-assisted chalcogen bond is discussed as the driving factor of the tight binding of SAM to various SAM riboswitches. The Coulombic attraction between the positive charge of the sulfonium ion and an electron donor is enhanced by the interaction between the sulfur σ-hole and the electron donor. In the case of the *Ba* SAM-VI riboswitch, oxygen atoms (O4) of U6 or U8 could potentially act as the electron donor atoms (Figure 4A).^76^ This might explain the higher affinity of SAM compared to its aza-analogue **1** despite the same formal charge of +1. However, different size between sulfur and nitrogen and the presence of a methyl group may also contribute to affinity. Further the sulfonium ion is permanently charged while ammonium could be partially deprotonated (p*K*_a_-values of 7.6 and 7.8 for **1** and **2**, respectively, calculated using MOE2022.02^82−84^). Compound **2** lacks the carboxylate functionality of the other SAM-derivatives resulting in two positive formal charges. The binding affinities determined with all three methods (SPR, MST, ITC) were comparable with values in the one-digit micromolar range. Compound **2** showed enhanced affinity compared to the net uncharged SAH and **1** (formal charge +1). Thus, the series SAH, compound **1** and **2** revealed a trend of increased affinity by higher formal charges, and with SAM being superior to nonsulfonium-containing molecules.

**Table 1:**
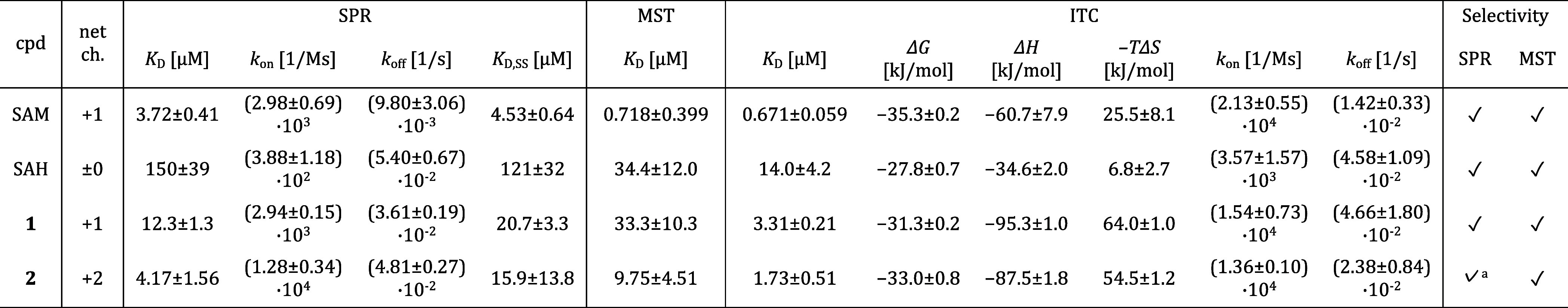
SPR, MST, ITC, and Selectivity over *Tte* preQ_1_ Riboswitch Results for SAM**-**VI Riboswitch Compounds (cpd)^b^

a SPR-sensorgrams show fast-on and fast off-rates indicating a loose binding (see Figure 5B,D).

b Net charge (net ch.), dissociation constant (*K*_D_), association rate constant (*k*_on_), dissociation rate constant (*k*_off_), dissociation constant derived from SPR steady-state analysis (*K*_D,SS_). Gibbs binding free energy (Δ*G*), enthalpy (Δ*H*) and temperature-dependent entropy change (*−T*Δ*S*). Kinetic parameters for ITC experiments were determined using kinITC.^95−97^

#### Binding Kinetics

Comparison of the kinetic parameters determined by SPR and kinITC (Table 1, Tables S1 and S4, Figures S1 and S17) showed similar trends in binding kinetics. Especially in SPR, the gold standard method for the determination of binding kinetics, the sum of formal charges correlated with faster association rates. SAH showed a *k*_on_-value of 3.88 × 10^2^ 1/Ms, which was increased up to 1.28 × 10^4^ 1/Ms for compound **2**. Additional positively charged moieties seem to be responsible for this effect. Notably, increasing formal charges from +1 in compound **1** to +2 in compound **2** had minor impact (*k*_on_-factor 4.4 in SPR) than from ± 0 (SAH) to +1 (compound **1**, *k*_on_-factor of 7.6). In kinITC, the association rates showed overall similar trends, with SAH being the slowest one binding, even though absolute *k*_on_-values differ from SPR (*k*_on_ = 3.88 × 10^2^ 1/Ms in SPR, 3.57 × 10^3^ 1/Ms in kinITC). Differences in absolute values might originate from fundamental differences between both methods like immobilization or in-solution measurements, differences in used concentrations or assumptions made for kinetic characterizations. Still, the similar trends observed allow for the interpretation of relative values within the ligand series. In kinITC, the effect of additional charges was also less pronounced for the exchange from +1 to +2 formal charge resulting in overall similar *k*_on_-values for SAM, **1** and **2**, while for the net neutral SAH slower association was observed. The dissociation rates were broadly similar across both methods and all compounds showed *k*_off_-values between 0.98 × 10^–2^ and 5.40 × 10^–2^ 1/s. Thus, different binding affinities can be primarily attributed to differences in increased association rates as observed in SPR experiments.

#### Binding Thermodynamics

Binding stoichiometry for the SAM-VI riboswitch aptamer was below 1 (*n* = 0.3–0.7, Table S3) which is an indicator for target heterogeneity, probably due to partially improper folding. For the binding-competent fraction of the SAM-VI riboswitch aptamer, binding of all ligands was highly enthalpy-driven with Δ*H* ranging from −34.6 kJ/mol (SAH) to −95.3 kJ/mol (**1**). Highest enthalpic contributions were observed for compounds **1** and **2**. But these compounds showed only a moderate increase of Δ*G* compared to SAH due to a higher entropic penalty (*−T*Δ*S* = +64.0 kJ/mol for **1** and +54.5 kJ/mol for **2**, respectively) indicative for a strong enthalpy–entropy compensation event.^85,86^ Similar enthalpy–entropy compensation effects were described for other polycationic compounds binding to RNA.^58,87,88^ The replacement of sulfur (SAM, SAH) by nitrogen in compounds **1** and **2** resulted in an increased enthalpic contribution with comparable binding free energies and dissociation constants to SAM. This more exothermic process indicates strong RNA-ligand interactions at the cost of entropy, suggesting a higher intrinsic stability of the formed complex.

#### Selectivity

*Pan*-RNA promiscuity and selectivity was investigated by testing SAM, SAH, **1** and **2** against the structurally unrelated *Tte* preQ_1_ riboswitch aptamer by SPR and MST (Table 1). In MST, all SAM-VI riboswitch ligands were selective with respect to their target. In SPR, binding of compound **2** to the preQ_1_ riboswitch was observed. However, differently from the on-target binding sensorgrams (Figure 5A), the off-target sensorgrams showed fast-on/fast-off characteristics which are indicative for loose, unspecific binding events (Figure 5B).^89^ In contrast, the sensorgrams of compound **2** binding to the SAM-VI riboswitch showed the typical shape for specific binding interactions with higher response units (Figure 5A). The effect of fast-on/fast-off rates was only observed for the highest charged compound **2** (Figure 5B). Thus, a correlation between unspecific binding and the sum of positive formal charges of the molecule can be assumed. In agreement with selectivity observed in MST, steady-state evaluation of the SPR data revealed saturation of compound **2** binding to the SAM-VI riboswitch (Figure 5C). In contrast, no saturation of compound **2** on the off-target could be reached (Figure 5D). This suggests accumulation of the compound on the off-target’s surface, eventually by loose, rather unspecific ionic interactions.

**Figure 5:**
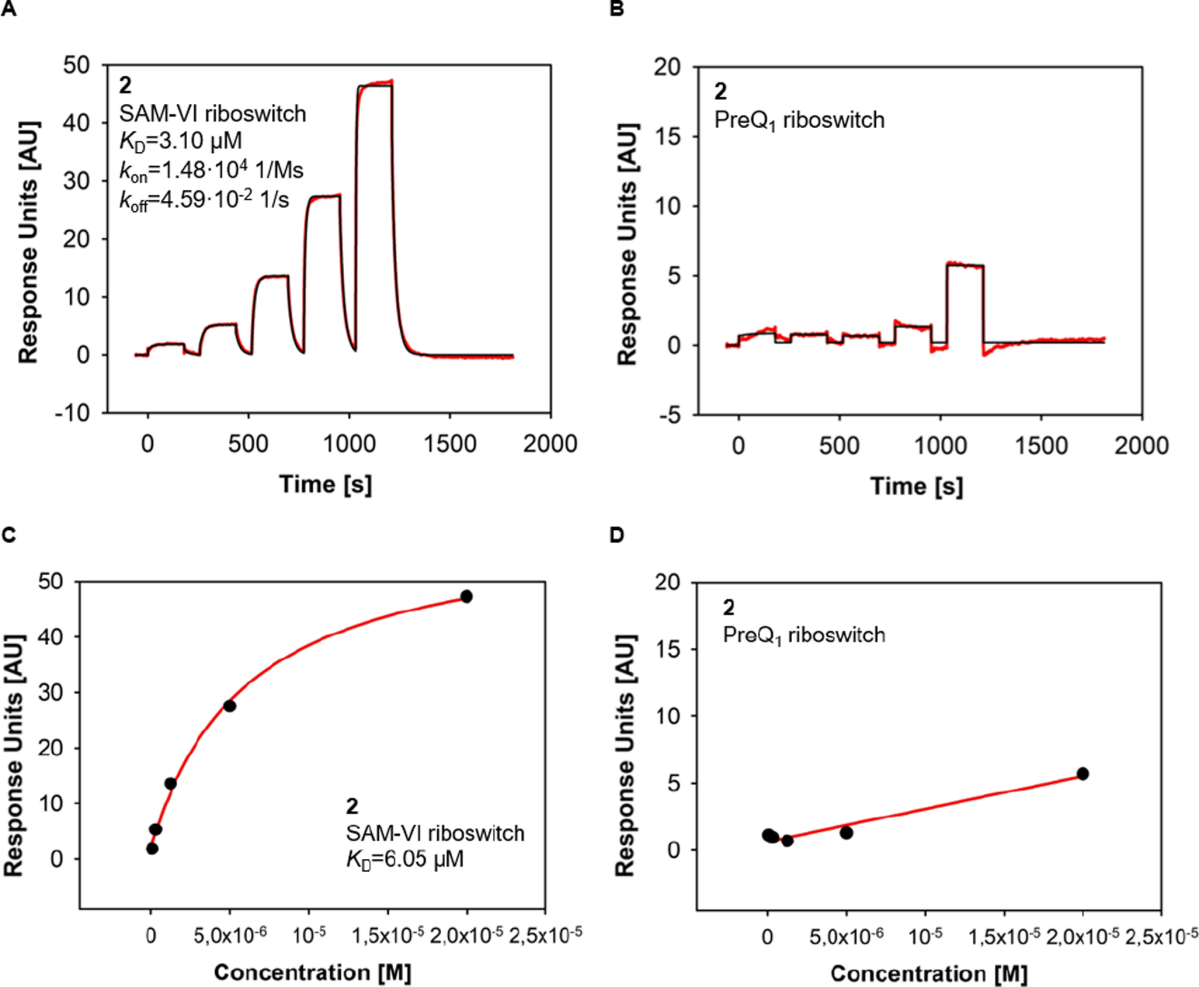
(A) Representative SPR-sensorgram of **2** binding to the SAM-VI riboswitch (*K*_D_ = 3.10 μM; average from nine measurements *K*_D_ = 4.17 μM). (B) Representative SPR-sensorgram of **2** showing loose, unspecific preQ_1_ riboswitch binding. Sensorgram is depicted in red, fit in black. (C) SPR steady-state response of **2** binding to the SAM-VI riboswitch reaching saturation (*K*_D_ = 6.05 μM; average from nine measurements *K*_D_ = 15.9 μM). (D) SPR steady-state response of **2** binding to the preQ_1_ riboswitch showing no saturation within tested concentrations (*K*_D_ = not determinable).

### PreQ_1_ Riboswitch Binding Studies

#### Binding Affinity

For the *Tte* preQ_1_ riboswitch aptamer binding of preQ_1_, preQ_0_ and synthetic ligands **3**–**8** was investigated by SPR, MST, and ITC (Table 2). We observed an enhanced affinity of the positively charged preQ_1_ over the neutral preQ_0_ with high consistency in all experiments as described in literature.^63^ PreQ_1_ showed a 3-fold higher affinity compared to preQ_0_ in SPR analysis, with being 6-fold in MST and 4-fold in ITC. The synthetic dibenzofuran ligand **3** with one positive charge was reported to bind the *Tte* preQ_1_ riboswitch with *K*_D_ = 0.5 ± 0.2 μM.^69^ Several dibenzofuran and carbazole derivatives with one positive charge showed similar affinities.^69,90,91^ We selected dibenzofuran **3** and the carbazole analog **4**, both carrying one positive charge, as starting points for further polycationic derivatives **5**–**8** (Figure 3B). In SPR, sensorgrams showed fast-on-fast-off shapes (Figure S2C–H), while SPR steady-state *K*_D_-evaluation showed no saturation in dose–response curves. For MST, poor signal-to-noise-ratios were observed prohibiting accurate determination of *K*_D_-values (Figures S4C–H and S8C–H). Likewise, ITC showed no heat signals beyond ligand dilution during titrations (Figure S12G–L). The reason why no binding was detected for **3**–**8** in these methods (MST, ITC and steady-state SPR signals) might lie in the unstable and loose binding behavior of the compounds like it was observed for analyzing the binding kinetics of **2** binding to the preQ_1_ riboswitch off-target (Figure 5B,D). Potentially, the positive charge and intercalation properties of the three-membered ring systems are responsible for unspecific interactions. These results do not necessarily contradict the results published previously.^69,90^ Possibly, the unspecific contributions overlap the specific ones in a manner that only the unspecific binding can be observed in SPR where a high negative charge density on the sensor surface is facing a (poly)cationic ligand. The synthesized multibasic dibenzofuran and carbazole derivatives **5** and **6** with two positive charges and **7** and **8** with three positive charges were designed following the findings in spermidine-like RNA binders.^54,55^ No binding was detected for these polycationic compounds in MST and ITC. The SPR sensorgrams and SPR steady-state evaluation were similar to the one-basic compounds **3** and **4**, showing unspecific loose binding without reaching saturation (Figure S2E–H). Especially for the three-basic centers carrying compound **8**, a very high response was observed (Figure S2H), indicating binding of more than one molecule. This suggested accumulation of the compound on the target’s surface by electrostatic interactions and/or intercalation.

**Table 2:**
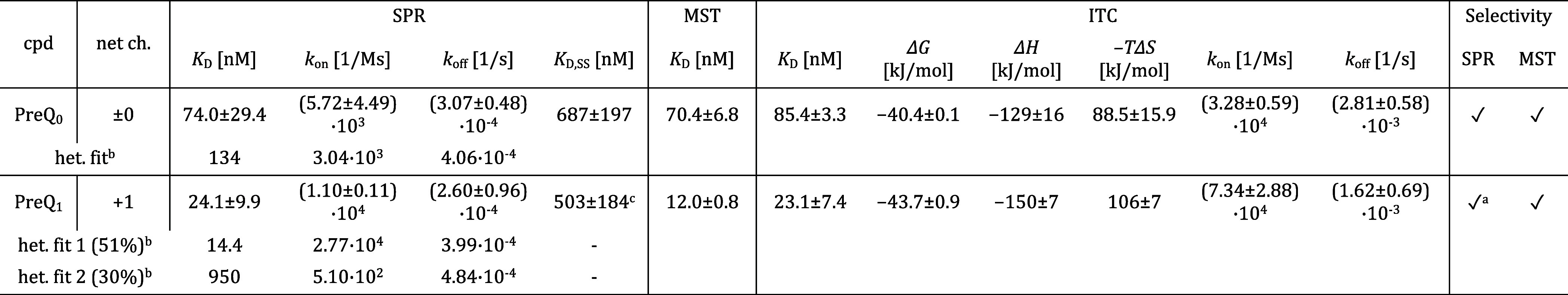
SPR, MST, ITC, and Selectivity over SAM-VI Riboswitch Results for *Tte* preQ_1_ Riboswitch Compounds (cpd)^d^

a SPR-sensorgrams show partially fast-on and fast off-rates indicating a loose binding (Figure S2B).

b IM calculation based on heterogeny target (*Tte* preQ_1_ riboswitch) fit for a representative SPR sensorgram of preQ_0_/preQ_1_. Percentage of binding mode occurrence is given in parentheses.

c Steady-state analysis is based on the 1:1 binding model which is not accurate for preQ_1_ binding to the preQ_1_ riboswitch aptamer.

d Net charge (net ch.), dissociation constant (*K*_D_), association rate constant (*k*_on_), dissociation rate constant (*k*_off_). Dissociation constant derived from SPR steady-state analysis (*K*_D,SS_). Gibbs binding free energy (Δ*G*), enthalpy (Δ*H*) and temperature-dependent entropy change (*−T*Δ*S*). Kinetic parameters for ITC experiments were determined using kinITC.^95−97^

#### Binding Kinetics

Kinetic binding parameters were determined by SPR and kinITC. Association rate constants of the positively charged preQ_1_ were 2-times higher in SPR and in kinITC compared to the uncharged preQ_0_. As described previously, *k*_off_-values for preQ_1_ and preQ_0_ were similar and the higher potency of preQ_1_ can be annotated to faster association. Again, absolute values differ between SPR and kinITC which might be due to fundamental differences in the assays including immobilization for SPR or high concentrations in ITC experiments. Still, relative values between preQ_1_ and preQ_0_ agree with literature^63^ and between the methods. The enhanced on-rate while maintaining similar off-rates resulted in the observed higher binding affinity of preQ_1_ over preQ_0_ which was attributed to the different ligand charge.

#### IM-Analysis

**Figure 6:**
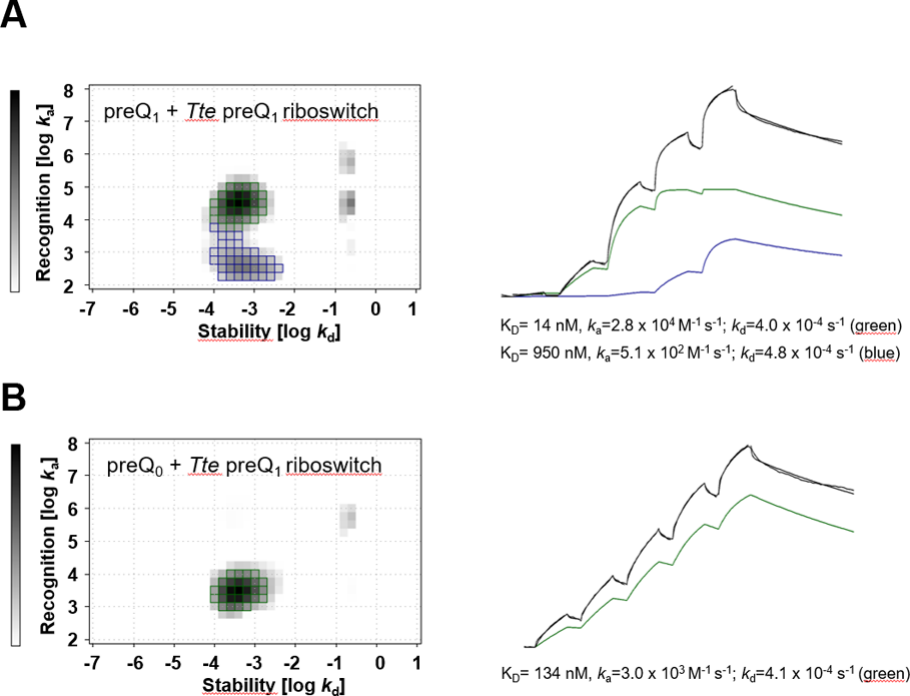
Interaction Map analyses of preQ_1_-*Tte* preQ_1_ riboswitch (A) and preQ_0_-*Tte* preQ_1_ riboswitch (B) interaction. The blue and the green spots represent the interactions with a peak weight of >40% corresponding to a true binding event (see text). On the right panel the calculated sensorgrams for the respective interaction are shown. The calculated affinity (*K*_D_), as well as the ON (*k*_on_) and OFF (*k*_off_) rates, are indicated below the sensorgram. The black curves represent experimental data and the summarized calculated data for the respective fit.

SPR sensorgrams of preQ_1_ binding to its riboswitch showed better fits when using a heterogeny target model compared to the standard 1:1 binding model. This deviates from our observations in ITC experiments of preQ_1_ binding to the preQ_1_ riboswitch (Figure S12) where only one binding event was observed with a molecular ratio of *n* ≈ 0.7 (Table S5). Binding stoichiometries of *n* < 1 in 1:1 binding events are usually observed when not all RNA (or protein) molecules are properly folded and thus not being capable of ligand binding. However, differences between ITC and SPR experiments might also originate from the fundamental differences between the two methods like the immobilization in SPR or high concentrations in ITC. Native polyacrylamide (PAGE) gel electrophoresis showed no evidence for RNA heterogeneity for the used constructs of the preQ_1_ riboswitch aptamers (Figure S24). To further elucidate potential binding heterogeneity, we performed interaction map (IM) analyses from the preQ_0_-preQ_1_ riboswitch and the preQ_1_-preQ_1_ riboswitch sensorgrams, respectively, in order to determine and quantify the individual binding events represented by the SPR curves. The algorithm splits the experimental SPR data set into several theoretical monovalent binding curves and selects the binding curves that, when summed up, best fit the experimental data. By plotting the association rate *k*_on_ and the dissociation rate *k*_off_ within a two-dimensional distribution, it is possible to display heterogeneous binding data as a map, in which each peak corresponds to one component that contributes to the cumulative binding curve.^92^ The IM data reveal a clear 2:1 interaction stoichiometry for preQ_1_-preQ_1_ riboswitch (Figure 6A), one more stable and with higher association rate (*K*_D_ = 15 nM, *k*_on_ = 2.8 × 10^4^ 1/Ms; *k*_off_ = 4.0 × 10^–4^ 1/s;) and one less stable interaction with a lower association rate (*K*_D_ = 950 nM, *k*_on_ = 5.1 × 10^2^ 1/Ms; *k*_off_ = 4.9 × 10^–4^ 1/s). Both interactions have a comparable peak weight revealing a clear 2:1 interaction. Hence, the observed 2:1 binding in SPR might not be caused by target heterogeneity but suggests that the *Tte* preQ_1_ riboswitch may have two binding sites for preQ_1_. The recently published crystal structure of the structurally similar *Carnobacterium antarcticum* preQ_1_ riboswitch showed two bound preQ_1_ molecules (Figure S26, PDB-ID: 8FB3).^93^ Probably, a similar second binding event is also possible in the *Tte* preQ_1_ riboswitch under SPR conditions. In contrast, the IM analyses of the preQ_0_-preQ_1_ riboswitch interaction revealed a 1:1 binding event with lower affinity (*K*_D_ = 134 nM, *k*_on_ = 3.1 × 10^3^ 1/Ms; *k*_off_ = 4.1 × 10^–4^ 1/s) compared to the more stable preQ_1_-preQ_1_ riboswitch interaction (Figure 6B).

#### Binding Thermodynamics

Thermodynamic parameters were determined by ITC. The natural ligands preQ_1_ and preQ_0_ showed thermodynamic binding profiles with large enthalpic contributions of −150 and −129 kJ/mol, respectively (Table 2). As described for the SAM-VI riboswitch, the highly exothermic binding is compensated by an entropic penalty of −*T*Δ*S* = +106 kJ/mol for preQ_1_ and *–T*Δ*S* = +88.5 kJ/mol for preQ_0_. The substitution of the nitrile group in preQ_0_ by the more flexible methylamino group in preQ_1_ resulted in slightly favorable enthalpic and unfavorable entropic contributions. The positively charged amine group is involved in specific polar interactions (Figure 4B), which might be accompanied by a potential rigidification upon binding. This is similar to the results obtained for the SAM-VI riboswitch where additional charges also led to more favorable binding enthalpy partially compensated by unfavorable entropy. These thermodynamic binding profiles differ from typical protein–ligand interactions, where enthalpic and entropic contributions are usually more balanced and potent ligands show larger increases in entropy from hydrophobic interactions and solvent displacement.^94^

#### Selectivity

For selectivity studies, binding of *Tte* preQ_1_ riboswitch ligands to the SAM-VI riboswitch were investigated using SPR and MST (Table 2). In MST, preQ_1_ and preQ_0_ were selective for the *Tte* preQ_1_ riboswitch showing no binding to the *Ba* SAM-VI riboswitch. Further, no binding was detected in steady-state evaluation of SPR. The sensorgrams of preQ_1_ showed partially fast-on/fast-off shapes and low response units (Figure S2B) similar to the ones of compound **2** against its off-target (Figure 5B) in contrast to the sensorgrams involving their targets (Figures S2B and 5A). The single positive charge of preQ_1_ probably increases the possibility of weak loose binding events in SPR. Dibenzofuran and carbazole derivatives **3**–**8** did not show binding in the MST assay (Figures S6C–H and S10C–H), but showed SPR sensorgram shapes of unspecific loose binding for both riboswitch aptamers(Figure S2C–H).

##### Conclusions

We investigated the addition of positive ligand charges as a design strategy in RNA-binding small molecule drug discovery. In this regard, the impact of additional ligand charges on binding affinity, kinetics, thermodynamics and selectivity was examined using the *Tte* preQ_1_ and *Ba* SAM-VI riboswitch aptamer domains as model systems. Combined methods of SPR, ITC and MST were used to examine the effects of protonated amines as “electrostatic anchors” on binding characteristics. For the SAM-VI riboswitch, the natural ligands SAM and SAH as well as synthetic positively charged SAM-analogs **1** and **2** were tested (Figure 3A, Table 1). For the *Tte* preQ_1_ riboswitch, preQ_1_ and preQ_0_ as natural binders (Figure 2A), and synthetic dibenzofuran and carbazole derivatives **3–8** (Figure 3B) were analyzed. Our results for reported natural ligands and derivatives thereof agree with previous findings,^63,67,70^ which validated the suitability of methods under investigation. Overall, the addition of positively charged moieties had only minor effects on the binding affinities and association rates (Tables 1 and 2). For both compound series, SAM, SAH, compounds **1** and **2,** and preQ_0_, preQ_1_, only a slight trend that a higher number of positive charges can lead to enhanced association rates, and thus affinity to the RNA targets, was observed. These changes were only moderate and cannot be considered as a universal design principle to overcome affinity cliffs.

Thermodynamic binding profiles of all ligands were dominated by enthalpy, partially compensated by unfavorable entropy terms. This observation was more pronounced for ligands carrying multiple positive charges. Like for protein–ligand interactions, this suggests that strong interactions like salt bridges reduce flexibility of interacting moieties for both ligand and target resulting in an entropic penalty. However, (de)solvation effects also play an important role in binding thermodynamics and may also strongly contribute to the observed thermodynamic binding profiles.^56,88,98^ As a consequence, this enthalpy–entropy compensation leads to smaller changes in Δ*G* and affinity than additional interaction sites might suggest. Compared to proteins, RNAs show highly charged polar surfaces. Therefore, the observed thermodynamic binding profiles are dominated by enthalpy and differ from common interactions between proteins and ligands were enthalpic and entropic contributions are more balanced.^94^ While during hit-to-lead optimization for protein ligands, entropy often becomes more favorable, this design principle might not be easily transferable to RNA-ligands.

In the selectivity study, all ligands of a particular target, SAM, SAH, **1** and **2** for the SAM-VI riboswitch, and preQ_1_ and preQ_0_ for the preQ_1_ riboswitch, distinguished their target from the respective off-target. Compounds **3**–**8** from the dibenzofuran and carbazole series showed no binding in ITC and MST. For these synthetic, putative preQ_1_ riboswitch ligands, however, fast association and dissociation rates were observed in SPR experiments for both riboswitches. The shapes of SPR sensorgrams and the steady-state analysis of SPR data showed clear differences for specific ligands compared to such loose binding behavior. Compounds, which did not bind in MST and are positively charged, had a higher probability to be identified as fast-on/fast-off loose binders^89^ in SPR. Therefore, a *caveat* arises for SPR-screenings against RNA-targets: Sensorgrams of loose and likely unspecific binders showed fast-off/fast-on rates, and no saturation of the target for steady-state analysis was reached. This was also the case for the SAM-VI riboswitch ligand **2** binding to the preQ_1_ riboswitch off-target (Figure 5B). We hypothesize this loose, unspecific binding is caused by high ligand charges leading to surface interactions between protonated amine moieties of the ligand with the phosphate-backbone of immobilized RNA. This behavior further suggests accumulation of the compounds on the target’s surface probably by electrostatic interactions like the “ion atmosphere” found around nucleic acids.^24−26^ For **3**–**8** this effect might be further enhanced by intercalation of the three-ring core scaffolds dibenzofuran or carbazole overshadowing the previously reported specific interactions^69,90^ with the preQ_1_ riboswitch. Similarly, increased potency by acridine-conjugation was described previously for multiple unrelated RNA-targets.^99,100^ However, such loose binding events to the off-targets probably might also be an artifact of immobilization-based techniques like SPR, as no binding was observed via MST and ITC for these compounds. Hence, it may not have biological relevance through RNA interaction competition. Polycationic compounds still can be selective for their target.^30,32,49,50,54^ Consequently, ligands carrying one or probably more positive charges are no exclusion criterium per se when it comes to binding affinity and selectivity. Still, negative pharmacokinetic effects on permeability and cellular uptake^64^ must be considered in RNA-targeting drug design. Subsequently, our findings demonstrate that introducing a positive ligand charge for specific interactions does not need to be generally avoided. The designed ligands of this study introduced positive charges mostly in molecular regions oriented away from the binding site. This aimed to minimize specific interactions with binding site residues and to elucidate the impact of long-range ionic interactions in sense of electrostatic anchoring or enhanced association in a Circe-like effect. Overall, the charge influence on affinity and association rates was low, but larger affinity gains can be obtained when placing direct ionic interactions or charge-assisted hydrogen bonds. An example can be found in the amine-substituted guanosine-derivative (Figure 2B) binding to the *tetrahymena* group I ribozyme with a 1000-fold affinity increase.^65^ Another example is the here and previously described discrimination of preQ_1_ riboswitches between preQ_1_ and preQ_0_.^63,101^ Within the series of SAM-derivatives, the biggest affinity increase was reached from SAH when exchanging neutral sulfur to a permanently charged sulfonium ion in SAM and partly when changed to a protonatable amine (compound **1**, Table 1). Even though no direct interaction is indicated by the SAM-VI riboswitch–SAM complex structure (Figure 4A), one could speculate if the flexibility of the amino acid substructure and the intrinsic flexibility of RNA are sufficient to occasionally form specific interactions between RNA and the sulfonium or ammonium groups. A likely interaction partner for a charge-assisted chalcogen bond with SAM as described previously for the SAM-I riboswitch,^76^ or ion-dipole interaction with compounds **1** and **2** would be oxygen (O4) of the U6 or U8 residues (Figure 4A). Further examples, where charged ligand moieties led to potent ligands are described for pre-miRNA-372^32^ (Figure 1A), the thiamine pyrophosphate (TPP) riboswitch,^102^ the hepatitis C virus internal ribosome entry site (HCV IRES)^103,104^ and myotonic dystrophy type 2 (DM2)-causing CUG-repeats.^46^

In summary, our kinetic and thermodynamic in vitro studies provide valuable insights into molecular recognition between RNA and small molecules to answer the initially raised questions: (1—affinity) The implementation of solvent exposed, positively charged moieties leads to only minor increases in potency insufficient to overcome affinity cliffs. Differently, purposefully placed moieties forming ion–ion or ion-dipole interactions can have a larger impact.^63,65,67,70^ (2—kinetics) Small to moderate improvements in affinity from ligand charges seem to arise from faster association rates agreeing with electrostatic anchoring and a Circe-like effect. (3—selectivity) No general loss of selectivity and off-target (*pan*-RNA) binding was observed with up to +3 formal charges (compounds **7**, **8**) in MST for our model systems. SPR sensorgrams showed fast-on/fast-off kinetics and loose unspecific binding of multibasic compounds. This behavior can be identified from the sensorgrams (fast-on/fast-off) while saturation in dose–response curves is not reached.^105^ Awareness and testing with orthogonal methods can protect against following-up false positive multicharged hits in SPR screenings against RNA. *Vice versa*, the implementation of a well-placed charged moiety does not impair selectivity per se. (4—thermodynamics). All thermodynamic binding profiles were dominated by high enthalpic contributions and unfavorable entropy. This enthalpy–entropy compensation is also well described for proteins where enthalpically strong ion–ion and ion-dipole interactions result in an entropic penalty.^56,88,98^ (5—design strategy) Taken together, implementation of positively charged groups cannot serve as a universal strategy for RNA-ligand design. The low contribution to affinity is insufficient to overcome affinity cliffs (1) even though association rates are slightly enhanced (2). However, a well-placed charged moiety could enhance potency without necessarily having negative impact on selectivity (3). Still, physicochemical properties and their effect on pharmacokinetics must be considered in hit to lead optimization. These findings contribute to a better understanding of RNA-small molecule recognition and provide a piece to the puzzle of how to design and optimize selective, drug-like RNA-ligands.

## Experimental Section

### General Synthetic Methods

All reagents and solvents were commercial grade and used without further purification. Reaction progress was monitored by thin layer chromatography (**TLC**) using Alugram Xtra F254 silica plates from Machery-Nagel. In addition, high-performance liquid chromatography/electron spray ionization mass spectrometry (**HPLC/ESI-MS**) was used to control reaction’s conversion and to determine the identity as well as the purity of compounds tested in the assay. An Agilent 1100 series HPLC system and either an Agilent Zorbax SB-Aq (4.6 mm × 150 mm, 5 μm) or Poroshell 120 EC-C18 (2.10 mm × 150 mm, 4 μM) column coupled to an Agilent 1100 series LC/MSD Trap with ESI, was used. The measurements were conducted with a gradient of acetonitrile and water (+0.1% formic acid) with 10–90% acetonitrile over 10 min with a flow rate of 0.7 mL/min unless otherwise stated. Signals were detected at 254 nm with quantification by area under curve (AUC) and masses were determined in positive ionization mode (ESI). For the determination of purity and identity for the most polar compounds **7** and **8**, a Waters Alliance e2695 HPLC system and a MZ-Aqua Perfect C18 (4.6 × 250 mm, 10 μm) analytical HPLC column coupled to a Waters ACQUITY QDa single quadrupole detector with ESI was used. **Column chromatography** was performed with silica gel 60 (40–63 μm) from Machery-Nagel. **Flash column chromatography** was performed with the Biotage Isolera One system. For reversed-phase flash chromatography prepacked columns of the type BiotageSfär C18 Duo from Biotage were used. In the case of normal-phase flash chromatography, columns were self-packed with silica gel 60 (15–40 μm) from Merck KGaA. **Preparative HPLC** purification was performed with a Varian PrepStar system (model 218) with a MZ-Aqua Perfect C18 20 × 250 mm, 7 μm preparative LC column and acetonitrile/water +0.1% trifluoroacetic acid as mobile phase. **Melting points (mp)** (uncorrected) were measured with a MPM-H3 device from Schorpp Gerätetechnik using semiopen capillaries. Proton (^1^H) and carbon (^13^C) nuclear magnetic resonance (**NMR**) spectra were recorded on Bruker Fourier 300 MHz (300 MHz for ^1^H and 75 MHz for ^13^C). The chemical shift was abbreviated to δ and has the unit ppm. The chemical shifts were referenced to the solvent peaks in ^1^H: δ = 7.26 (CDCl_3_), 2.50 (DMSO-*d*_6_), 3.31 (CD_3_OD) ppm and in ^13^C: δ = 77.16 (Chloroform-*d*), 39.52 (DMSO-*d*_6_), 49.00 (CD_3_OD) ppm purchased from Deutero GmbH. In addition, the following abbreviations for the multiplicities of the peaks were defined: s (singlet), d (doublet), dd (doublet of doublet), t (triplet), td (triplet of doublet), q (quartet), p (pentet), and m (multiplet). Coupling constant *J* was given in Hz and denoted together with the number of signaling hydrogen atoms for ^1^H NMR spectra. MestReNova 12.0.4-22023 NMR spectrum processing program from *Mestrelab Research* was used to evaluate the NMR spectra and to determine the purity of the compounds through LC–MS. The purity of all compounds tested in biochemical assays was ≥95% as determined by LC–MS. NMR spectra and LC–MS traces for the tested compounds were included in the SI (Figures S18–23).

#### General Method A: Molecular Weight Determination for Trifluoroacetate Salts

The molecular weights of trifluoroacetate salt compounds **5–8** (two benzofurans and two carbazoles) were determined using the corresponding LC system. Compounds **3d** and **4a** were used as reference for the respective compound class. First, the reference compound and the undetermined compound were mixed in a 1:1 ratio in methanol assuming the molecular weight of the free base. The percentual ratio of both compounds in the sample were evaluated by calculating the AUCs at 254 nm wavelength. The molecular weight of the tested compound was recalculated with the determined ratio. For confirmation, a new mixed 1:1 sample was prepared including the determined molecular weight. The ratio of the new determined AUCs at 254 nm had to be between 0.8 and 1.2. Different absorption properties of the reference compounds and the tested compounds at 254 nm were neglected. The aliphatic amine tail was assumed to not influence absorption at 254 nm wavelength. The hydrochloride salts of the final compounds **3** and **4** were evaluated, too, for further validation of this method and could confirm their molecular weights.

#### General Method B: Amide Couplings

The desired Boc-protected amino acid (1.20 equiv) and benzotriazol-1-yloxy(dimethylamino)methylidene]-dimethylazanium tetrafluoroborate (TBTU) (3.00 equiv) were suspended in DCM (0.6 M) and cooled to 0 °C under inert gas atmosphere. *N,N*-Di*iso*propylethylamine (DIPEA) (4.00 equiv) was added dropwise and the mixture was stirred for 1 h. The carbazole or benzofuran amine (1.00 equiv) was added portionwise and the mixture was allowed to reach room temperature and was stirred for 23 h. The reaction was quenched with saturated sodium bicarbonate solution. The aqueous layer was extracted with DCM unless otherwise stated. Combined organic layers were washed with aqueous saturated sodium chloride solution, dried over sodium sulfate, filtered, and concentrated under reduced pressure. Crude product was purified via column chromatography with either a cyclohexane-ethyl acetate or DCM-methanol system.

#### General Method C: Boc Deprotection

Deprotection was performed according to Han et al. reaction conditions.^106^ The Boc protected compound (1.00 equiv) was dissolved in 1,4-dioxane, treated with 4 M HCl in 1,4-dioxane (20.0 equiv) and stirred overnight under inert gas atmosphere. Ammonium salt precipitation was completed with cold petroleum ether and product isolation was done via vacuum filtration unless otherwise stated.

### Synthesis SAH Analogs **1** and **2**

#### (*S*)-2-Amino-4-((((2*R*,3*S*,4*R*,5*R*)-5-(6-amino-9*H*-purin-9-yl)-3,4-dihydroxytetrahydrofuran-2-yl)methyl)amino)butanoic Acid Trifluoroacetate Salt (**1**)

Complete synthesis and analytical data of SAH analog 1 was reported by Schwickert et al.^107^ Same batch was used. 99% purity.

#### (2*R*,3*R*,4*S*,5*R*)-2-(6-Amino-9*H*-purin-9-yl)-5-(((3-aminopropyl)-amino)methyl)tetrahydrofuran-3,4-diol Trifluoroacetate Salt (**2**)

Complete synthesis and analytical data of SAH analog **2** was reported by Schwickert et al.^107^ Same batch was used. 99% purity.

### Synthesis of Putative preQ_1_ Riboswitch Ligands Compounds **3**–**8** (Scheme 1)

#### 1-Dibenzofuran-2-ylethanone (**3a**)

Synthesis was performed according to Chiranjeevi et al.^108^ instructions. A solution of acetyl chloride (1.12 g, 1.02 mL, 14.3 mmol, 1.20 equiv) and anhydrous trichloroalumane (1.90 g, 14.2 mmol, 1.20 equiv) in chloroform (20.0 mL) was added dropwise to a stirred solution of dibenzofuran (2.00 g, 11.9 mmol, 1.00 equiv) in chloroform (20.0 mL) under inert gas atmosphere. The reaction mixture was stirred at room temperature for 4 h. The reaction mixture was poured over ice and the precipitated aluminum hydroxide was dissolved with concentrated hydrochloric acid. The aqueous layer was extracted with chloroform (2 × 20.0 mL). The combined organic layers were dried with sodium sulfate, filtered and concentrated under reduced pressure. The crude product was purified via column chromatography (cyclohexane:ethyl acetate; 5:1). Target compound **3a** (1.19 g, 5.66 mmol, 48% yield) was isolated as a colorless solid. Mp: 77–79 °C. ^1^H NMR (300 MHz, CDCl_3_): δ = 8.61–8.54 (m, 1H), 8.09 (dd, *J* = 8.6, 1.8 Hz, 1H), 8.03–7.94 (m, 1H), 7.62–7.54 (m, 2H), 7.54–7.45 (m, 1H), 7.42–7.33 (m, 1H), 2.70 (s, 3H). ^13^C NMR (75 MHz, CDCl_3_): δ = 197.4, 159.0, 157.0, 132.6, 128.1, 124.7, 123.9, 123.5, 121.7, 121.0, 112.1, 111.7, 26.9. MS (ESI): found: *m*/*z* = 211.0 [M + H^+^], calculated: *m*/*z* = 211.1 [M + H^+^].

#### Dibenzofuran-2-yl Acetate (**3b**)

Synthesis was performed according to Chiranjeevi et al.^108^ instructions. A solution of 1-dibenzofuran-2-ylethanone (**3a**) (1.60 g, 7.61 mmol, 1.00 equiv) in dry DCM (25.0 mL) was cooled to 0 °C under inert gas atmosphere. 2,2,2-trifluoroacetic acid (3.55 g, 2.40 mL, 31.2 mmol, 4.09 equiv) was added dropwise, followed by a solution of 3-chloroperoxybenzoic acid (3.41 g, 15.2 mmol, 2.00 equiv) in DCM (13.0 mL), which was added carefully. After addition, the mixture was allowed to reach room temperature and was stirred for 3 d. The reaction was quenched with aqueous FeSO_4_ heptahydrate (around 2.5 g) solution and basified with saturated sodium bicarbonate solution under gas evolution. Emerging solid was filtered over Celite and the filtrate was washed with saturated sodium bicarbonate solution. The aqueous layer was extracted with DCM (2× 25.0 mL) and the combined organic layers were washed with saturated sodium chloride solution, dried with sodium sulfate, filtered and concentrated under reduced pressure. The dark brown crude product was purified via column chromatography (cyclohexane:ethyl acetate; 7:1). The content of isomeric 1-(dibenzo[b,d]furan-3-yl)ethan-1-one was reduced by recrystallization from methanol (15 mL) from 10 to 3% isomeric 1-(dibenzo[b,d]furan-3-yl)ethan-1-one. The target compound **3b** (1.16 g, 4.95 mmol, 65% yield) was isolated as beige solid. Mp: 113–115 °C. ^1^H NMR (300 MHz, CDCl_3_): δ = 7.95–7.87 (m, 1H), 7.72–7.67 (m, 1H), 7.61–7.53 (m, 2H), 7.52–7.43 (m, 1H), 7.39–7.30 (m, 1H), 7.17 (dd, *J* = 8.8, 2.4 Hz, 1H), 2.36 (s, 3H). ^13^C NMR (75 MHz, CDCl_3_): δ = 170.1, 157.1, 153.8, 146.3, 127.8, 125.1, 124.1, 122.9, 121.0, 120.8, 113.7, 112.2, 112.0, 21.3. MS (ESI): found: *m*/*z* = 184.9 [M-Acetyl+H^+^], calculated: *m*/*z* = 227.1 [M+H^+^], *m*/*z* = 185.1 [M-Acetyl+H^+^].

#### 2-Dibenzofuranol (**3c**)

Synthesis was performed according to Chiranjeevi et al.^108^ instructions. A suspension of dibenzofuran-2-yl acetate (**3b**) (1.10 g, 4.86 mmol, 1.00 equiv) in methanol (19.0 mL) was added dropwise to a suspension of freshly prepared sodium methanolate in methanol (559 mg elementary sodium in 12.0 mL methanol) under ice cooling under inert gas atmosphere. The mixture was allowed to reach room temperature. After 1 h, the reaction was quenched with 2 M HCl solution (8.00 mL). Methanol was evaporated under reduced pressure and the remaining residue was taken in water. The aqueous layer was acidified with concentrated HCl to protonate the product. Then it was extracted with chloroform (3×) and the organic layer was washed with saturated sodium chloride solution, dried with sodium sulfate, filtered and concentrated under reduced pressure. The target compound **3c** (876 mg, 4.75 mmol, 98% yield) was isolated as pale-yellow solid. Mp: 130–132 °C. ^1^H NMR (300 MHz, CDCl_3_): δ = 7.91–7.84 (m, 1H), 7.55 (dt, *J* = 8.3, 0.9 Hz, 1H), 7.49–7.40 (m, 2H), 7.40–7.35 (m, 1H), 7.35–7.28 (m, 1H), 6.97 (dd, *J* = 8.8, 2.6 Hz, 1H), 4.95 (s, 1H). ^13^C NMR (75 MHz, CDCl_3_): δ = 127.4, 125.2, 124.3, 122.6, 120.8, 115.4, 112.3, 111.9, 106.4. MS (ESI): found: *m*/*z* = 184.9 [M + H^+^], calculated: *m*/*z* = 185.1 [M + H^+^].

#### 2-Methoxydibenzofuran (**3d**)

Synthesis was performed according to Yempala et al.^109^ instructions. Sodium hydride (15.6 mg, 391 μmol, 1.20 equiv) was added to a solution of 2-dibenzofuranol (**3c**) (60.0 mg, 326 μmol, 1.00 equiv) in dry THF (1 mL) under ice cooling and inert gas atmosphere and stirred for 10 min. After, iodomethane (55.5 mg, 24.3 μL, 391 μmol, 1.20 equiv) was added and was allowed to reach room temperature and was stirred for 1 h. Further 0.60 equiv iodomethane were added and stirred for 1 h. The reaction was quenched with 5 mL water and the aqueous layer was extracted with DCM (3× 15.0 mL). Combined organic layers were washed with aqueous saturated sodium chloride solution, dried over sodium sulfate, filtered, and concentrated under reduced pressure. The crude product was purified via flash chromatography (cyclohexane:ethyl acetate; gradient 0–100% ethyl acetate) and was applied as dry load. The target compound **3d** (23.7 mg, 118 μmol, 36% yield) was isolated as pink solid. Mp: 47–49 °C. ^1^H NMR (300 MHz, CDCl_3_): δ = 7.96–7.88 (m, 1H), 7.56 (dt, *J* = 8.3, 1.0 Hz, 1H), 7.51–7.39 (m, 3H), 7.33 (td, *J* = 7.5, 1.1 Hz, 1H), 7.06 (dd, *J* = 8.9, 2.6 Hz, 1H), 3.92 (s, 3H). ^13^C NMR (75 MHz, CDCl_3_): δ = 157.1, 156.0, 151.0, 127.2, 124.8, 124.6, 122.5, 120.7, 115.3, 112.2, 111.9, 103.9, 56.2. MS (ESI). found: *m*/*z* = 199.0 [M + H^+^], calculated: *m*/*z* = 199.1 [M + H^+^].

#### 3-Methoxy-9*H*-carbazole (**4a**)

Synthesis was performed according to Ku et al.^110^ instructions. A solution of 3-bromo-9*H*-carbazole (2.00 g, 8.13 mmol, 1.00 equiv) in dry DMF (11.0 mL) was added to a suspension of freshly prepared sodium methanolate (3.10 g elementary sodium in 20.0 mL methanol; excess of methanol was removed under reduced pressure) in dry DMF (20.0 mL). Copper(I)iodide (3.10 g, 16.3 mmol, 2.00 equiv) was added and the mixture was stirred for 15 min at room temperature while several color changes were observed. Afterward, the dark green mixture was heated to 120 °C and refluxed for 22 h under inert gas atmosphere. The reaction mixture was filtered over silica gel, which was rinsed several times with ethyl acetate (in total 150 mL). The organic phase was washed with water and the resulting precipitate was filtered off over silica gel. The filtrate was washed with saturated sodium chloride solution (3× 30.0 mL, 5× 10.0 mL) and the organic layer was dried with sodium sulfate, filtered and concentrated under reduced pressure. The brown crude product was purified via column chromatography (cyclohexane:ethyl acetate; 5:1). The target compound **4a** (1.36 g, 6.88 mmol, 85% yield) was isolated as beige solid. Mp: 145–147 °C. ^1^H NMR (300 MHz, CDCl_3_): δ = 8.07 (dt, *J* = 7.8, 1.0 Hz, 1H), 7.88 (s, 1H), 7.60 (d, *J* = 2.5 Hz, 1H), 7.49–7.36 (m, 2H), 7.32 (dd, *J* = 8.8, 0.6 Hz, 1H), 7.29–7.21 (m, 1H), 7.10 (dd, *J* = 8.8, 2.5 Hz, 1H), 3.96 (s, 3H). ^13^C NMR (75 MHz, CDCl_3_): δ = 154.0, 140.4, 134.5, 125.9, 123.9, 123.5, 120.4, 119.2, 115.2, 111.4, 110.9, 103.3, 56.2. MS (ESI): found: *m*/*z* = 198.0 [M+H^+^], calculated: *m*/*z* = 198.1 [M+H^+^].

#### 9*H*-Carbazol-3-ol (**4b**)

Synthesis was performed according to Milne et al.^111^ instructions. A mixture of 3-methoxy-9*H*-carbazole (**4a**) (1.30 g, 6.59 mmol, 1.00 equiv), 47% hydrobromic acid (2.90 mL) and acetic acid glacial (24.0 mL) was heated to reflux (at 135 °C) under inert gas atmosphere. The progress of the reaction was monitored by TLC every hour. After 6 h, the reaction mixture was cooled to room temperature and acetone (20.0 mL) and water (10.0 mL) were added. The aqueous layer was extracted with DCM (1× 20.0 mL, 4× 10.0 mL) and concentrated under reduced pressure. The residue was taken in a water/acetonitrile mixture and lyophilized overnight. The crude product was purified via column chromatography (cyclohexane:ethyl acetate; 5:1 to 2:1). Traces of starting material were removed by washing the solid with chloroform (3× 3.00 mL). The target compound **4b** (956 mg, 5.22 mmol, 79% yield) was isolated as pale green solid. Mp: > 255 °C under decomposition. ^1^H NMR (300 MHz, DMSO-*d*_6_): δ = 10.87 (s, 1H), 8.92 (s, 1H), 7.98 (d, *J* = 7.8 Hz, 1H), 7.47–7.36 (m, 2H), 7.36–7.26 (m, 2H), 7.15–7.00 (m, 1H), 6.91 (dd, *J* = 8.6, 2.4 Hz, 1H). ^13^C NMR (75 MHz, DMSO-*d*_6_): δ = 150.4, 140.4, 133.8, 125.2, 123.1, 122.3, 120.1, 117.7, 115.0, 111.3, 110.8, 104.9. MS (ESI): found: *m*/*z* = 183.8 [M+H^+^], calculated: *m*/*z* = 184.1 [M+H^+^].

Synthesis of **5a** and **6a** was performed according to Pájaro et al.^112^ reaction conditions. 2-Dibenzofuranol (**3c**) or 9*H*-carbazol-3-ol (**4b**) (100 mg, 1.00 equiv) was added portionwise to a suspension of 1,2-dibromoethane (5.00 equiv) and anhydrous potassium carbonate (3.00 equiv) in dry acetonitrile (2 mL) heated at 80 °C under inert gas atmosphere. After addition, the mixture was heated to 80 °C and stirred for 22–24 h. The mixture was filtered and the filtrate was concentrated under reduced pressure. Crude product was purified via column chromatography (cyclohexane:ethyl acetate).

#### 2-(2-Bromoethoxy)dibenzofuran (**5a**)

Crude product was purified via column chromatography (cyclohexane:ethyl acetate; 20:1) yielding **5a** (96.4 mg, 318 μmol, 59% yield) as colorless solid. Mp: 69–71 °C. ^1^H NMR (300 MHz, CDCl_3_): δ = 7.94–7.87 (m, 1H), 7.56 (dt, *J* = 8.3, 0.9 Hz, 1H), 7.50–7.42 (m, 3H), 7.37–7.30 (m, 1H), 7.07 (dd, *J* = 8.9, 2.7 Hz, 1H), 4.38 (t, *J* = 6.3 Hz, 2H), 3.69 (t, *J* = 6.3 Hz, 2H). ^13^C NMR (75 MHz, CDCl_3_): δ = 157.1, 154.4, 151.5, 127.4, 124.9, 124.4, 122.7, 120.7, 116.0, 112.4, 111.9, 105.7, 69.2, 29.4. MS (ESI): found: not ionizable, calculated: *m*/*z* = 291.0 [M + H^+^].

#### 3-(2-Bromoethoxy)-9*H*-carbazole (**6a**)

Crude product was purified via column chromatography (cyclohexane:ethyl acetate; 4:1) yielding **6a** (81.9 mg, 282 μmol, 52% yield) as colorless solid. Mp: 134–136 °C. ^1^H NMR (300 MHz, DMSO-*d*_6_): δ = 11.08 (s, 1H), 8.15–8.05 (m, 1H), 7.74 (d, *J* = 2.5 Hz, 1H), 7.53–7.31 (m, 3H), 7.17–7.04 (m, 2H), 4.40 (t, *J* = 5.5 Hz, 2H), 3.84 (t, *J* = 5.5 Hz, 2H). ^13^C NMR (75 MHz, DMSO-*d*_6_): δ = 151.5, 140.5, 134.9, 125.5, 122.8, 122.4, 120.3, 118.1, 115.3, 111.6, 111.0, 104.8, 68.7, 31.8. MS (ESI): found: *m*/*z* = 289.8/291.8 [M + H^+^], calculated: *m*/*z* = 290.0/292.0 [M + H^+^].

Synthesis of **5** and **6** was performed according to Zhang et al.^113^ reaction conditions. A solution of propane-1,3-diamine (10.0 equiv) in acetonitrile (3 M) was prepared and heated to 80 °C. Then a solution of 2-(2-bromoethoxy)dibenzofuran (**5a**) or 3-(2-bromoethoxy)-9H-carbazole (**6a**) (1.00 equiv) in acetonitrile (1 M) was added dropwise under inert gas atmosphere. After addition, the mixture was stirred at that temperature for 3 h. The reaction mixture was concentrated under reduced pressure. The crude product was purified via preparative HPLC (gradient 10–100% acetonitrile in water+0.1% TFA, eluates at 40–50%). The molecular weight was determined according to general method A.

#### *N*^1^-(2-(Dibenzo[b,d]furan-2-yloxy)ethyl)propane-1,3-diamine Trifluoroacetate Salt (**5**)

**5a** (35.0 mg, 120 μmol, 1.00 equiv) yielded the target compound **5** as colorless solid (600.4 g/mol (∼3× TFA), 40.4 mg, 0.067 mmol, 49.0% yield). Mp: 179–181 °C. ^1^H NMR (300 MHz, DMSO-*d*_6_): δ = 8.10 (d, *J* = 7.7 Hz, 1H), 7.76 (s, 1H), 7.64 (t, *J* = 7.9 Hz, 2H), 7.50 (t, *J* = 7.8 Hz, 1H), 7.38 (t, *J* = 7.6 Hz, 1H), 7.17 (d, *J* = 8.9 Hz, 1H), 4.43–4.28 (m, 2H), 3.43–3.39 (m, 2H), 3.23–3.07 (m, 2H), 3.02–2.85 (m, 2H), 2.12–1.90 (m, 2H). ^13^C NMR (75 MHz, DMSO-*d*_6_): δ = 156.4, 154.3, 150.6, 127.9, 124.4, 123.9, 123.1, 121.3, 116.3, 112.5, 111.9, 105.7, 64.4, 46.4, 44.6, 36.4, 23.9. MS (ESI): found: *m*/*z* = 285.1 [M+H^+^], calculated: *m*/*z* = 285.2 [M+H^+^]. 99% purity.

#### *N*^1^-(2-((9*H*-Carbazol-3-yl)oxy)ethyl)propane-1,3-diamine Trifluoroacetate Salt (**6**)

**6a** (76.0 mg, 262 μmol, 1.00 equiv) yielded the target compound **6** as colorless solid (855.8 g/mol (∼5× TFA), 38.5 mg, 0.045 mmol, 17% yield). Mp: >194 °C under decomposition. ^**1**^**H NMR** (300 MHz, DMSO-*d*_6_): δ = 11.20 (s, 1H), 8.08 (d, *J* = 7.7 Hz, 1H), 7.75 (d, *J* = 2.4 Hz, 1H), 7.45 (t, *J* = 8.5 Hz, 2H), 7.36 (t, *J* = 7.5 Hz, 1H), 7.17–7.05 (m, 2H), 4.33 (t, *J* = 4.9 Hz, 2H), 3.59–3.26 (m, 2H), 3.15 (t, *J* = 7.6 Hz, 2H), 2.94 (t, *J* = 7.5 Hz, 2H), 2.01 (p, *J* = 7.5 Hz, 2H). ^**13**^**C NMR** (75 MHz, DMSO-*d*_6_): δ = 151.8, 140.9, 135.5, 126.0, 123.2, 122.8, 120.6, 118.5, 115.8, 112.1, 111.5, 105.1, 64.8, 46.7, 44.8, 36.7, 24.2. **MS (ESI):** found: *m*/*z* = 284.1 [M + H^+^], calculated: *m*/*z* = 284.2 [M + H^+^]. 100% purity.

#### *tert*-Butyl *N*-(2-Bromoethyl) Carbamate (**3e**)

A solution of di-*tert*-butyldicarbonate (3.20 g, 14.6 mmol, 1.00 equiv) in DCM (10.0 mL) was added dropwise to a solution of 2-bromoethylamine hydrobromide (3.00 g, 14.6 mmol, 1.00 equiv) triethylamine (1.48 g, 2.03 mL, 14.6 mmol, 1.00 equiv) in DCM (30.0 mL) under ice cooling and inert gas atmosphere. After addition, the mixture was allowed to reach room temperature and was stirred for 2 h. The organic phase was washed with 1 M HCl (3× 20.0 mL), saturated sodium bicarbonate solution (3× 20.0 mL), aqueous saturated sodium chloride solution (1× 20 mL), dried over sodium sulfate, filtered, and concentrated under reduced pressure. The crude product of **3e** (2.967 g) showed around 20% di-*tert*-butyldicarbonate contamination and was used without further purification.^**1**^**H NMR** (300 MHz, CDCl_3_): δ = 4.99 (s, 1H), 3.57–3.46 (m, 2H), 3.46–3.38 (m, 2H), 1.43 (s, 9H). ^**13**^**C NMR** (75 MHz, CDCl_3_): δ = 155.7, 79.9, 42.5, 32.8, 28.4. **MS (ESI):** found: *m*/*z* = 167.7/169.8 [M-*tert*-butyl+H^+^], calculated: *m*/*z* = 224.0/226.0 [M+H^+^], *m*/*z* = 168.0/170.0 [M-*tert*-butyl+H^+^].

Synthesis of **3f** and **4c** was performed according to Pájaro et al.^112^ reaction conditions. 2-Dibenzofuranol (**3c**) or 9*H*-carbazol-3-ol (**4b**) (1.00 equiv), *tert*-butyl *N*-(2-bromoethyl)carbamate (**3e**) (3.00 equiv, 80% purity) and anhydrous potassium carbonate (5.00 equiv) were mixed in dry acetonitrile (**3c/4b** 0.3 M). The mixture was heated at 80 °C and stirred for 18–20 h. The mixture was concentrated under reduced pressure. The residue was taken in water and was extracted with DCM. Combined organic layers were washed with water and aqueous saturated sodium chloride solution, dried over sodium sulfate, filtered, and concentrated under reduced pressure. The crude product was purified via flash chromatography or column chromatography.

#### *tert*-Butyl (2-(Dibenzo[b,d]furan-2-yloxy)ethyl)carbamate (**3f**)

**3c** (750 mg, 3.26 mmol, 1.00 equiv) was used. Crude product was purified via flash chromatography (cyclohexane:ethyl acetate; gradient 5–12% ethyl acetate). Mixed product fractions with contamination of *tert*-butyl *N*-(2-bromoethyl)carbamate were purified via column chromatography (cyclohexane:ethyl acetate; 7:1) yielding target compound **3f** as pale-yellow oil (425 mg, 1.30 mmol, 40% yield). ^1^H NMR (300 MHz, CDCl_3_): δ = 7.79–7.72 (m, 1H), 7.45–7.27 (m, 3H), 7.24 (s, 1H), 7.23–7.12 (m, 1H), 6.89 (dd, *J* = 8.9, 2.6 Hz, 1H), 5.03 (s, 1H), 3.97 (t, *J* = 5.2 Hz, 2H), 3.47 (d, *J* = 5.7 Hz, 2H), 1.37 (s, 9H). ^13^C NMR (75 MHz, CDCl_3_): δ = 157.0, 156.0, 154.8, 151.1, 127.2, 124.8, 124.4, 122.5, 120.6, 115.6, 112.2, 111.8, 104.8, 79.6, 68.2, 40.4, 28.5. MS (ESI): found: *m*/*z* = 350.1 [M + Na^+^], calculated: *m*/*z* = 328.2 [M + H^+^], *m*/*z* = 350.1 [M + Na^+^].

#### *tert*-Butyl (2-((9*H*-Carbazol-3-yl)oxy)ethyl)carbamate (**4c**)

**4b** (642 mg, 2.80 mmol, 1.00 equiv) was used. The crude product was purified via column chromatography (cyclohexane:ethyl acetate; 3:1). The target compound **4c** (350 mg, 1.07 mmol, 38% yield) was isolated as yellow solid. Mp: 154–158 °C. ^1^H NMR (300 MHz, DMSO-*d*_6_): δ = 11.01 (s, 1H), 8.13–8.03 (m, 1H), 7.69 (d, *J* = 2.5 Hz, 1H), 7.47–7.30 (m, 3H), 7.14–7.07 (m, 1H), 7.03 (dd, *J* = 8.7, 2.5 Hz, 1H), 4.05 (t, *J* = 5.9 Hz, 2H), 3.40–3.31 (m, 2H), 1.40 (s, 9H). ^13^C NMR (75 MHz, DMSO-*d*_6_): δ = 155.7, 152.0, 140.4, 134.7, 125.4, 122.8, 122.4, 120.2, 118.0, 115.3, 111.5, 110.9, 104.2, 77.7, 67.3, 39.7, 28.2. MS (ESI): found: *m*/*z* = 227.0 [M-Boc + H^+^], calculated: *m*/*z* = 327.2 [M + H^+^], *m*/*z* = 227.1 [M-Boc + H^+^].

#### 2-Dibenzofuran-2-yloxyethylamine Hydrochloride (**3**)

Synthesis was performed according to general method C. **3f** (1.73 g, 5.29 mmol, 1.00 equiv) was used. The target compound **3** (1.31 g, 4.97 mmol, 94% yield) was isolated as colorless solid. Mp: > 260 °C. ^1^H NMR (300 MHz, DMSO-*d*_6_): δ = 8.45 (s, 3H), 8.15 (d, *J* = 7.6 Hz, 1H), 7.81 (d, *J* = 2.6 Hz, 1H), 7.65 (t, *J* = 8.5 Hz, 2H), 7.51 (t, *J* = 7.7 Hz, 1H), 7.38 (t, *J* = 7.4 Hz, 1H), 7.17 (dd, *J* = 9.0, 2.7 Hz, 1H), 4.32 (t, *J* = 5.2 Hz, 2H), 3.25 (s, 2H). ^13^C NMR (75 MHz, DMSO-*d*_6_): δ = 156.2, 154.3, 150.3, 127.6, 124.2, 123.8, 122.9, 121.3, 116.3, 112.3, 111.7, 105.5, 65.2, 38.3. MS (ESI): found: *m*/*z* = 228.0 [M + H^+^], calculated: *m*/*z* = 228.1 [M + H^+^]. 100% purity.

#### 2-((9*H*-Carbazol-3-yl)oxy)ethan-1-amine Hydrochloride (**4**)

Synthesis was performed according to general method C. **4c** (1.02 g, 3.13 mmol, 1.00 equiv) was used. The target compound **4** (629 mg, 2.39 mmol, 76% yield) was isolated as colorless solid. Mp: >260 °C. ^1^H NMR (300 MHz, DMSO-*d*_6_): δ = 11.21 (s, 1H), 8.42 (s, 3H), 8.09 (d, *J* = 7.8 Hz, 1H), 7.75 (d, *J* = 2.4 Hz, 1H), 7.50–7.38 (m, 2H), 7.41–7.29 (m, 1H), 7.17–7.05 (m, 2H), 4.29 (t, *J* = 5.2 Hz, 2H), 3.24 (q, *J* = 5.3 Hz, 2H). ^13^C NMR (75 MHz, DMSO-*d*_6_): δ = 151.4, 140.4, 135.0, 125.5, 122.7, 122.4, 120.3, 118.1, 115.5, 111.7, 111.1, 104.6, 65.2, 38.5. MS (ESI): found: *m*/*z* = 227.0 [M + H^+^], calculated: *m*/*z* = 227.1 [M + H^+^]. 100% purity.

#### *tert*-Butyl (3-((2-(Dibenzo[b,d]furan-2-yloxy)ethyl)amino)-3-oxopropyl)carbamate (**7a**)

Synthesis was performed according to general method B. **3** (377 mg, 1.43 mmol, 1.00 equiv) was used. Crude product was purified via column chromatography (cyclohexane:ethyl acetate; 1:2). The target compound **7a** (485 mg, 1.22 mmol, 85% yield) was isolated as colorless solid. Mp: 117–119 °C. ^1^H NMR (300 MHz, CDCl_3_): δ = 7.92–7.85 (m, 1H), 7.57–7.38 (m, 4H), 7.31 (td, *J* = 7.5, 1.1 Hz, 1H), 7.01 (dd, *J* = 8.9, 2.6 Hz, 1H), 6.33 (s, 1H), 5.18 (s, 1H), 4.12 (t, *J* = 5.1 Hz, 2H), 3.70 (q, *J* = 5.3 Hz, 2H), 3.42 (t, *J* = 6.1 Hz, 2H), 2.52–2.38 (m, 2H), 1.41 (s, 9H). ^13^C NMR (75 MHz, CDCl_3_): δ = 171.8, 157.0, 156.2, 154.7, 151.2, 127.4, 124.9, 124.4, 122.6, 120.7, 115.6, 112.3, 111.9, 105.0, 79.5, 67.7, 39.2, 36.9, 36.4, 28.5. MS (ESI): found: *m*/*z* = 299.1 [M-Boc + H^+^], calculated: *m*/*z* = 399.2 [M + H^+^], *m*/*z* = 299.1 [M-Boc + H^+^].

#### *tert*-Butyl (3-((2-((9*H*-Carbazol-3-yl)oxy)ethyl)amino)-3-oxopropyl)carbamate (**8a**)

Synthesis was performed according to general method B. **6** (319 mg, 1.21 mmol, 1.00 equiv) was used. Crude product was purified via column chromatography (cyclohexane:ethyl acetate; 1:2). The target compound **8a** (430 mg, 920 μmol, 76% yield) was isolated as colorless solid. Mp: 149–152 °C.^1^H NMR (300 MHz, DMSO-*d*_6_): δ = 11.02 (s, 1H), 8.15 (t, *J* = 5.5 Hz, 1H), 8.08 (d, *J* = 7.8 Hz, 1H), 7.70 (d, *J* = 2.5 Hz, 1H), 7.48–7.29 (m, 3H), 7.16–6.99 (m, 2H), 6.74 (t, *J* = 5.8 Hz, 1H), 4.07 (t, *J* = 5.8 Hz, 2H), 3.48 (q, *J* = 5.7 Hz, 2H), 3.24–3.10 (m, 2H), 2.30 (t, *J* = 7.3 Hz, 2H), 1.37 (s, 9H). ^13^C NMR (75 MHz, DMSO-*d*_6_): δ = 170.7, 155.5, 152.0, 140.4, 134.7, 125.4, 122.8, 122.4, 120.3, 118.0, 115.3, 111.6, 111.0, 104.3, 77.6, 67.2, 38.4, 36.8, 35.8, 28.2. MS (ESI): found: *m*/*z* = 298.1 [M-Boc+H^+^], calculated: *m*/*z* = 398.2 [M + H^+^], *m*/*z* = 298.2 [M-Boc + H^+^].

#### 3-Amino-*N*-(2-(dibenzo[b,d]furan-2-yloxy)ethyl)propenamide Hydrochloride (**7b**)

Synthesis was performed according to general method C. **7a** (453 mg, 1.14 mmol, 1.00 equiv) was used and the reaction was stirred for 1.5 h. The solvent was removed under reduced pressure and the residue was lyophilized in water. The target compound **7b** (379 mg, 1.13 mmol, quantitative) was isolated as colorless solid. Mp: 171–173 °C. ^1^H NMR (300 MHz, DMSO-*d*_6_): δ = 8.54 (t, *J* = 5.5 Hz, 1H), 8.22–8.07 (m, 4H), 7.75 (d, *J* = 2.7 Hz, 1H), 7.66–7.55 (m, 2H), 7.53–7.44 (m, 1H), 7.36 (td, *J* = 7.5, 1.0 Hz, 1H), 7.10 (dd, *J* = 8.9, 2.7 Hz, 1H), 4.11 (t, *J* = 5.7 Hz, 2H), 3.51 (q, *J* = 5.7 Hz, 2H), 3.00 (t, *J* = 7.1 Hz, 2H), 2.59 (t, *J* = 7.1 Hz, 2H). ^13^C NMR (75 MHz, DMSO-*d*_6_): δ = 169.8, 156.2, 154.8, 150.1, 127.5, 124.2, 123.9, 122.8, 121.3, 116.0, 112.2, 111.7, 105.3, 67.1, 39.0, 38.4, 35.3, 32.1. MS (ESI): found: *m*/*z* = 299.0 [M + H^+^], calculated: *m*/*z* = 298.7 [M + H^+^].

#### *N*-(2-((9*H*-Carbazol-3-yl)oxy)ethyl)-3-aminopropanamide Hydrochloride (**8b**)

Synthesis was performed according to general method C. **8a** (400 mg, 1.01 mmol, 1.00 equiv) was used. The solvent was removed under reduced pressure and the residue was lyophilized in water. The target compound **8b** (318 mg, 953 μmol, 95% yield) was isolated as colorless solid. Mp: 175–177 °C. ^1^H NMR (300 MHz, DMSO-*d*_6_): δ = 11.16 (s, 1H), 8.51 (t, *J* = 5.6 Hz, 1H), 8.20–8.02 (m, 4H), 7.70 (d, *J* = 2.4 Hz, 1H), 7.49–7.29 (m, 3H), 7.14–6.99 (m, 2H), 4.09 (t, *J* = 5.7 Hz, 2H), 3.51 (q, *J* = 5.7 Hz, 2H), 3.01 (q, *J* = 6.3 Hz, 2H), 2.59 (t, *J* = 7.1 Hz, 2H). ^13^C NMR (75 MHz, DMSO-*d*_6_): δ = 169.7, 151.9, 140.4, 134.7, 125.4, 122.8, 122.4, 120.3, 118.0, 115.3, 111.6, 111.0, 104.2, 67.1, 38.5, 35.3, 32.1. MS (ESI): found: *m*/*z* = 298.1 [M + H^+^], calculated: *m*/*z* = 298.2 [M + H^+^].

#### *tert*-Butyl (4-((3-((2-(Dibenzo[b,d]furan-2-yloxy)ethyl)amino)-3-oxopropyl)amino)-4-oxobutyl)carbamate (**7c**)

Synthesis was performed according to general method B. **7b** (330 mg, 986 μmol, 1.00 equiv) was used. Crude product was purified via column chromatography (DCM:methanol; 20:1). The target compound **7c** (428 mg, 885 μmol, 90% yield) was isolated as colorless solid. Mp: 119–121 °C. ^1^H NMR (300 MHz, CDCl_3_): δ = 7.90–7.84 (m, 1H), 7.54–7.36 (m, 4H), 7.33–7.25 (m, 1H), 6.99 (dd, *J* = 8.9, 2.6 Hz, 1H), 6.95–6.72 (m, 2H), 4.91 (s, 1H), 4.10 (t, *J* = 5.2 Hz, 2H), 3.67 (q, *J* = 5.4 Hz, 2H), 3.52 (q, *J* = 5.9 Hz, 2H), 3.07 (t, *J* = 6.7 Hz, 2H), 2.47 (t, *J* = 6.1 Hz, 2H), 2.16 (t, *J* = 7.1 Hz, 2H), 1.74 (t, *J* = 6.9 Hz, 2H), 1.40 (s, 9H). ^13^C NMR (75 MHz, CDCl_3_): δ = 173.1, 172.0, 157.0, 156.4, 154.7, 151.2, 127.4, 124.9, 124.3, 122.6, 120.6, 115.6, 112.3, 111.8, 105.0, 79.4, 67.6, 39.2, 35.8, 33.7, 28.5, 26.2. MS (ESI): found: *m*/*z* = 484.1 [M + H^+^], calculated: *m*/*z* = 484.2 [M + H^+^].

#### *tert*-Butyl (4-((3-((2-((9*H*-Carbazol-3-yl)oxy)ethyl)amino)-3-oxopropyl)amino)-4-oxobutyl)carbamate (**8c**)

Synthesis was performed according to general method B. **8b** (279 mg, 836 μmol, 1.00 equiv) was used. The aqueous layer was extracted with DCM (1× 20.0 mL). Next, a mixture of chloroform and *iso*propanol (2:1) was used to extract the aqueous phase (3× 20.0 mL). Combined organic layers were washed with aqueous saturated sodium chloride solution, dried over sodium sulfate, filtered, and concentrated under reduced pressure. Crude product was purified via column chromatography (DCM:methanol; 15:1). The target compound **8c** (359 mg, 744 μmol, 89% yield) was isolated as colorless/pale yellow solid. Mp: 160–163 °C. ^1^H NMR, (300 MHz, DMSO-*d*_6_): δ = 11.02 (s, 1H), 8.16 (t, *J* = 5.6 Hz, 1H), 8.08 (d, *J* = 7.8 Hz, 1H), 7.84 (t, *J* = 5.7 Hz, 1H), 7.70 (d, *J* = 2.4 Hz, 1H), 7.48–7.29 (m, 3H), 7.15–7.00 (m, 2H), 6.77 (t, *J* = 5.7 Hz, 1H), 4.08 (t, *J* = 5.8 Hz, 2H), 3.49 (q, *J* = 5.7 Hz, 2H), 3.34–3.21 (m, 2H), 2.89 (q, *J* = 6.6 Hz, 2H), 2.31 (t, *J* = 7.0 Hz, 2H), 2.04 (t, *J* = 7.5 Hz, 2H), 1.58 (p, *J* = 7.3 Hz, 2H), 1.36 (s, 9H). ^13^C NMR (75 MHz, DMSO-*d*_6_): δ = 171.9, 170.8, 155.6, 152.0, 140.4, 134.7, 125.4, 122.8, 122.5, 120.3, 118.0, 115.3, 111.6, 111.0, 104.3, 77.4, 67.2, 39.5, 38.5, 35.4, 35.3, 32.9, 28.3, 25.9. MS (ESI): found: *m*/*z* = 483.1 [M + H^+^], calculated: *m*/*z* = 483.3 [M + H^+^].

#### 4-Amino-*N*-(3-((2-(dibenzo[b,d]furan-2-yloxy)ethyl)amino)-3-oxopropyl)butanamide Hydrochloride (**7d**)

Synthesis was performed according to general method C. **7c** (100 mg, 207 μmol, 1.00 equiv) was used and the reaction was stirred for 1 h. Solvent was concentrated under reduced pressure and the residue was lyophilized in water. The target compound **7d** (86.0 mg, 205 μmol, quantitative) was isolated as colorless solid. Mp: 206–209 °C. ^1^H NMR (300 MHz, DMSO-*d*_6_): δ = 8.28 (t, *J* = 5.6 Hz, 1H), 8.19–8.04 (m, 5H), 7.75 (d, *J* = 2.6 Hz, 1H), 7.61 (dd, *J* = 16.3, 8.6 Hz, 2H), 7.54–7.43 (m, 1H), 7.36 (t, *J* = 7.5 Hz, 1H), 7.10 (dd, *J* = 9.0, 2.7 Hz, 1H), 4.10 (t, *J* = 5.8 Hz, 2H), 3.48 (q, *J* = 5.7 Hz, 2H), 3.28 (q, *J* = 6.7 Hz, 2H), 2.75 (t, *J* = 7.5 Hz, 2H), 2.32 (t, *J* = 7.1 Hz, 2H), 2.18 (t, *J* = 7.2 Hz, 2H), 1.78 (p, *J* = 7.3 Hz, 2H). ^13^C NMR (75 MHz, DMSO-*d*_6_): δ = 171.3, 170.8, 156.1, 154.8, 150.1, 127.5, 124.2, 123.9, 122.8, 121.2, 116.0, 112.1, 111.6, 105.3, 67.2, 38.4, 38.3, 35.4, 35.3, 32.1, 23.2. MS (ESI): found: *m*/*z* = 384.1 [M + H^+^], calculated: *m*/*z* = 384.2 [M + H^+^].

#### *N*-(3-((2-((9*H*-Carbazol-3-yl)oxy)ethyl)amino)-3-oxopropyl)-4-aminobutanamide Hydrochloride (**8d**)

Synthesis was performed according to general method C. **8c** (320 mg, 663 μmol, 1.00 equiv) was used. Solvent was concentrated under reduced pressure and the residue was lyophilized in water. The target compound **8d** (275 mg, 656 μmol, quantitative) was isolated as colorless solid. Mp: 240–243 °C. ^1^H NMR (300 MHz, DMSO-*d*_6_): δ = 11.13 (s, 1H), 8.26 (s, 1H), 8.20–7.97 (m, 5H), 7.70 (d, *J* = 2.4 Hz, 1H), 7.49–7.26 (m, 3H), 7.17–6.97 (m, 2H), 4.07 (t, *J* = 5.8 Hz, 2H), 3.48 (q, *J* = 6.0 Hz, 2H), 3.28 (q, *J* = 6.7 Hz, 2H), 2.85–2.64 (m, 2H), 2.32 (t, *J* = 7.1 Hz, 2H), 2.18 (t, *J* = 7.2 Hz, 2H), 1.79 (p, *J* = 7.4 Hz, 2H). ^13^C NMR (75 MHz, DMSO-*d*_6_): δ = 171.3, 170.8, 152.0, 140.4, 134.7, 125.4, 122.8, 122.4, 120.3, 118.0, 115.3, 111.6, 111.0, 104.3, 67.2, 38.4, 35.4, 35.4, 32.1, 23.2. MS (ESI): found: *m*/*z* = 383.1 [M + H^+^], calculated: *m*/*z* = 383.2 [M + H^+^].

Synthesis of **7** and **8** was performed according to Pirali et al.^114^ reaction conditions. Tetrahydrofuran-BH_3_ complex (1.00 M in THF, 20.0 equiv) was added dropwise to a solution of amide **7d** or **8d** (50.0 mg, 1.00 equiv) in dry THF (0.5 mL) under ice cooling and inert gas atmosphere. After addition, the mixture was let to reach room temperature and was stirred for 3 d at 60 °C. The reaction mixture was quenched with methanol (gas evolution) and the solvent was evaporated. The resulting borane-amine complex was dissolved differently.

#### *N*^1^-(3-((2-(Dibenzo[b,d]furan-2-yloxy)ethyl)amino)propyl)butane-1,4-diamine Trifluoroacetate Salt (**7**)

The mixture was taken in 2.4 mL water. 0.6 mL TFA was added (in total 20 vol %) and the mixture stirred for 30 min to destroy the borane-amine complex. The solution was lyophilized and the crude product was purified via reversed phase flash chromatography (acetonitrile:water 0.1% TFA; gradient 0–25% acetonitrile). The product was further purified via preparative HPLC (gradient 10–100% acetonitrile in water + 0.1% TFA, eluates at 30%). The molecular weight was determined according to the described general method A. The target compound **7** (874.6 g/mol (∼5 xTFA), 54.8 mg, 0.063 mmol, 53%) was isolated as colorless solid. Mp: 233–236 °C. ^1^H NMR (300 MHz, CD_3_OD): δ = 8.01 (dd, *J* = 7.7, 1.3 Hz, 1H), 7.67 (d, *J* = 2.6 Hz, 1H), 7.59–7.44 (m, 3H), 7.36 (td, *J* = 7.4, 1.2 Hz, 1H), 7.19 (dd, *J* = 8.9, 2.6 Hz, 1H), 4.42 (t, *J* = 5.1 Hz, 2H), 3.56 (t, *J* = 4.9 Hz, 2H), 3.36–3.25 (m, 2H), 3.25–3.15 (m, 2H), 3.11 (t, *J* = 7.4 Hz, 2H), 3.00 (t, *J* = 7.1 Hz, 2H), 2.33–2.15 (m, 2H), 1.91–1.70 (m, 4H). ^13^C NMR (75 MHz, CD_3_OD): δ = 158.4, 155.6, 152.8, 128.6, 126.1, 125.5, 123.8, 121.8, 117.0, 113.2, 112.6, 106.3, 65.3, 48.4, 48.3, 46.0, 45.8, 40.0, 25.6, 24.2, 24.0. MS (ESI): found: *m*/*z* = 356.0 [M + H^+^], calculated: *m*/*z* = 356.2 [M + H^+^]. 98% purity.

#### N^1^-(3-((2-((9*H*-Carbazol-3-yl)oxy)ethyl)amino)propyl)butane-1,4-diamine Trifluoroacetate Salt (**8**)

The mixture was taken in 2.4 mL water. 0.6 mL TFA were added (in total 20 vol %) and the mixture was stirred in total for 6 h to destroy the amine-borane complexes, which was not completed. The mixture was lyophilized and taken in 2.2 mL water and 0.4 mL 10% sodium hydroxide solution. Stirring for 30 min resulted in gas evolution and completely dissolved amine-borane complexes. The mixture was lyophilized and the crude product was purified via flash chromatography reversed phase (acetonitrile:water 0.1% TFA; gradient 0–25% acetonitrile). Crude product was further purified via HPLC (gradient 5–100% acetonitrile in water + 0.1% TFA, eluates at 10–20%). The molecular weight was determined according to the described general method A. The target compound **8** (827.2 g/mol (ca. 5× TFA), 41.0 mg, 0.050 mmol, 42% yield) was isolated as colorless solid (pale brown in solution). Mp: 211–213 °C. ^**1**^**H NMR** (300 MHz, DMSO-*d*_6_): δ = 11.18 (s, 1H), 8.07 (d, *J* = 7.8 Hz, 1H), 7.74 (d, *J* = 2.5 Hz, 1H), 7.52–7.29 (m, 3H), 7.18–7.05 (m, 2H), 4.34 (t, *J* = 4.9 Hz, 2H), 3.60–3.30 (m, 2H), 3.15 (t, *J* = 7.7 Hz, 2H), 3.04 (t, *J* = 7.7 Hz, 2H), 2.94 (t, *J* = 7.2 Hz, 2H), 2.82 (t, *J* = 7.0 Hz, 2H), 2.15–1.96 (m, 2H), 1.63 (m, 4H). ^**13**^**C NMR** (75 MHz, DMSO-*d*_6_): δ = 151.4, 140.5, 135.1, 125.6, 122.8, 122.4, 120.2, 118.1, 115.4, 111.7, 111.1, 104.7, 64.4, 46.3, 46.1, 44.4, 43.9, 38.2, 24.1, 22.6, 22.3. **MS (ESI):** found: *m*/*z* = 355.0 [M+H^+^], calculated: *m*/*z* = 355.3 [M+H^+^]. 97% purity.

#### RNA Oligonucleotides

RNA oligonucleotides except for the label-free *Ba* SAM-VI riboswitch which was prepared by polymerase chain reaction (PCR, see below) and in vitro transcription (IVT, see below), were purchased from Biomers.net GmbH (Ulm, Germany) (5′-biotin-*Ba* SAM-VI riboswitch, 5′-biotin-*Tte* preQ_1_ riboswitch, *Tte* preQ_1_ riboswitch) and Integrated DNA Technologies (Coralville, IA, USA) (5′-Cy5-*Tte* preQ_1_ riboswitch, 5′-Cy5-*Ba* SAM-VI riboswitch) in HPLC-purified quality (for oligonucleotide sequences see Tables S7 and S8). The concentrations of RNA were determined using a NanoDrop2000 (ThermoFisher Scientific, Waltham, MA, USA). All experiments were performed under RNase-avoiding conditions. All experiments including Cy5- and biotin-labeled RNA were performed under light protection.

#### Polymerase Chain Reaction

The DNA template for the IVT was amplified using PCR. The PCR reaction mixture contained 10 nM of template DNA, 2 μM of each forward and reverse primer, 400 μM dNTP Mix (New England Biolabs GmbH, Frankfurt, Germany)), 3 mM MgCl_2_, 0.05 U mL^–1^*Taq* DNA Polymerase (New England Biolabs GmbH, Frankfurt, Germany) and 1× standard *Taq* reaction buffer (New England Biolabs GmbH, Frankfurt, Germany) in a final volume of 200 μL. The reaction was performed using a ^3^Prime thermal cycler (Cole-Parmer Ltd., Stone, St, U.K.) with the following cycle parameters: initial denaturation (95 °C, 30 s), followed by 30 cycles of denaturation (95 °C, 30 s), annealing (temperature depends on the primer sequence and was determined by NEB Tm calculator, 30 s) and extension (72 °C, 45 s), and a final extension (72 °C, 4 min). The PCR product homogeneity was determined on a 10% native PAGE gel to confirm the size and purity.

#### In Vitro Transcription

The crude PCR products were used as templates for IVT using the T7 polymerase system. The IVT reaction mixture contained 400 μL PCR product, (without further purification), 7.5 mM of each NTP (Jena Bioscience, Jena, Germany), 10 mM DTT, 30 mM MgCl_2_, 5 μL HighYield T7 RNA polymerase mix (Jena Bioscience, Jena, Germany) and 1× HighYield T7 reaction buffer (Jena Bioscience) in a final volume of 1 mL. The reaction was incubated at 37 °C for 4 h. The formed pyrophosphate was removed by centrifugation at 14,000 g for 5 min directly after transcription and the cleared supernatant was transferred to a micro reaction tube for further sample processing. To remove the PCR- and IVT-enzymes, phenol/chloroform extraction was performed twice using ROTI phenol/chloroform/isoamyl alcohol (Carl Roth, Karlsruhe, Germany) and the standard protocol from the supplier. For further purification and concentration of the RNA, the RNA Clean & Concentrator-5 kit (Zymo Research Europe GmbH, Freiburg, Germany) including DNase I was used. The RNA product was determined on a 10% denaturing PAGE gel to confirm size and purity.

#### Surface Plasmon Resonance Spectroscopy (SPR)

SPR assays were performed in a Biacore T200 using carboxymethyl dextran sensor chips precoated with streptavidin (SA Sensor Chip Series S). All experiments were carried out at a constant temperature of 25 °C in buffer (50 mM HEPES pH 7.0, 150 mM NaCl, 6 mM MgCl_2_) as running buffer. Before immobilizing the RNA, the chips were equilibrated by three injections of 1 M NaCl/50 mM NaOH applied at a flow rate of 10 μL/min. Biotinylated RNA samples were heated to 75 °C for 5 min and cooled down to room temperature for 20 min prior immobilization. Then, the respective biotinylated RNA riboswitch aptamer (200 nM) was injected at a flow rate of 10 μL/min for a total contact time of 300 s. Approximately 2500–3000 RU of the relevant RNA aptamer was bound per flow cell. Analyses of the kinetics of interaction of the respective riboswitch (*Ba* SAM-VI, *Tte* preQ_1_, and *Bs* preQ_1_ riboswitch with the different ligands were performed at a flow rate of 30 μL/min in running buffer at 25 °C as single cycle kinetic. Various concentrations of the metabolites (preQ_1_: 5, 50, 500, 5000, 50,000 nM; SAH and **5**: 1953, 7812, 31,250, 125,000, 500,000 nM; **3**: 50, 500, 5000, 50,000, 500,000 nM; **4** and **8**: 390, 1560, 6250, 25,000, 100,000 nM; **7**: 0.5, 5, 50, 500, 5000 nM; SAM: 123, 370, 1111, 3333, 10,000 nM; **1** and **2**: 1000, 6250, 12,500, 25,000, 50,000 nM; preQ_0_: 188, 375, 750, 1500, 3000 nM; and **6:** 938, 1875, 3750, 7500, 15,000 nM), dissolved in running buffer or running buffer containing 2% DMSO (for analytes preQ_0_ and **6**) were passed over the flow cells for 240 or 180 s (samples containing DMSO), and the complexes formed were allowed to dissociate for 600 s before the next cycle started. After each cycle, the surface was regenerated by injection of 3 M guanidine hydrochloride for 60 s, followed by 1 M NaCl for 60 s, at a flow rate of 30 μL/min. All experiments were performed at 25 °C. Sensorgrams were recorded using Biacore T200 Control Software 3.2 and analyzed with Biacore T200 Evaluation Software 3.2.1. The surface of flow cell 1 was not coated with RNA and used to obtain blank sensorgrams for subtraction of the bulk refractive index background. The referenced sensorgrams were normalized to a baseline of 0. Peaks in the sensorgrams at the beginning and the end of the injection are due to the run-time difference between the flow cells for each chip.

#### Interaction Map (IM) Analysis

IM calculations were performed on the Ridgeview Diagnostic Server (Ridgeview Diagnostics, Uppsala, Sweden). For this purpose, the SPR sensorgrams were exported from the Biacore T200 Evaluation Software 3.2.1 as *.txt files and imported into TraceDrawer Software 1.10.1 (Ridgeview Instruments, Uppsala, Sweden). IM files were created using the IM tool within the software, generating files that were sent via e-mail to the server (im@ridgeviewdiagnostics.com), where the IM calculations were performed.^92^ The resulting files were then evaluated for spots in the TraceDrawer 1.10.1 Software, and the IM spots were quantified.

#### Isothermal Titration Calorimetry

ITC experiments were performed on an MicroCal PEAQ-ITC Automated calorimeter (Malvern Panalytical, Malvern, U.K.) at 25 °C. Prior to titration, the RNA was desalted using Zebra spin desalting columns (ThermoFisher Scientific, Waltham, MA, USA) following the standard protocol from the supplier, and lyophilized. RNA was resolved in ITC buffer (50 mM HEPES pH 7.0, 100 mM NaCl, 6 mM MgCl_2_ for *Tte* preQ_1_ riboswitch and 40 mM HEPES pH 7.0, 50 mM KCl, 10 mM MgCl_2_ for *Ba* SAM-VI riboswitch). For refolding, the RNA was heated to 75 °C for 5 min and cooled down to room temperature for 20 min. For the *Tte* preQ_1_ riboswitch experiments, the RNA was diluted to 20 μM in ITC buffer, preQ_1_ and preQ_0_ were diluted to 200 μM in ITC buffer. For each experiment, 19 ligand injections in a volume of 2.0 μL, with a 0.5 μL s^–1^ rate, 150 s intervals between injections and a reference power of 41.9 μW were performed. For the *Ba* SAM-VI riboswitch experiments, the RNA was diluted to 15 μM (SAM, **1** and **2** measurements) and 30 μM (SAH measurements) in ITC buffer. SAM, **1** and **2** were diluted to 150 μM in ITC buffer, SAH was diluted to 300 μM in ITC buffer. For each experiment, 13 ligand injections in a volume of 3.0 μL, with a 0.5 μL s^–1^ rate, 150 s intervals between injections and a reference power of 41.9 μW were performed. All experiments contained a final DMSO concentration of 0.2%. Due to high sample consumption, thermodynamic profiles are not corrected for buffer ionization effects.^79,115^ Integrated heat data were analyzed using a one-site binding model via the MicroCal PEAQ-ITC Analysis Software (version 1.21), provided by the manufacturer. Kinetic data were analyzed using the AFFINImeter web tool (Software 4 Science Developments, https://www.affinimeter.com). All ITC experiments were independently repeated three times.

#### Microscale Thermophoresis

MST experiments were performed on a Monolith NT.115 (NanoTemper Technologies, Munich, Germany) using standard uncoated capillaries as described previously.^116−118^ Briefly, for refolding, the 5′-Cy5-labeled RNA was heated to 75 °C for 5 min in MST buffer (50 mM Tris–HCl, pH 7.5, 100 mM KCl, 25 mM MgCl_2_) and cooled down to room temperature for 20 min. The RNA was diluted to 20 nM in MST buffer and supplemented with ligands in appropriate concentrations ranging from 1 mM to 0.1 nM in 3.16-fold (semilogarithmic) dilution series. Cross-selectivity experiments were performed under the same conditions, but with ligand concentrations of 1 mM, 100 μM, 1 μM, 100 nM and 1 nM. For each experiment, one DMSO negative control was carried out. Final DMSO concentration was always 2%. MST measurements (read at 5 s laser on-time) were analyzed using the NT analysis software (version 1.5.41) and exported for statistical analysis and plotting in GraphPad Prism (version 8.0.1, GraphPad Software, Boston, MA, USA, https://www.graphpad.com).

## Supporting Information Available

Data of SPR measurements and representative sensorgrams; data of MST measurements: fluorescence-time traces, dose–response curves; data of (kin)ITC experiments, ITC thermograms and isotherms, signature plots, and ETCs; NMR spectra, mass spectra, and LC-MS chromatograms of all compounds tested in assays; and miscellaneous: native PAGE of *Tte* preQ_1_ riboswitch, crystal structures, and RNA sequences (PDF)
Molecular formula strings (CSV)

## Abbreviations

AUC: area under curve
ASO: antisense oligonucleotide
*Ba*: *Bifidobacterium angulatum*
Boc: *tert*-butoxycarbonyl
Boc_2_O: di-*tert*-butyl decarbonate
Cy5: Cyanine5
DCM: methylene chloride
DIPEA: *N,N*-di*iso*propylethylamine
DM2: myotonic dystrophy type 2
DMF: dimethylformamide
DMSO: dimethyl sulfoxide
DNase: deoxyribonuclease
dNTP: deoxynucleotide triphosphate
ETC: equilibration time curve
ESI: electron spray ionization
HCV IRES: hepatitis C virus internal ribosome entry site
HEPES: (4-(2-hydroxyethyl)-1-piperazineethanesulfonic acid)
HPLC: high-performance liquid chromatography
mp: melting point
IM: Interaction map
IVT: in vitro transcription
ITC: isothermal titration calorimetry
MS: mass spectrometry
MST: microscale thermophoresis
messenger RNA: mRNA
NMR: nuclear magnetic resonance
PAGE: polyacrylamide gel electrophoresis
PCR: polymerase chain reaction
PINAD: proximity-induced nucleic acid degrader
preQ_1_: prequeuosine-1
preQ_0_: prequeuosine-0
PROTAC: proteolysis targeting chimera
RiboTAC: ribonuclease targeting chimera
RNase: ribonuclease
RU: response units
SAH: *S*-adenosyl homocysteine
SAM: *S*-adenosyl methionine
siRNA: small interfering RNA
SMA: spinal muscular atrophy
SMN2: survival motor neuron 2
SPR: surface plasmon resonance spectroscopy
*Taq*: *Thermus aquaticus*
TAR: trans-activation response element
TBTU: benzotriazol-1-yloxy(dimethylamino)methylidene]-dimethylazanium tetrafluoroborate
TFA: trifluoroacetic acid
THF: tetrahydrofuran
TLC: thin layer chromatography
Tm: melting temperature
TPP: thiamine pyrophosphate
Tris-HCl: Tris(hydroxymethyl)aminomethane hydrochloride
*Tte*: *Thermoanaerobacter tengcongensis*.
