# Supplementary material for: Electrostatic Anchoring in RNA-Ligand Design—Dissecting the Effects of Positive Charges on Affinity, Selectivity, Binding Kinetics, and Thermodynamics
Source: J Med Chem. 2025 Apr 7;68(8):8659–78. doi: 10.1021/acs.jmedchem.5c00339 (PMC12035807; doi:10.1021/acs.jmedchem.5c00339)
Supplement: Supplementary file 1 — jm5c00339_si_001.pdf [file jm5c00339_si_001.pdf]

Supporting Information

Electrostatic Anchoring in RNA-Ligand Design – Dissecting the Effects of positive Charges on Affinity, Selectivity, Binding Kinetics and Thermodynamics

Laura Almena Rodriguez[1,‡], Elisabeth Kallert[1,‡], Jan-Åke Husmann[1], Kirsten Schaubruch[2], Katherina I. S. Meisel[1], Marvin Schwickert[1], Sabrina N. Hoba[1], Ralf Heermann[2], Christian Kersten[1,3,*]

[1] Institute of Pharmaceutical and Biomedical Sciences, Johannes Gutenberg-University, Staudingerweg 5, 55128 Mainz, Germany.

[2] Institute of Molecular Physiology, Microbiology and Biotechnology, Johannes Gutenberg-University, Hanns-DieterHüsch-Weg 17, 55128 Mainz, Germany

[3] Institute for Quantitative and Computational Biosciences, Johannes Gutenberg-University, BioZentrum I, Hanns-Dieter-Hüsch Weg 15, 55128 Mainz, Germany

‡ LAR & EK contributed equally to this manuscript

*correspondence: kerstec@uni-mainz.de

Table of Contents

1. SPR Experiments (Figures S1-S3, Tables S1, S2) 2
2. MST Experiments (Figures S4-S11) 8
3. ITC Experiments (Figures S12-S17, Tables S3-S6) 10
4. Analytical data compounds 3–8 (Figures S18-S23) 17
5. Miscellaneous (Figures S24–S26, Tables S7, S8) 23

1. SPR Experiments (Figures S1-S3, Tables S1, S2)

Table S1. Binding affinities, kinetic parameters and fit qualities (chi^2) from SPR measurements for SAM, SAH, **1**, and **2** against the *Ba* SAM–VI riboswitch, 1:1 binding fit model.

Ba SAM-VI	SPR				
**Compound**	**K_D [µM]**	**k_{on} [1/Ms]**	**k_{off} [1/s]**	**chi^2**	**$K_{D,SS}$ [µM]**
**SAM**	3.02	$2.51 \cdot 10^3$	$7.59 \cdot 10^{-3}$	0.15	5.27
	3.74	$4.76 \cdot 10^3$	$1.78 \cdot 10^{-2}$	0.04	4.83
	3.51	$2.52 \cdot 10^3$	$8.85 \cdot 10^{-3}$	0.07	5.17
	3.63	$2.38 \cdot 10^3$	$8.64 \cdot 10^{-3}$	0.18	4.60
	3.47	$2.44 \cdot 10^3$	$8.46 \cdot 10^{-3}$	0.21	4.68
	3.78	$3.72 \cdot 10^3$	$1.40 \cdot 10^{-2}$	0.15	5.15
	3.36	$2.61 \cdot 10^3$	$8.78 \cdot 10^{-3}$	0.18	4.67
	3.21	$2.60 \cdot 10^3$	$8.34 \cdot 10^{-3}$	0.23	4.85
	2.39	$2.90 \cdot 10^3$	$6.93 \cdot 10^{-3}$	0.18	3.40
	2.96	$3.16 \cdot 10^3$	$9.36 \cdot 10^{-3}$	0.04	3.54
	2.86	$3.14 \cdot 10^3$	$8.98 \cdot 10^{-3}$	0.05	3.69
**SAM Average**	**3.72±0.41**	**$(2.98±0.68) \cdot 10^3$**	**$(9.80±3.06) \cdot 10^{-3}$**		**4.53±0.64**
**SAH**	64.9	$6.95 \cdot 10^2$	$4.51 \cdot 10^{-2}$	0.04	58.3
	172	$2.86 \cdot 10^2$	$4.92 \cdot 10^{-2}$	0.34	102
	164	$2.93 \cdot 10^2$	$4.82 \cdot 10^{-2}$	0.20	105
	167	$2.90 \cdot 10^2$	$4.85 \cdot 10^{-2}$	0.23	127
	79.0	$5.38 \cdot 10^2$	$4.25 \cdot 10^{-2}$	0.04	80.0
	128	$4.74 \cdot 10^2$	$6.04 \cdot 10^{-2}$	0.45	89.2
	192	$3.29 \cdot 10^2$	$6.31 \cdot 10^{-2}$	1.07	141
	189	$3.16 \cdot 10^2$	$5.97 \cdot 10^{-2}$	0.83	157
	171	$3.35 \cdot 10^2$	$5.73 \cdot 10^{-2}$	0.61	160
	168	$3.70 \cdot 10^2$	$6.21 \cdot 10^{-2}$	1.11	140
	149	$3.75 \cdot 10^2$	$5.60 \cdot 10^{-2}$	0.60	137
	156	$3.61 \cdot 10^2$	$5.61 \cdot 10^{-2}$	0.63	154
**SAH Average**	**150±39**	**$(3.88±1.18) \cdot 10^2$**	**$(5.40±0.67) \cdot 10^{-2}$**		**121±32**
**1**	14.2	$2.72 \cdot 10^3$	$3.88 \cdot 10^{-2}$	0.16	24.4
	14.0	$2.73 \cdot 10^3$	$3.83 \cdot 10^{-2}$	0.20	25.3
	14.2	$2.77 \cdot 10^3$	$3.93 \cdot 10^{-2}$	0.31	26.0
	11.8	$2.95 \cdot 10^3$	$3.48 \cdot 10^{-2}$	0.21	17.6
	11.8	$3.00 \cdot 10^3$	$3.54 \cdot 10^{-2}$	0.18	18.5
	11.6	$3.05 \cdot 10^3$	$3.53 \cdot 10^{-2}$	0.16	20.0
	11.3	$3.05 \cdot 10^3$	$3.44 \cdot 10^{-2}$	0.19	18.4
	11.0	$3.12 \cdot 10^3$	$3.41 \cdot 10^{-2}$	0.18	18.2
	11.2	$3.11 \cdot 10^3$	$3.47 \cdot 10^{-2}$	0.17	18.3
**1 Average**	**12.3±1.3**	**$(2.94±0.15) \cdot 10^3$**	**$(3.61±0.19) \cdot 10^{-2}$**		**20.7±3.3**
**2**	6.88	$7.16 \cdot 10^3$	$4.93 \cdot 10^{-2}$	0.30	37.8
	5.70	$8.64 \cdot 10^3$	$4.93 \cdot 10^{-2}$	0.30	33.8
	6.40	$8.46 \cdot 10^3$	$5.41 \cdot 10^{-2}$	0.34	34.7
	3.09	$1.49 \cdot 10^4$	$4.59 \cdot 10^{-2}$	0.26	6.02
	3.10	$1.48 \cdot 10^4$	$4.59 \cdot 10^{-2}$	0.23	6.05
	3.28	$1.48 \cdot 10^4$	$4.85 \cdot 10^{-2}$	0.32	5.99
	3.00	$1.50 \cdot 10^4$	$4.49 \cdot 10^{-2}$	0.28	6.15
	2.96	$1.55 \cdot 10^4$	$4.60 \cdot 10^{-2}$	0.22	6.16
	3.08	$1.58 \cdot 10^4$	$4.87 \cdot 10^{-2}$	0.26	6.29
**2 Average**	**4.17±1.56**	**$(1.28±0.34) \cdot 10^4$**	**$(4.81±0.27) \cdot 10^{-2}$**		**15.9±13.8**

Table S2. Binding affinities, kinetic parameters and fit qualities (chi^2) from SPR measurements for $preQ_0$ and $preQ_1$ against the *Tte* $preQ_1$ riboswitch, 1:1 binding fit model. [a] The 1:1 binding model underlies the steady-state analysis, thus not representing the best fitting model for preQ1 and preQ0.

Tte $\mathbf{preQ_1}$	**SPR**				
**Compound**	K_D **[nM]**	k_{on} **[1/Ms]**	k_{off} **[1/s]**	chi^2	$K_{D,SS}$ **[nM]**[a]
$\mathbf{preQ_0}$	64.3	$3.18 \cdot 10^3$	$2.05 \cdot 10^{-4}$	1.19	633
	107	$3.13 \cdot 10^3$	$3.35 \cdot 10^{-4}$	1.10	769
	107	$3.11 \cdot 10^3$	$3.32 \cdot 10^{-4}$	1.06	761
	88.3	$3.17 \cdot 10^3$	$2.80 \cdot 10^{-4}$	0.27	939
	95.2	$3.81 \cdot 10^3$	$3.63 \cdot 10^{-4}$	0.29	754
	89.8	$3.80 \cdot 10^3$	$3.41 \cdot 10^{-4}$	0.30	783
	53.2	$4.26 \cdot 10^3$	$2.26 \cdot 10^{-4}$	0.42	641
	85.5	$3.99 \cdot 10^3$	$3.41 \cdot 10^{-4}$	0.21	829
	83.1	$4.06 \cdot 10^3$	$3.38 \cdot 10^{-4}$	0.20	839
	21.7	$1.45 \cdot 10^4$	$3.14 \cdot 10^{-4}$	0.35	313
	19.4	$1.59 \cdot 10^4$	$3.08 \cdot 10^{-4}$	0.39	300
$\mathbf{preQ_0}$ **Average**	**74.0±29.4**	$\mathbf{(5.72 \pm 4.49) \cdot 10^3}$	$\mathbf{(3.07 \pm 0.48) \cdot 10^{-4}}$		**687±197**
$\mathbf{preQ_1}$	26.6	$1.04 \cdot 10^4$	$2.77 \cdot 10^{-4}$	16.26	389
	19.1	$1.05 \cdot 10^4$	$2.00 \cdot 10^{-4}$	14.15	410
	18.5	$1.03 \cdot 10^4$	$1.90 \cdot 10^{-4}$	11.44	431
	26.3	$9.60 \cdot 10^3$	$2.52 \cdot 10^{-4}$	9.57	474
	21.6	$1.00 \cdot 10^4$	$2.17 \cdot 10^{-4}$	8.58	446
	23.0	$9.65 \cdot 10^3$	$2.22 \cdot 10^{-4}$	7.80	528
	44.0	$9.81 \cdot 10^3$	$4.32 \cdot 10^{-4}$	3.07	396
	47.4	$9.99 \cdot 10^3$	$4.74 \cdot 10^{-4}$	2.27	455
	40.1	$1.00 \cdot 10^4$	$4.03 \cdot 10^{-4}$	2.23	482
	35.8	$1.15 \cdot 10^4$	$4.10 \cdot 10^{-4}$	1.28	309
	32.8	$1.20 \cdot 10^4$	$3.94 \cdot 10^{-4}$	1.12	297
	34.9	$1.17 \cdot 10^4$	$4.07 \cdot 10^{-4}$	0.93	329
	26.1	$1.12 \cdot 10^4$	$2.91 \cdot 10^{-4}$	6.13	329
	19.5	$1.19 \cdot 10^4$	$2.32 \cdot 10^{-4}$	7.32	300
	19.1	$1.18 \cdot 10^4$	$2.27 \cdot 10^{-4}$	7.18	327
	21.4	$1.02 \cdot 10^4$	$2.18 \cdot 10^{-4}$	15.61	736
	14.1	$1.19 \cdot 10^4$	$1.67 \cdot 10^{-4}$	15.25	712
	14.1	$1.19 \cdot 10^4$	$1.68 \cdot 10^{-4}$	13.62	768
	23.5	$9.61 \cdot 10^3$	$2.25 \cdot 10^{-4}$	11.91	838
	19.2	$1.14 \cdot 10^4$	$2.19 \cdot 10^{-4}$	9.33	777
	24.7	$1.01 \cdot 10^4$	$2.49 \cdot 10^{-4}$	7.60	914
	28.4	$1.11 \cdot 10^4$	$3.14 \cdot 10^{-4}$	7.88	265
	15.7	$1.23 \cdot 10^4$	$1.94 \cdot 10^{-4}$	7.52	254
	15.3	$1.20 \cdot 10^4$	$1.83 \cdot 10^{-4}$	5.52	284
	42.5	$9.29 \cdot 10^3$	$3.94 \cdot 10^{-4}$	12.59	593
	10.6	$1.12 \cdot 10^4$	$1.19 \cdot 10^{-4}$	10.92	579
	11.0	$1.09 \cdot 10^4$	$1.20 \cdot 10^{-4}$	8.88	668
	19.5	$1.05 \cdot 10^4$	$2.04 \cdot 10^{-4}$	6.42	607
	14.4	$1.32 \cdot 10^4$	$1.90 \cdot 10^{-4}$	5.82	572
	15.3	$1.31 \cdot 10^4$	$2.00 \cdot 10^{-4}$	4.76	619
$\mathbf{preQ_1}$ **Average**	**24.1±9.9**	$\mathbf{(1.10 \pm 0.11) \cdot 10^4}$	$\mathbf{(2.60 \pm 0.96) \cdot 10^{-4}}$		**503±184**

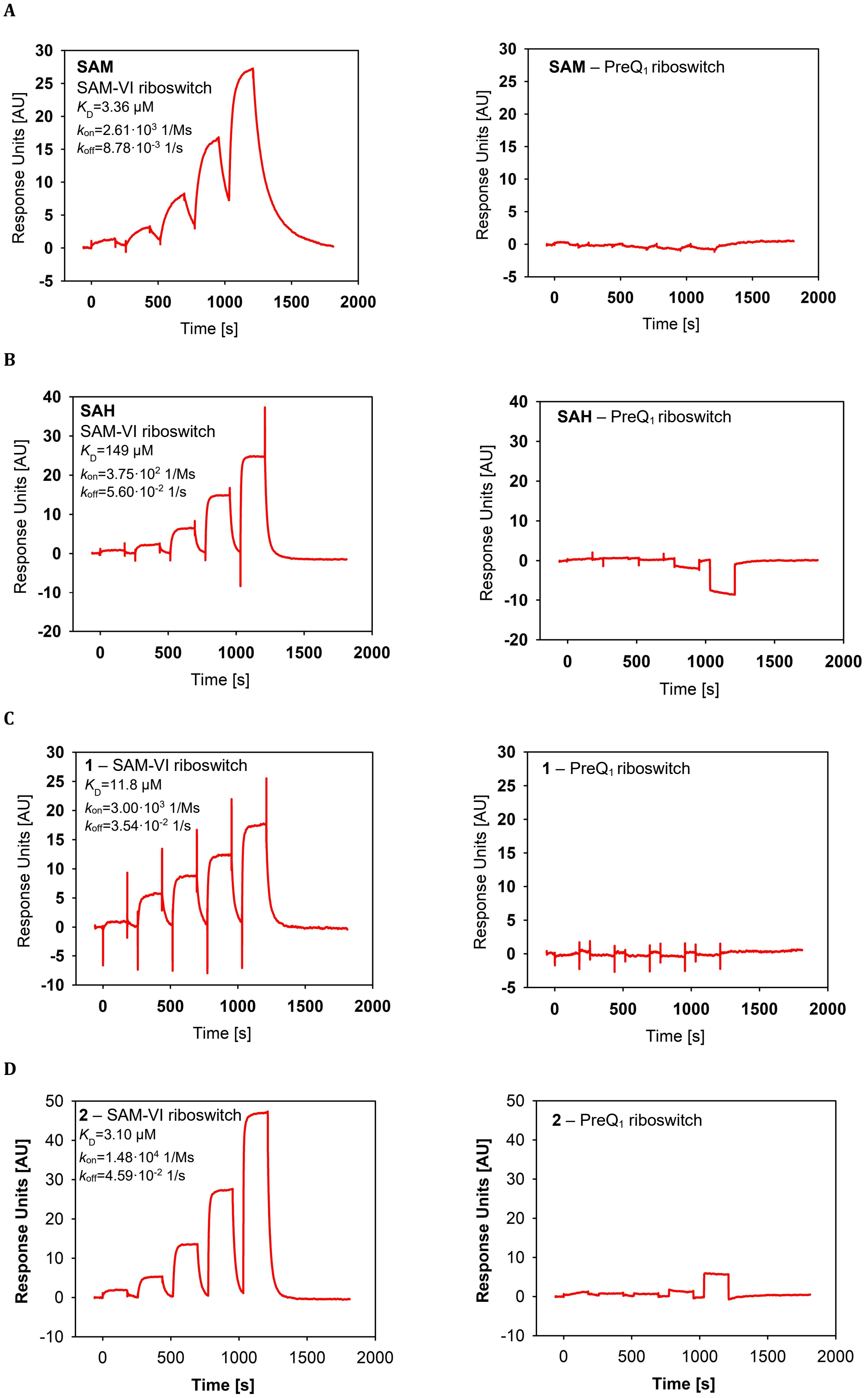

Figure S1. Representative SPR sensorgrams for SAM (**A**), SAH (**B**), **1** (**C**) and **2** (**D**) binding to the *Ba* SAM-VI riboswitch (left panel) and binding to the *Tte* preQ1 riboswitch (right panel) 1:1 binding mode fit.

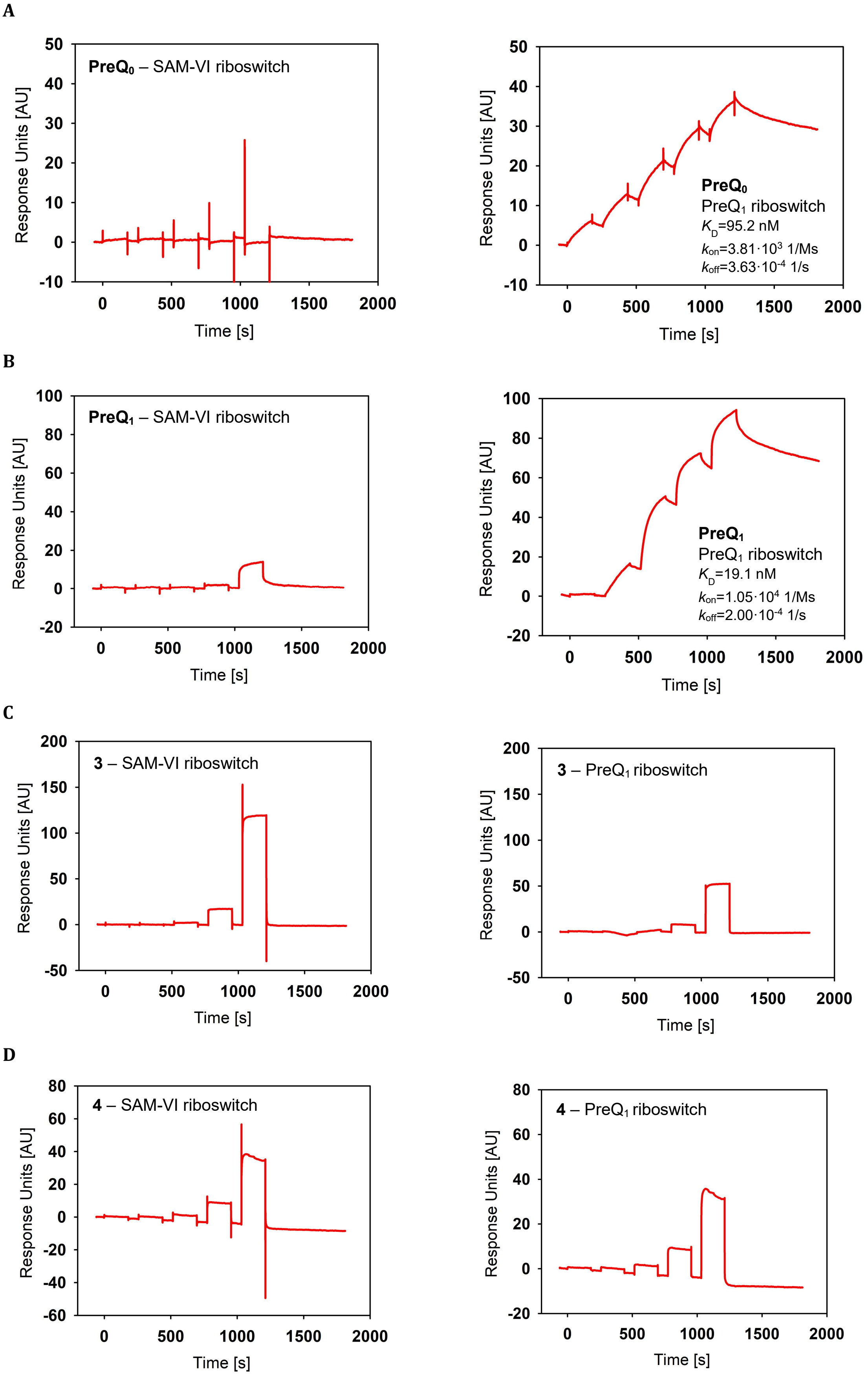

Figure S 2. Representative SPR sensorgrams for preQ$_0$ (**A**), preQ$_1$ (**B**), **3** (**C**), **4** (**D**), **5** (**E**), **6** (**F**), **7** (**G**) and **8** (**H**) binding to the *Ba* SAM-VI riboswitch (left panel) and binding to the *Tte* preQ$_1$ riboswitch (right panel). 1:1 binding mode fit. Compounds **3–8** show fast on/fast off, loose binding behavior to both targets.

E

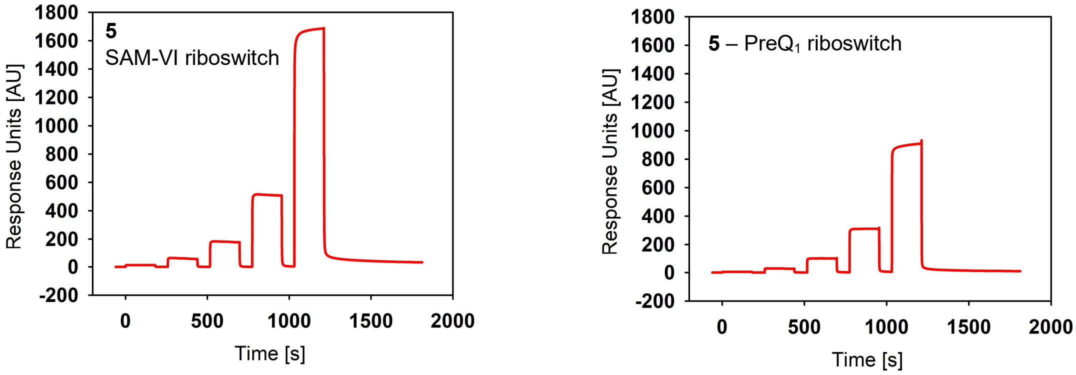

F

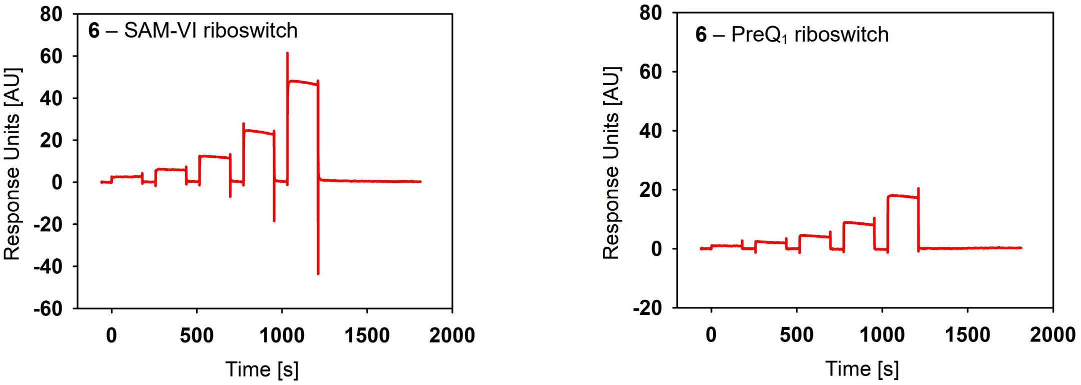

G

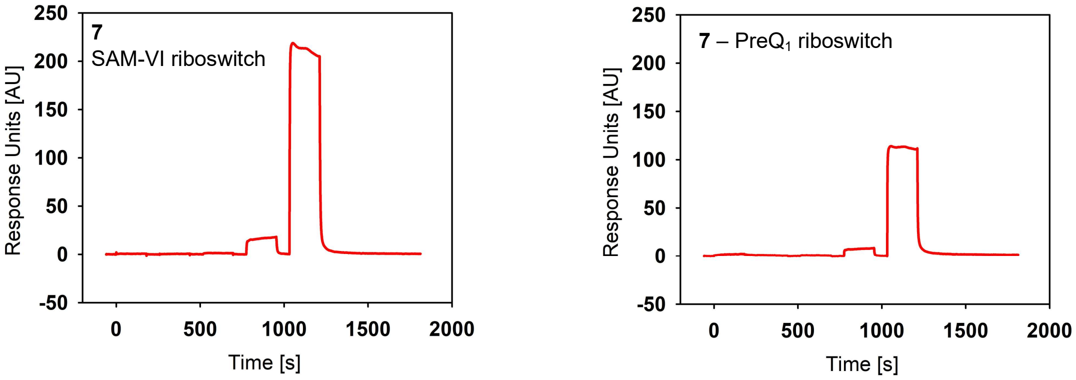

H

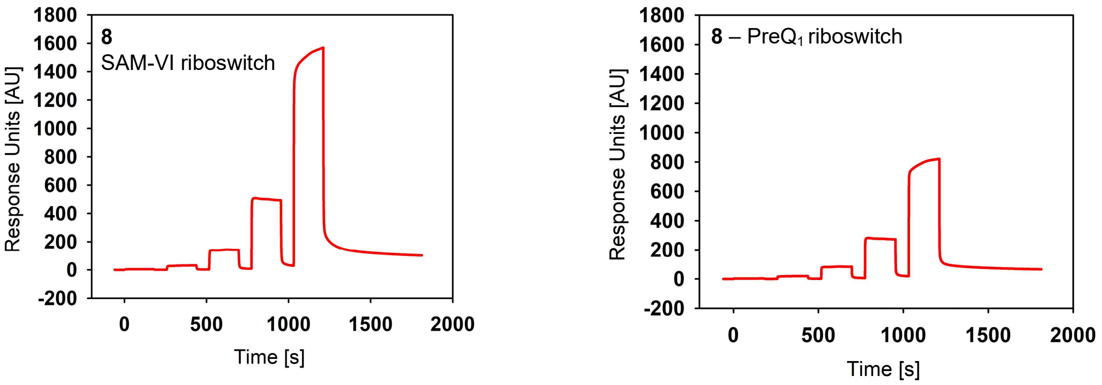

Figure S 2 continued. Representative SPR sensorgrams for preQ$_0$ (**A**), preQ$_1$ (**B**), **3** (**C**), **4** (**D**), **5** (**E**), **6** (**F**), **7** (**G**) and **8** (**H**) binding to the *Ba* SAM-VI riboswitch (left panel) and binding to the *Tte* preQ$_1$ riboswitch (right panel). 1:1 binding mode fit. Compounds **3–8** show fast on/fast off, loose binding behavior to both targets.

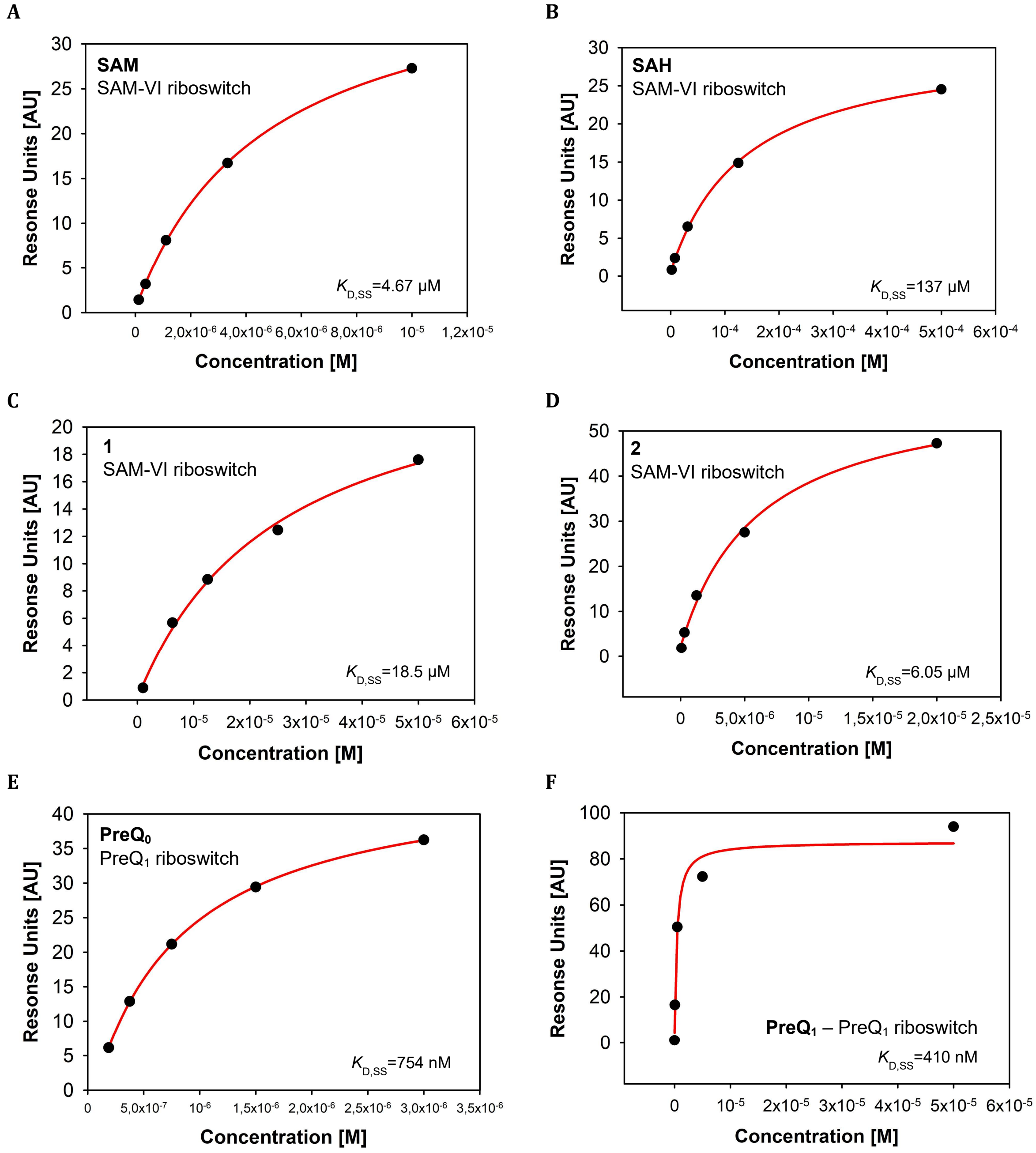

Figure S 3. Representative SPR affinity plots of steady-state analysis for SAM (**A,** corresponding to SPR sensorgram Figure **S1 A**), SAH (**B**, corresponding to SPR sensorgram Figure **S1 B**), 1 (**C**, corresponding to SPR sensorgram Figure **S1 C**), 2 (**D**, corresponding to SPR sensorgram Figure **S1 D**), preQ$_0$ (**E**, corresponding to SPR sensorgram Figure **S2 A**) and preQ$_1$ (**F**, corresponding to SPR sensorgram Figure **S2 B**) binding to their target Ba SAM-VI riboswitch or to the *Tte* preQ$_1$ riboswitch, respectively.

2. MST Experiments (Figures S4-S11)

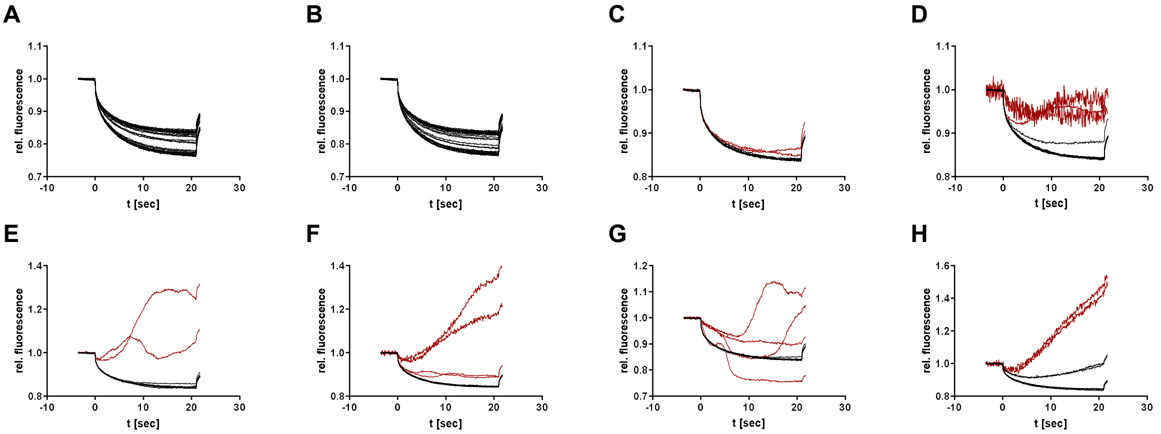

Figure S4. MST results at concentrations 0.1 nM, 0.316 nM, 1 nM, 3.16 nM, 10 nM, 31.6 nM, 100 nM, 316 nM, 1 µM, 3.16 µM, 10 µM for preQ$_1$ (**A**) and preQ$_0$ (**B**) binding to the *Tte* preQ$_1$ riboswitch. MST results at concentrations of 1 nM, 0.1 µM, 1 µM, 100 µM, 1 mM for **3** (**C**), **4** (**D**), **5** (**E**), **6** (**F**), **7** (**G**), and **8** (**H**) binding to the *Tte* preQ$_1$ riboswitch. Aggregation at high concentrations is colored in red.

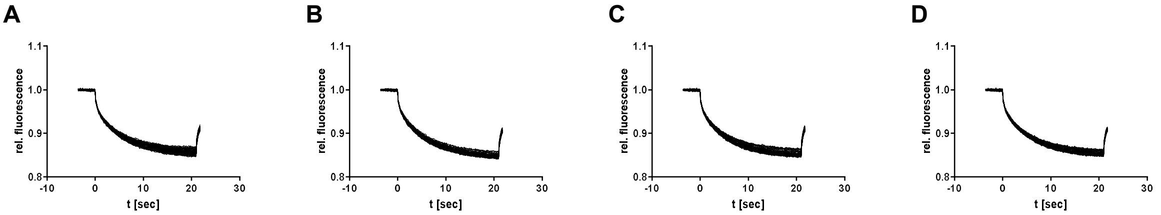

Figure S5. MST results at concentrations 0.1 nM, 0.316 nM, 1 nM, 3.16 nM, 10 nM, 31.6 nM, 100 nM, 316 nM, 1 µM, 3.16 µM, 10 µM, 31.6 µM, 100 µM, 316 µM, 1 mM for SAM (**A**), SAH (**B**), **1** (**C**), and **2** (**D**) binding to the *Ba* SAM-VI riboswitch.

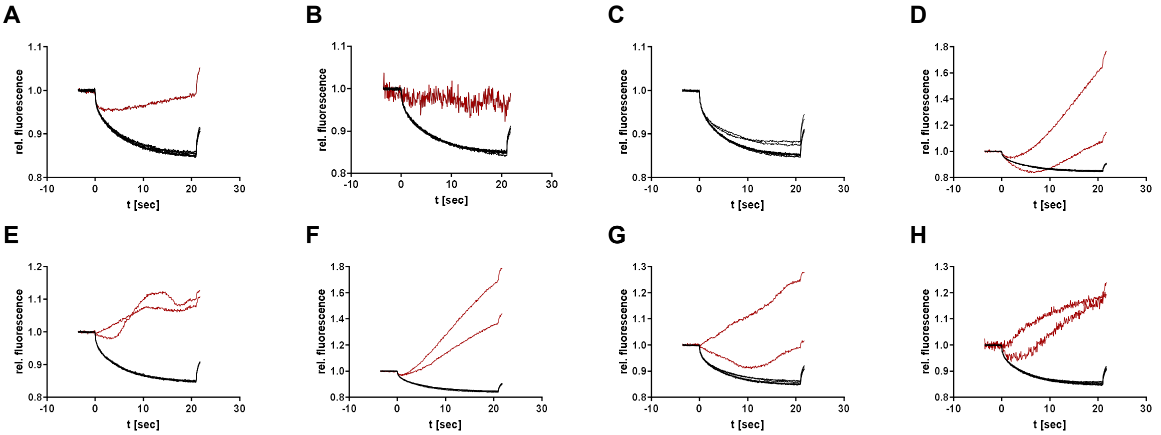

Figure S6. MST selectivity results at concentrations of 1 nM, 0.1 µM, 1 µM, 100 µM, 1 mM for preQ$_1$ (**A**), preQ$_0$ (**B**), **3** (**C**), **4** (**D**), **5** (**E**), **6** (**F**), **7** (**G**), and **8** (**H**) binding to the *Ba* SAM-VI riboswitch. For **4 (D)**, **6 (F)** and **8 (H)** concentrations of 1 nM, 0.1 µM, 100 µM, 316 µM, 1 mM were used. Aggregation at high concentrations is colored in red.

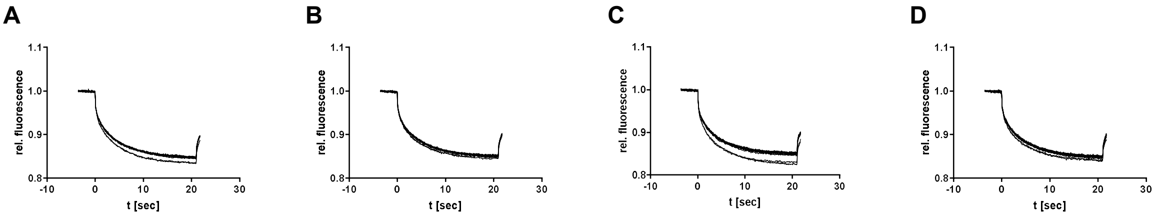

Figure S7. MST selectivity results at concentrations of 1 nM, 0.1 µM, 1 µM, 100 µM, 1 mM for SAM (**A**), SAH (**B**), **1** (**C**), and **2** (**D**) binding to the *Tte* preQ$_1$ riboswitch.

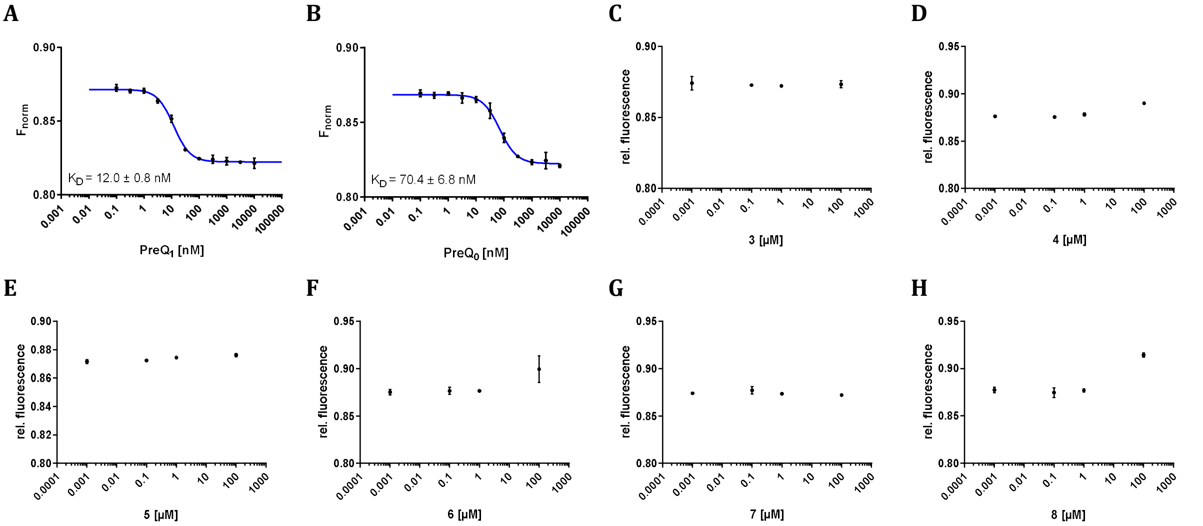

Figure S8. MST dose-response results for $preQ_1$ (**A**), $preQ_0$ (**B**), **3** (**C**), **4** (**D**), **5** (**E**), **6** (**F**), **7** (**G**), and **8** (**H**) binding to the *Tte* $preQ_1$ riboswitch (corresponding to MST traces Figure S4).

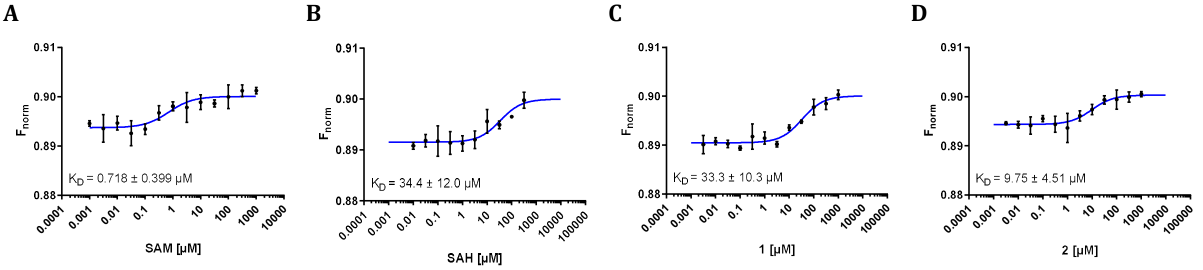

Figure S9. MST dose-response curves for SAM (**A**), SAH (**B**), **1** (**C**), and **2** (**D**) binding to the *Ba* SAM-VI riboswitch (corresponding to MST traces Figure S5).

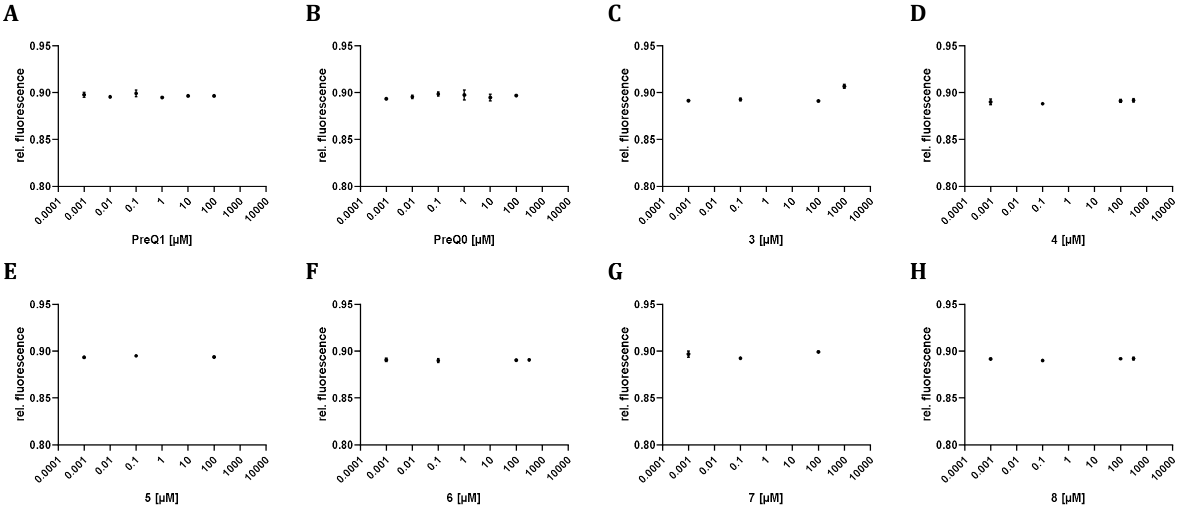

Figure S10. MST selectivity dose-response results for $preQ_1$ (**A**), $preQ_0$ (**B**), **3** (**C**), **4** (**D**), **5** (**E**), **6** (**F**), **7** (**G**), and **8** (**H**) not binding to the *Ba* SAM-VI riboswitch (corresponding to MST traces Figure S6).

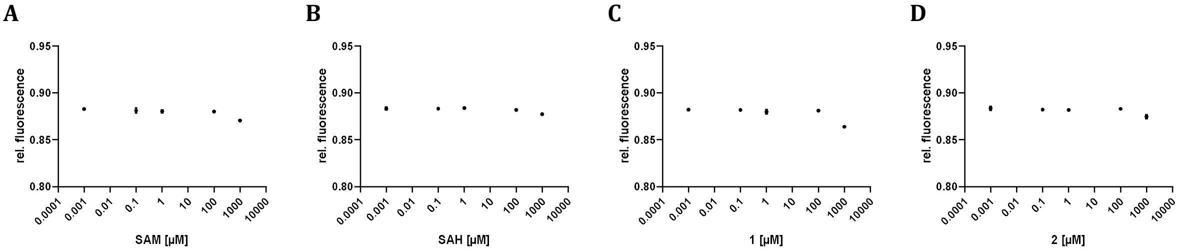

Figure S11. MST selectivity dose-response results for SAM (**A**), SAH (**B**), **1** (**C**), and **2** (**D**) not binding to the *Tte* $preQ_1$ riboswitch (corresponding to MST traces Figure S7).

3. ITC Experiments (Figures S12-S17, Tables S3-S6)

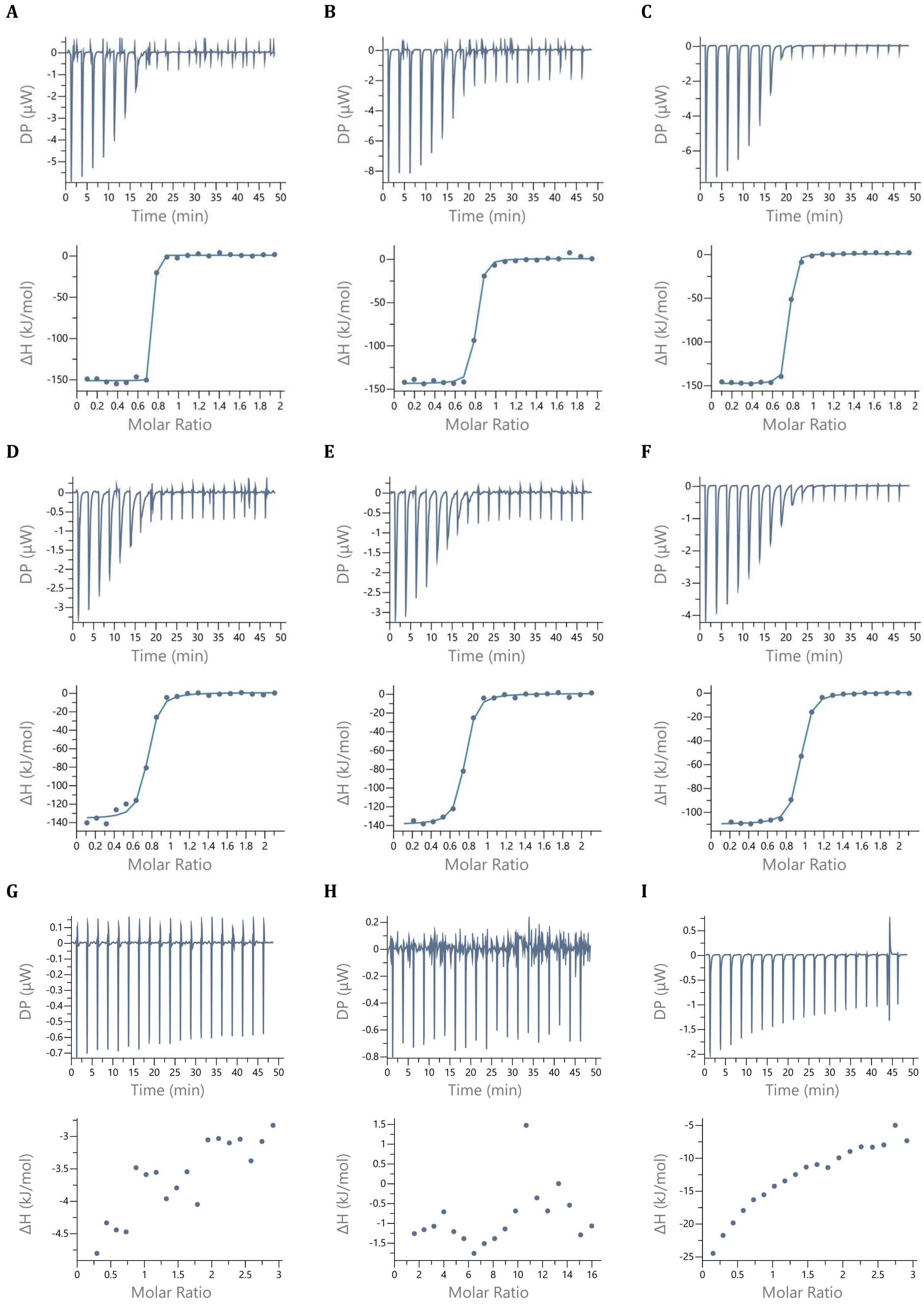

Figure S12. Thermograms (top and isotherms) from ITC experiments for preQ$_1$ (**A–C**), preQ$_0$ (**D–F**), **3** (**G**) with RNA: 30 μM and **3**: 300 μM, **4** (**H**) with RNA: 15 μM and **4**: 150 μM, **5** (**I**) with RNA: 30 μM and **5**: 300 μM, **6** (**J**) with RNA:30 μM and **6**: 300 μM, **7** (**K**) with RNA: 15 μM and **7**: 150 μM, and **8** (**L**) with RNA: 15 μM and **8**: 150 μM binding to the *Tte* preQ$_1$ riboswitch. For compounds **3**–**8**, no binding signals during titration were observed.

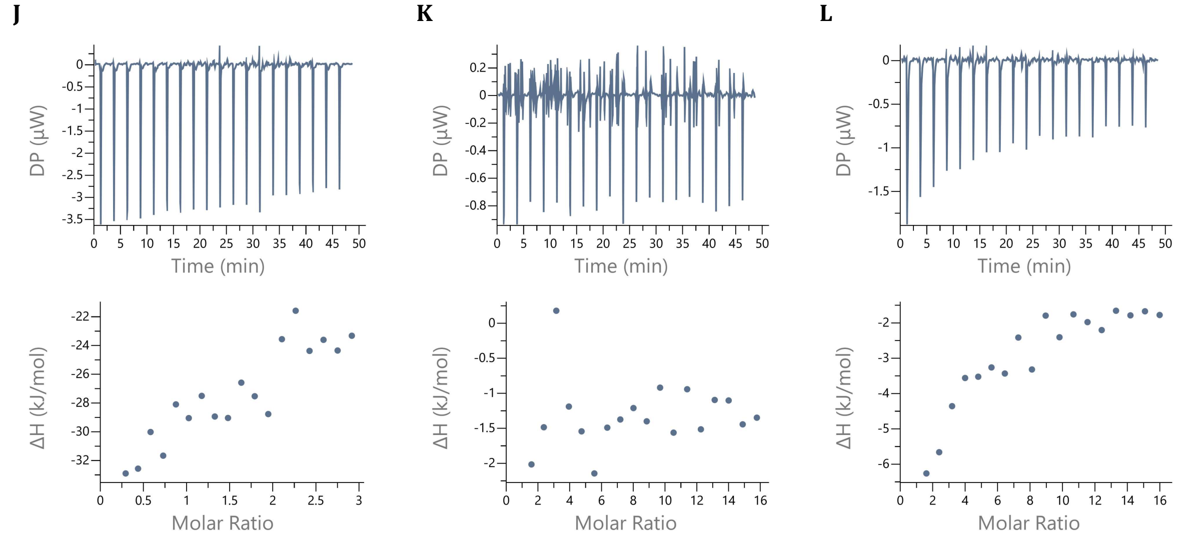

Figure S12 continued. Thermograms (top and isotherms) from ITC experiments for preQ$_1$ (**A–C**), preQ$_0$ (**D–F**), **3** (**G**) with RNA: 30 µM and **3**: 300 µM, **4** (**H**) with RNA: 15 µM and **4**: 150 µM, **5** (**I**) with RNA: 30 µM and **5**: 300 µM, **6** (**J**) with RNA:30 µM and **6**: 300 µM, **7** (**K**) with RNA: 15 µM and **7**: 150 µM, and **8** (**L**) with RNA: 15 µM and **8**: 150 µM binding to the *Tte* preQ$_1$ riboswitch. For compounds **3**–**8**, no binding signals during titration were observed.

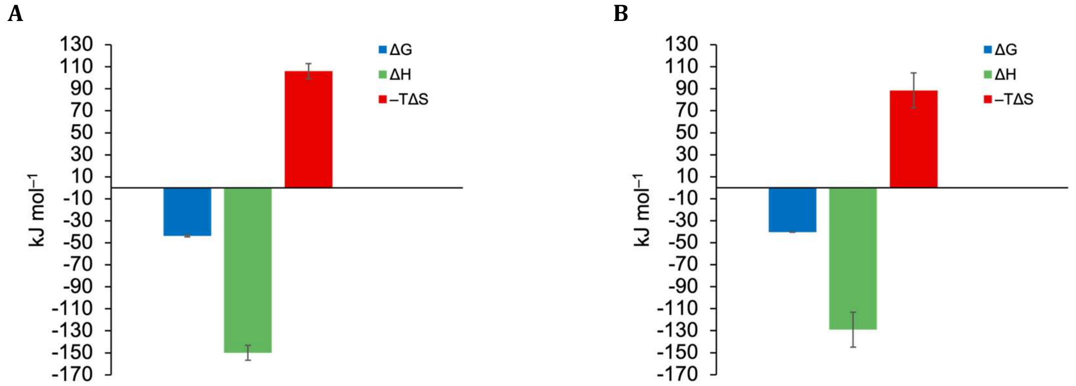

Figure S13. Thermodynamic signature plots of preQ$_1$ (**A**) and preQ$_0$ (**B**) binding to the *Tte* preQ$_1$ riboswitch.

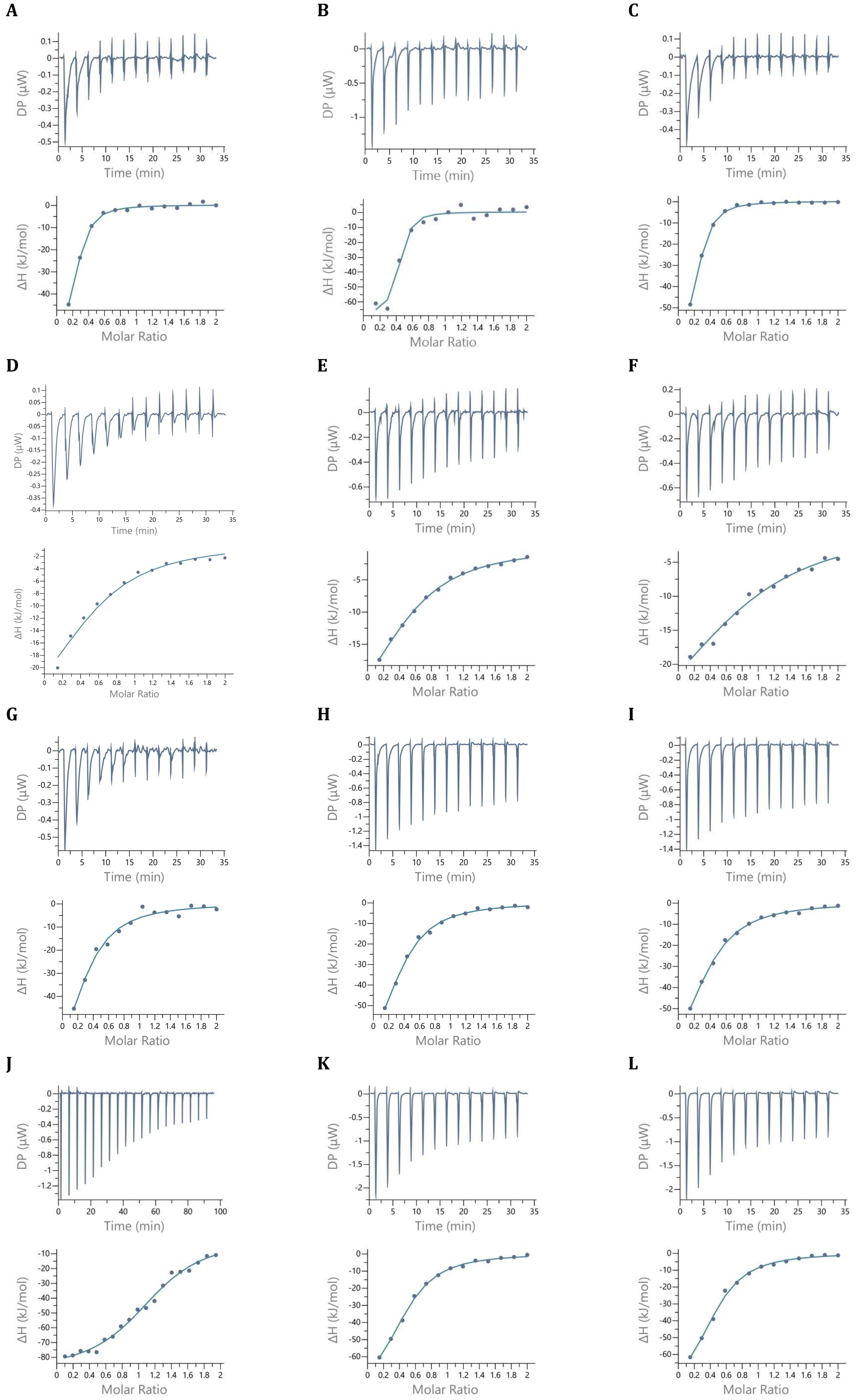

Figure S14. Thermograms (top) and isotherms (bottom) from ITC experiments for SAM (**A–C**), SAH (**D–F**), **1** (**G–I**), and **2** (**J–L**) binding to the *Ba* SAM-VI riboswitch.

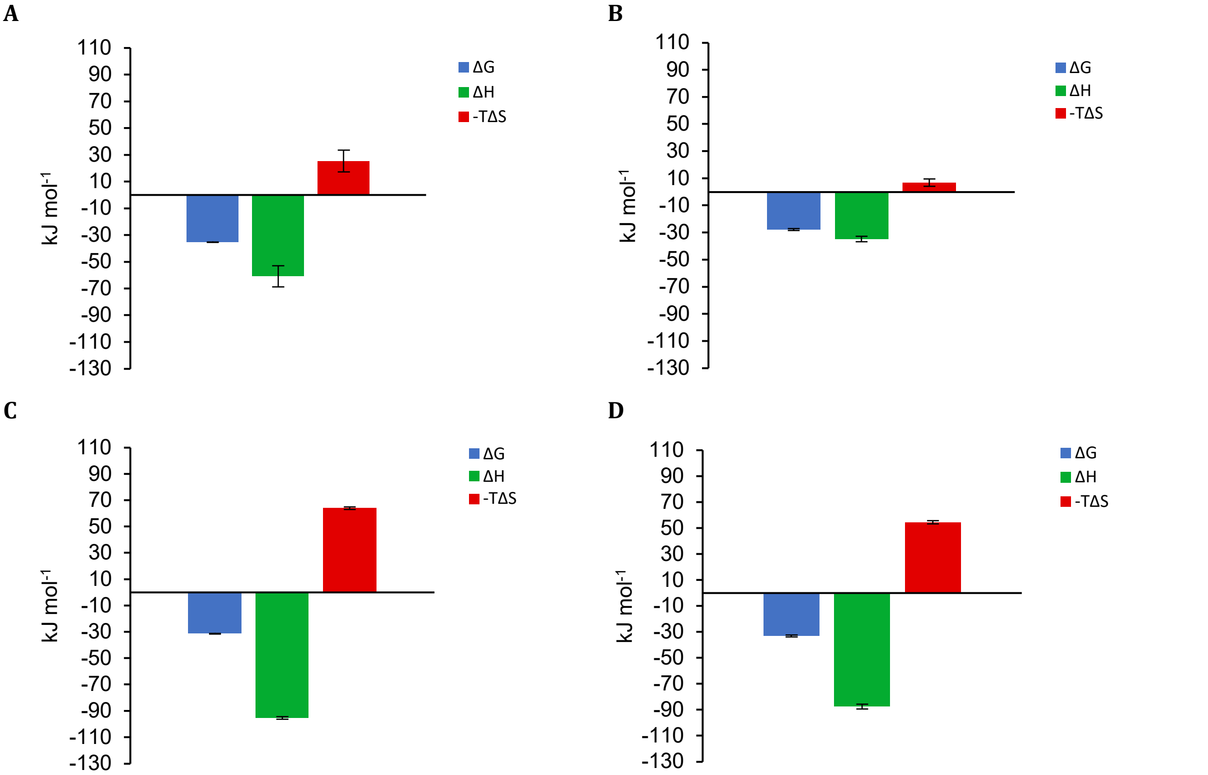

Figure S15. Thermodynamic signature plots of SAM (**A**), SAH (**B**), **1** (**C**), and **2** (**D**) binding to the *Ba* SAM-VI riboswitch.

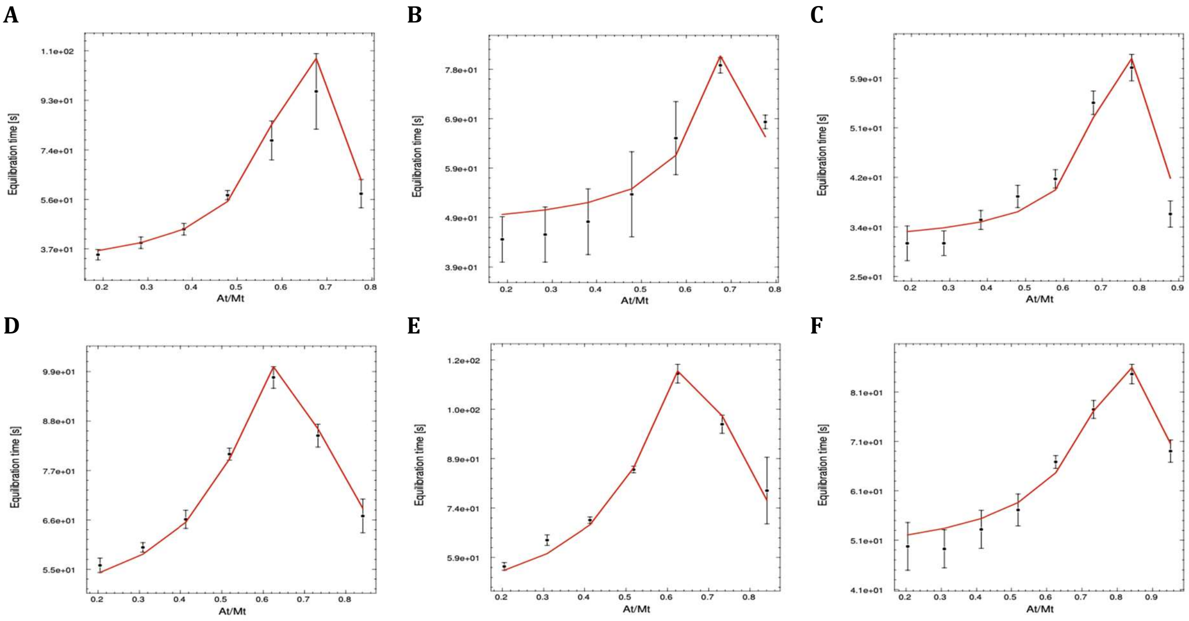

Figure S16. Equilibration-time curves (ETC) from kinITC analysis for preQ$_1$ (**A–B:** injections 2–8, **C**: injections 2–9) and preQ$_0$ (**D–E**: injections 2-8, **F**: injections 2–9) binding to the *Tte* preQ$_1$ riboswitch.

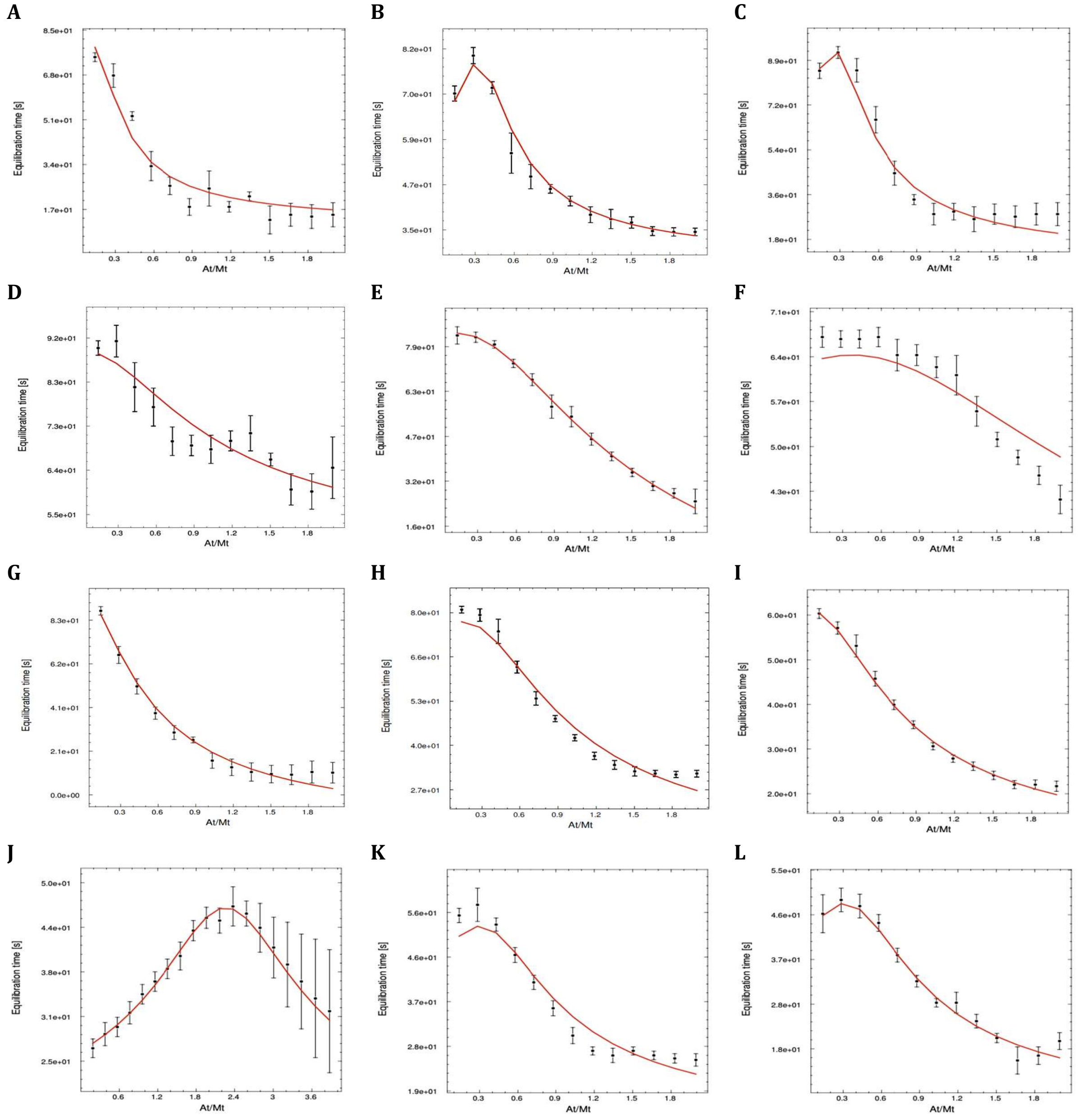

Figure S17. ETCs from kinITC analysis for SAM (**A**–**C**), SAH (**D**–**F**), **1** (**G**–**I**), and **2** (**J**–**L**) binding to the *Ba* SAM-VI riboswitch.

Table S3. Thermodynamic analysis results from ITC measurements for SAM, SAH, **1**, and **2** against the *Ba* SAM-VI riboswitch.

**Ligand**	**n**	**K_D [µM]**	**ΔG [kJ/mol]**	**ΔH [kJ/mol]**	**−TΔS [kJ/mol]**
**SAM**	0.20	0.688	−35.2	−64.8	29.6
	0.35	0.606	-35.5	-51.6	16.1
	0.30	0.720	−35.1	−65.8	30.7
**SAM Average**	**0.28**	**0.671 ± 0.059**	**−35.3 ± 0.2**	**−60.7 ± 7.9**	**25.5 ± 8.1**
**SAH**	0.54	10.9	−28.4	−33.2	4.86
	0.56	12.4	−28.0	−33.7	5.72
	0.86	18.8	−27.0	−36.9	9.90
**SAH Average**	**0.65**	**14.0 ± 4.2**	**−27.8 ± 0.7**	**−34.6 ± 2.0**	**6.8 ± 2.7**
**1**	0.28	3.43	-31.2	-96.2	65.0
	0.32	3.06	-31.5	-95.3	63.8
	0.34	3.44	-31.2	−94.3	63.1
**1 Average**	**0.31**	**3.31 ± 0.21**	**−31.3 ± 0.2**	**−95.3 ± 1.0**	**64.0 ± 1.0**
**2**	0.37	1.14	−34.0	−89.1	55.2
	0.45	2.07	−32.5	−85.6	53.1
	0.43	1.97	−32.6	−87.7	55.1
**2 Average**	**0.42**	**1.73 ± 0.51**	**−33.0 ± 0.8**	**−87.5 ± 1.8**	**54.5 ± 1.2**

Table S4. Kinetic analysis results from ITC measurements for SAM, SAH, **1**, and **2** against the *Ba* SAM-VI riboswitch.

**Ligand**	**k_{on} [1/Ms]**	**k_{off} [1/s]**	**chi^2**
**SAM**	$(2.50 \pm 0.08) \cdot 10^4$	$(1.72 \pm 0.06) \cdot 10^{-2}$	3.60
	$(2.41 \pm 0.04) \cdot 10^4$	$(1.46 \pm 0.03) \cdot 10^{-2}$	0.58
	$(1.49 \pm 0.05) \cdot 10^4$	$(1.07 \pm 0.04) \cdot 10^{-2}$	1.57
**SAM Average**	$\mathbf{(2.13 \pm 0.55) \cdot 10^4}$	$\mathbf{(1.42 \pm 0.33) \cdot 10^{-2}}$	
**SAH**	$(4.62 \pm 0.03) \cdot 10^3$	$(5.04 \pm 0.04) \cdot 10^{-2}$	1.48
	$(4.32 \pm 0.03) \cdot 10^3$	$(5.36 \pm 0.04) \cdot 10^{-2}$	0.18
	$(1.77 \pm 0.02) \cdot 10^3$	$(3.33 \pm 0.03) \cdot 10^{-2}$	4.98
**SAH Average**	$\mathbf{(3.57 \pm 1.57) \cdot 10^3}$	$\mathbf{(4.58 \pm 1.09) \cdot 10^{-2}}$	
**1**	$(2.38 \pm 0.03) \cdot 10^4$	$(6.74 \pm 0.08) \cdot 10^{-2}$	0.70
	$(1.20 \pm 0.04) \cdot 10^4$	$(3.66 \pm 0.13) \cdot 10^{-2}$	0.94
	$(1.04 \pm 0.03) \cdot 10^4$	$(3.57 \pm 0.09) \cdot 10^{-2}$	0.60
**1 Average**	$\mathbf{(1.54 \pm 0.73) \cdot 10^4}$	$\mathbf{(4.66 \pm 1.80) \cdot 10^{-2}}$	
**2**	$(1.25 \pm 0.08) \cdot 10^4$	$(1.42 \pm 0.09) \cdot 10^{-2}$	0.13
	$(1.42 \pm 0.05) \cdot 10^4$	$(2.95 \pm 0.10) \cdot 10^{-2}$	4.44
	$(1.41 \pm 0.07) \cdot 10^4$	$(2.78 \pm 0.13) \cdot 10^{-2}$	0.74
**2 Average**	$\mathbf{(1.36 \pm 0.10) \cdot 10^4}$	$\mathbf{(2.38 \pm 0.84) \cdot 10^{-2}}$	

Table S5. Thermodynamic analysis results from ITC measurements for $preQ_1$ and $preQ_0$ against the *Tte* $preQ_1$ riboswitch.

Ligand	n	K_D [nM]	ΔG [kJ/mol]	ΔH [kJ/mol]	−TΔS [kJ/mol]
$PreQ_1$	0.68	26.0	−43.3	−157	114
	0.76	28.7	−43.1	−144	101
	0.71	14.7	−44.8	−148	104
**$PreQ_1$ Average**	**0.72**	**23.1 ± 7.4**	**−43.7 ± 0.9**	**−150 ± 7**	**106 ± 7**
$PreQ_0$	0.71	88.5	−40.3	−137	96.2
	0.70	81.9	−40.5	−140	99.1
	0.90	85.9	−40.4	−111	70.2
**$PreQ_0$ Average**	**0.71**	**85.4 ± 3.3**	**−40.4 ± 0.1**	**−129 ± 16**	**88.5 ± 15.9**

Table S6. Kinetic analysis results from ITC measurements for $preQ_1$ and $preQ_0$ against the *Tte* $preQ_1$ riboswitch.

Ligand	k_{on} [1/Ms]	k_{off} [1/s]	chi^2
$PreQ_1$	$(4.10 \pm 0.37) \cdot 10^4$	$(1.07 \pm 0.10) \cdot 10^{-3}$	0.61
	$(8.33 \pm 0.71) \cdot 10^4$	$(2.39 \pm 0.20) \cdot 10^{-3}$	1.37
	$(9.59 \pm 0.75) \cdot 10^4$	$(1.41 \pm 0.11) \cdot 10^{-3}$	1.83
**$PreQ_1$ Average**	**$(7.34 \pm 2.88) \cdot 10^4$**	**$(1.62 \pm 0.69) \cdot 10^{-3}$**	
$PreQ_0$	$(3.41 \pm 0.13) \cdot 10^4$	$(3.01 \pm 0.12) \cdot 10^{-3}$	1.12
	$(2.64 \pm 0.08) \cdot 10^4$	$(2.16 \pm 0.06) \cdot 10^{-3}$	2.52
	$(3.80 \pm 0.30) \cdot 10^4$	$(3.26 \pm 0.26) \cdot 10^{-3}$	0.71
**$PreQ_0$ Average**	**$(3.28 \pm 0.59) \cdot 10^4$**	**$(2.81 \pm 0.58) \cdot 10^{-3}$**	

4. Analytical data compounds 3–8 (Figures S18-S23)

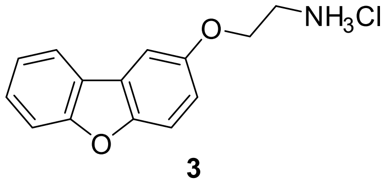

3

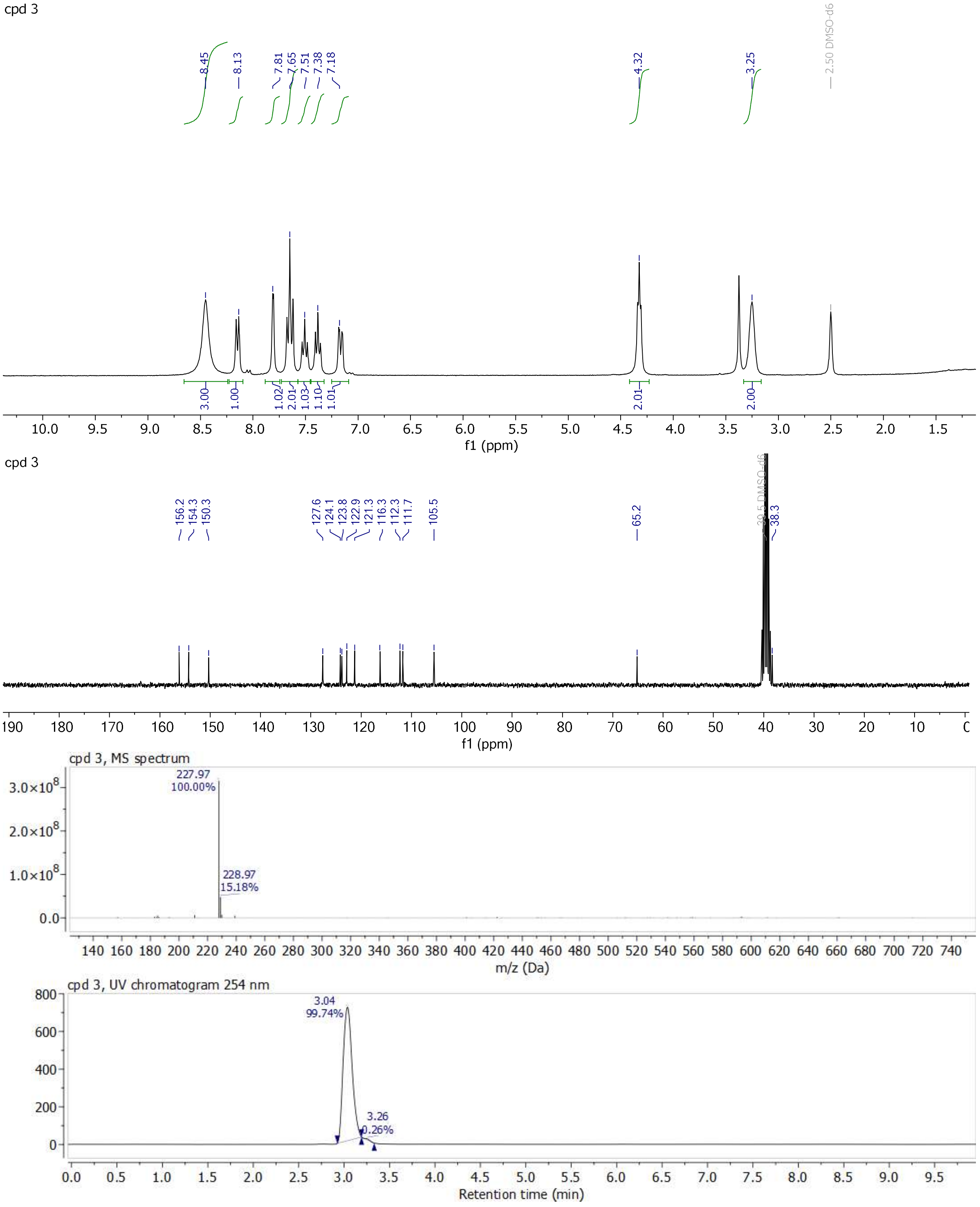

Figure S18. From top: ^{1}H-, ^{13}C-NMR spectra, ESI-MS spectrum and LC UV chromatogram at 254 nm of compound **3**. LC-ESI-MS measured with Zorbax column, isocratic 40% acetonitrile in water + 0.1% formic acid over 10 min.

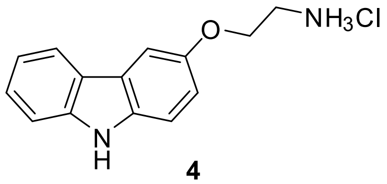

4

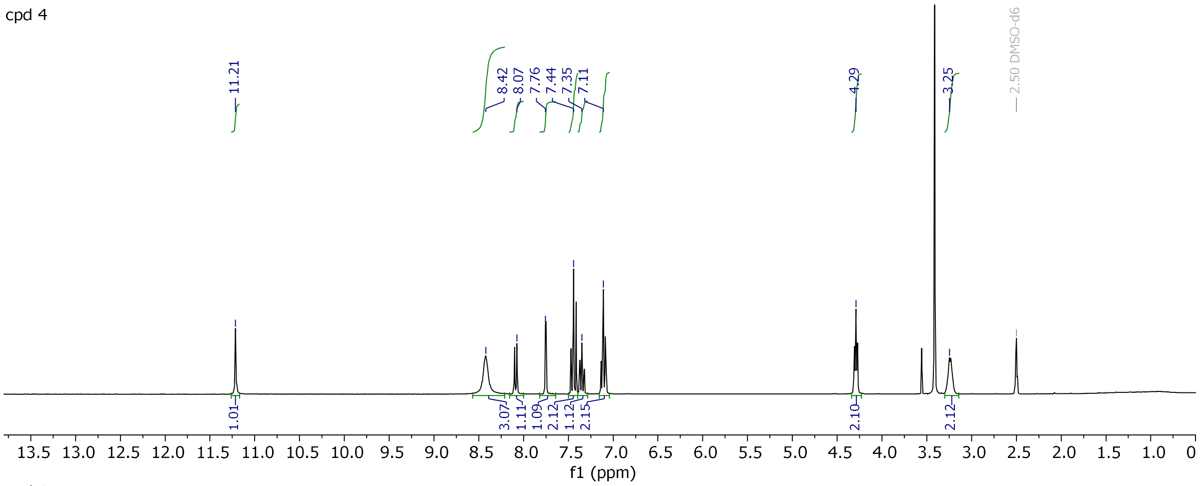

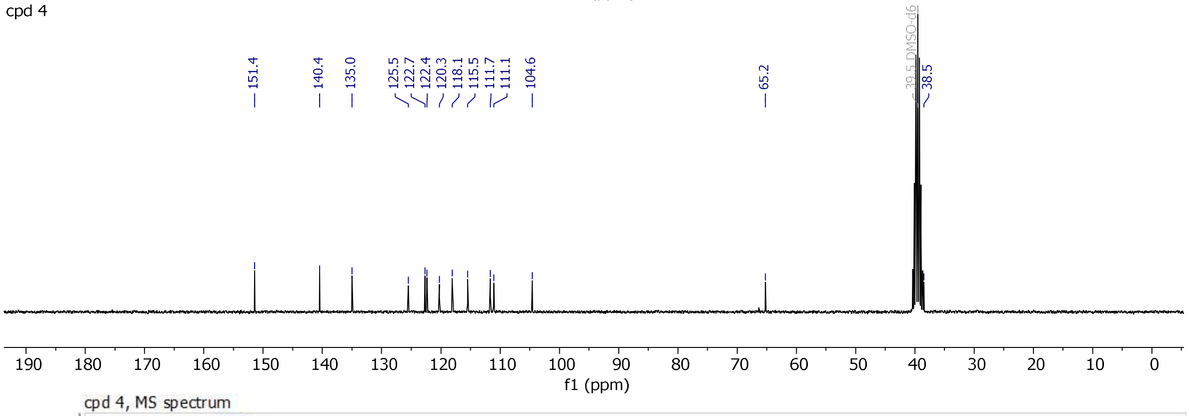

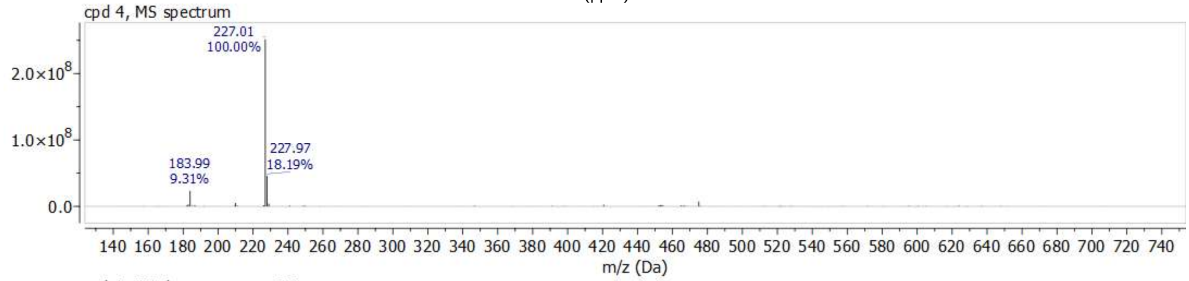

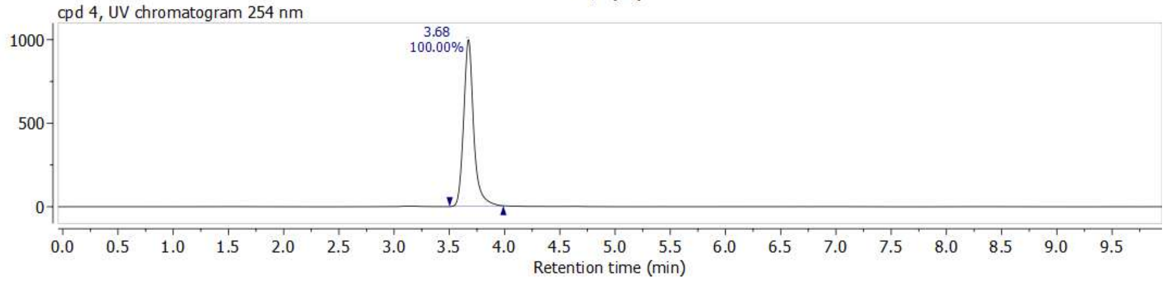

Figure S19. From top: ^{1}H-, ^{13}C-NMR spectra, ESI-MS spectrum and LC UV chromatogram at 254 nm of compound **4**. LC-ESI-MS measured with Zorbax column, isocratic 30% acetonitrile in water + 0.1% formic acid over 10 min.

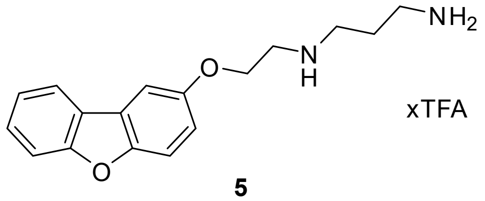

5

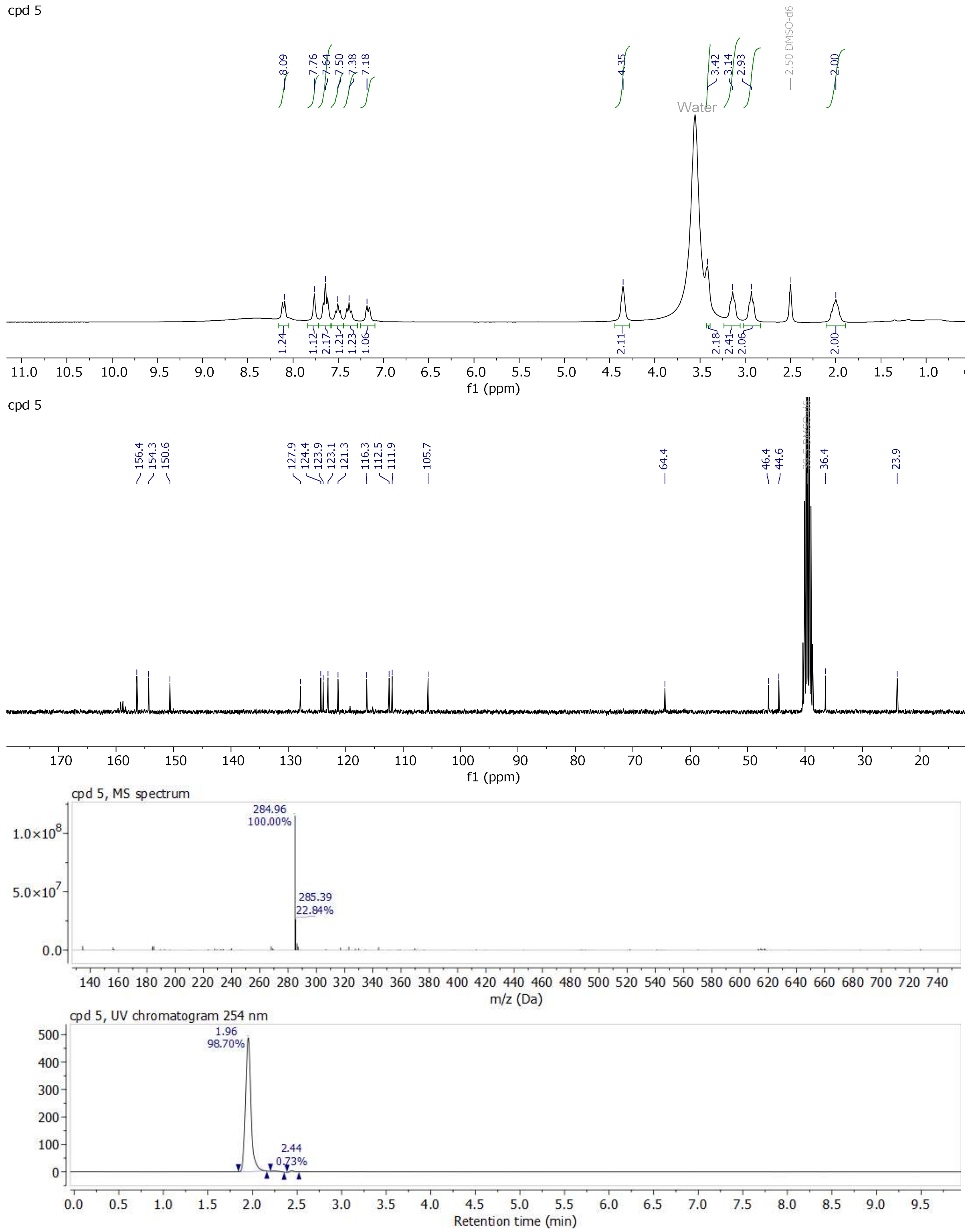

Figure S20. From top: ^{1}H-, ^{13}C-NMR spectra, ESI-MS spectrum and LC UV chromatogram at 254 nm of compound **5**. LC-ESI-MS measured with Zorbax column, isocratic 40% acetonitrile in water + 0.1% formic acid over 10 min.

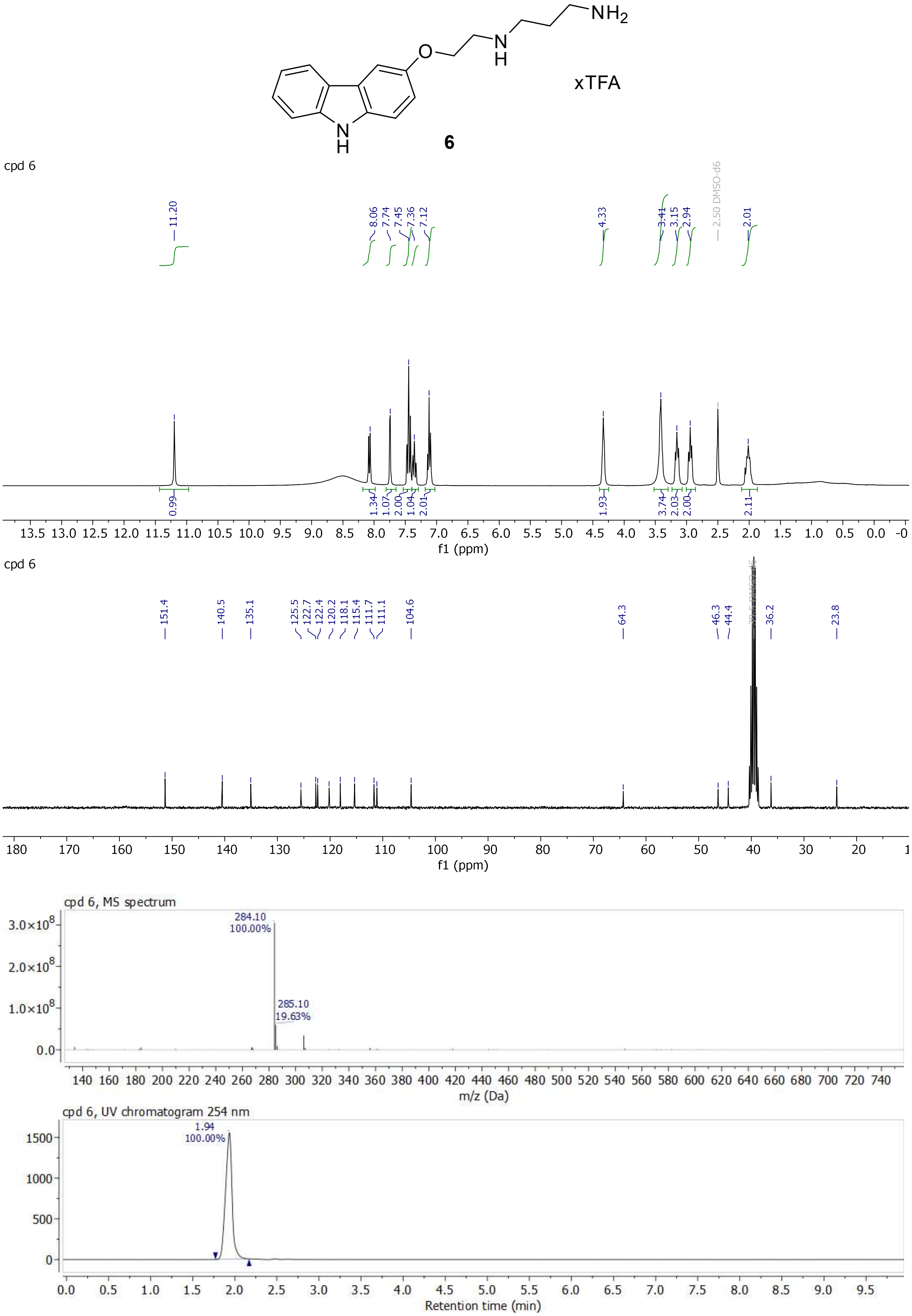

Figure S21. From top: ^{1}H-, ^{13}C-NMR spectra, ESI-MS spectrum and LC UV chromatogram at 254 nm of compound **6**. LC-ESI-MS measured with Zorbax column, isocratic 40% acetonitrile in water + 0.1% formic acid over 10 min.

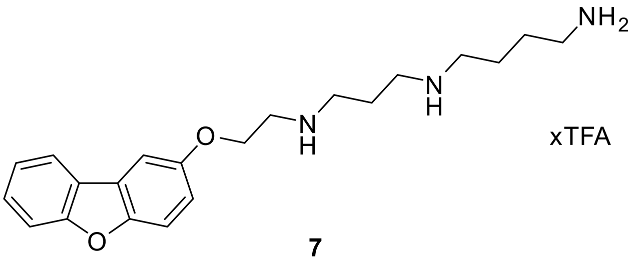

7

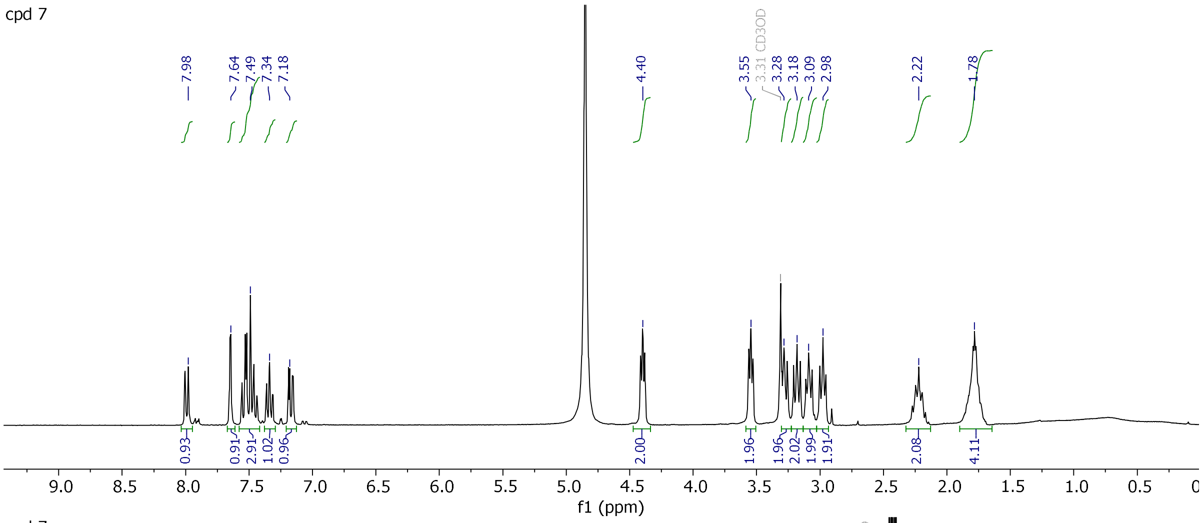

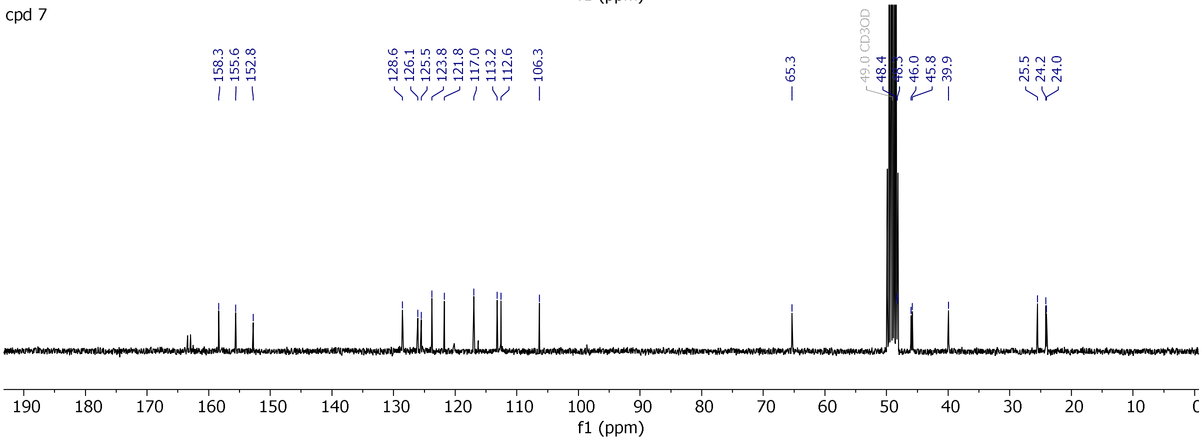

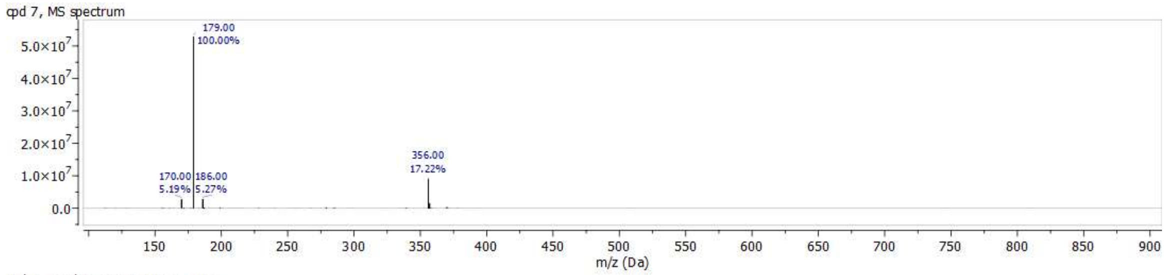

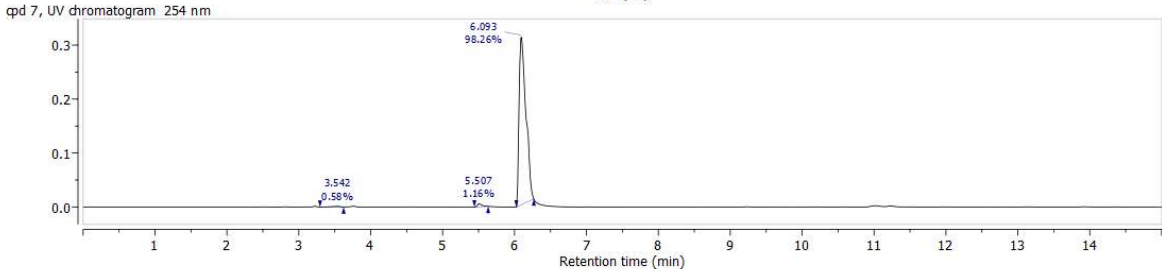

Figure S22. From top: ^{1}H-, ^{13}C-NMR spectra, ESI-MS spectrum and LC UV chromatogram at 254 nm of compound **7**. LC-ESI-MS measured with MZ-Aqua column, gradient 10–90% acetonitrile in water + 0.1% formic acid over 15 min.

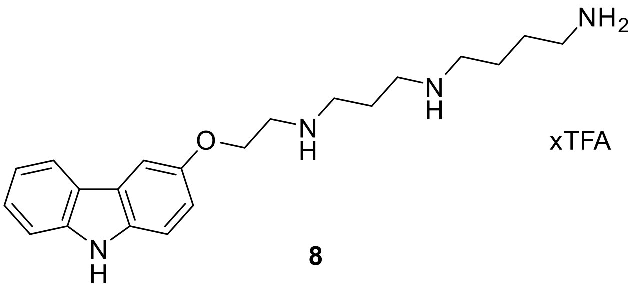

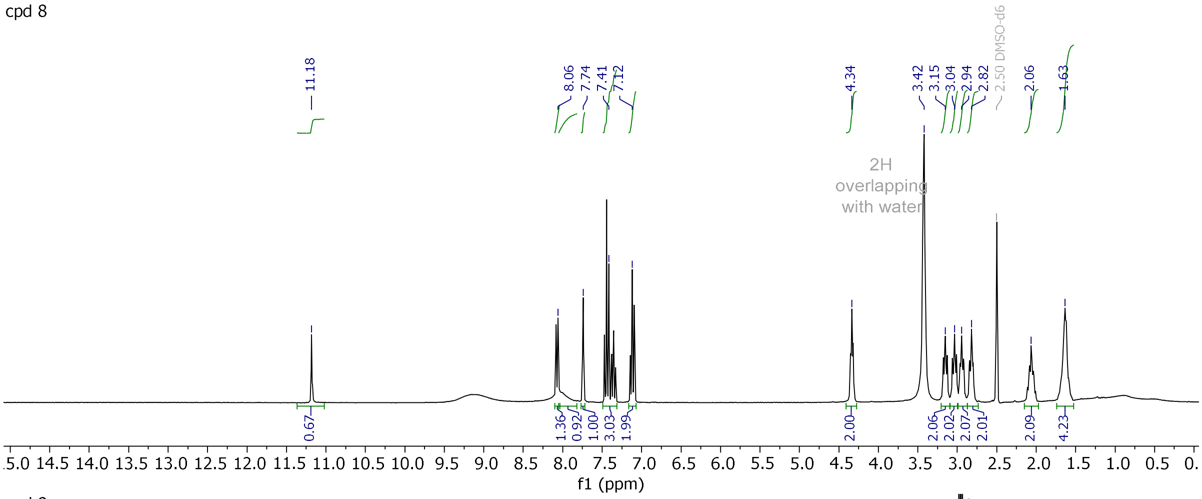

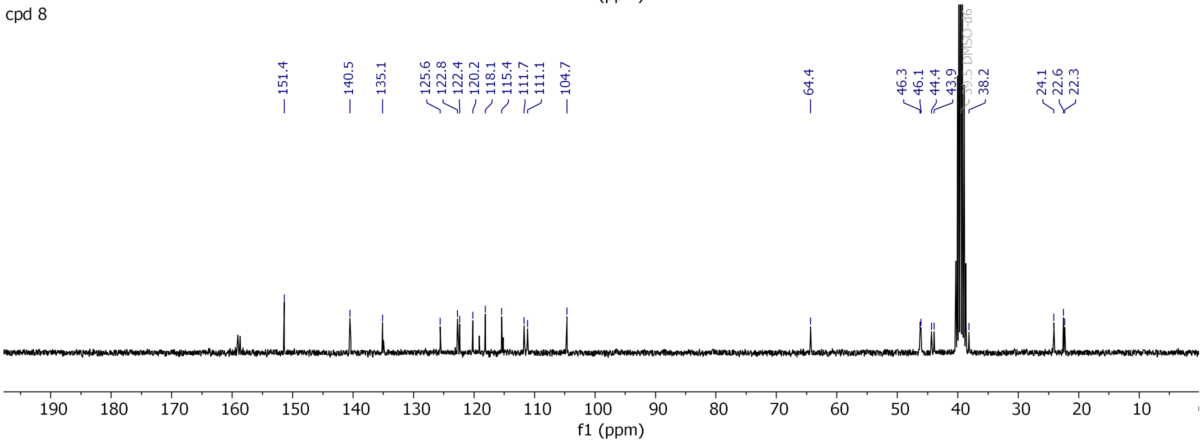

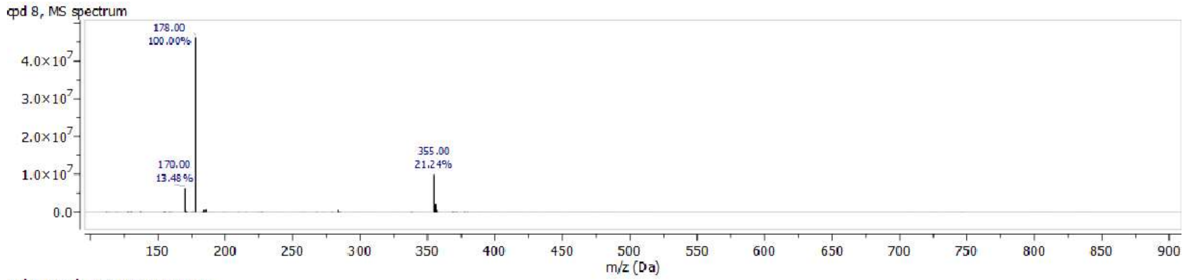

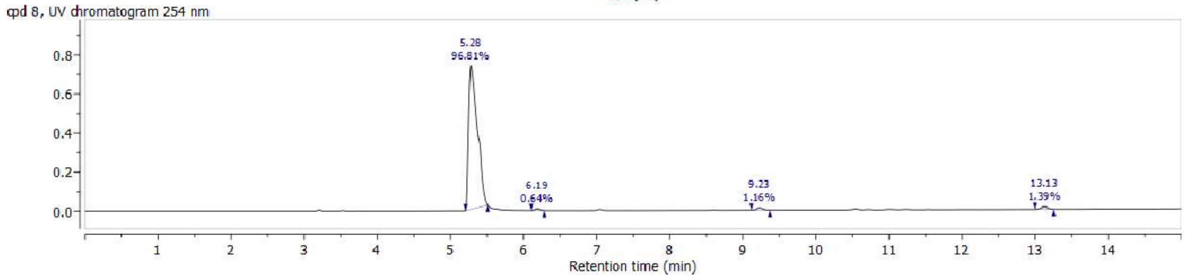

Figure S23. From top: ^{1}H-, ^{13}C-NMR spectra, ESI-MS spectrum and LC UV chromatogram at 254 nm of compound **8**. LC-ESI-MS measured with MZ-Aqua column, gradient 10–90% acetonitrile in water + 0.1% formic acid over 15 min.

5. Miscellaneous (Figures S24–S26, Tables S7, S8)

Table S7. Sequences of the 5’-labelled riboswitches.

Labelled RNA	Sequence
5’-Biotin-*Tte* preQ$_1$ riboswitch	Biotin-CUGGGUCGCAGUAACCCCAGUUAACAAAACAAG
5’-Cy5-*Tte* preQ$_1$ riboswitch	Cy5-CUGGGUCGCAGUAACCCCAGUUAACAAAACAAG
5’-Biotin-*Ba* SAM-VI riboswitch	Biotin-GGCAUUGUGCCUCGCAUUGCACUCCGCGGGGCGAUAAGUCCUGAAAAGGGAUGUC
5’-Cy5-*Ba* SAM-VI riboswitch	Cy5-GGCAUUGUGCCUCGCAUUGCACUCCGCGGGGCGAUAAGUCCUGAAAAGGGAUGUC

Table S8. Sequences of the DNA oligonucleotides used for the synthesis of the Ba SAM-VI riboswitch via PCR and IVT.

DNA oligonucleotide	Sequence
Ba SAM-VI riboswitch template	GACATCCCTTTTCAGGACTTATCGCCCCGCGGAGTGCAATGCGAGGCACAATGCCTATAGTGAGTCGTATTA
Ba SAM-VI riboswitch forward primer	CGCGCGAAGCTTAATACGACTCACTATA
Ba SAM-VI riboswitch reverse primer	GACATCCCTTTTCAGGACTTATCGCCCCG

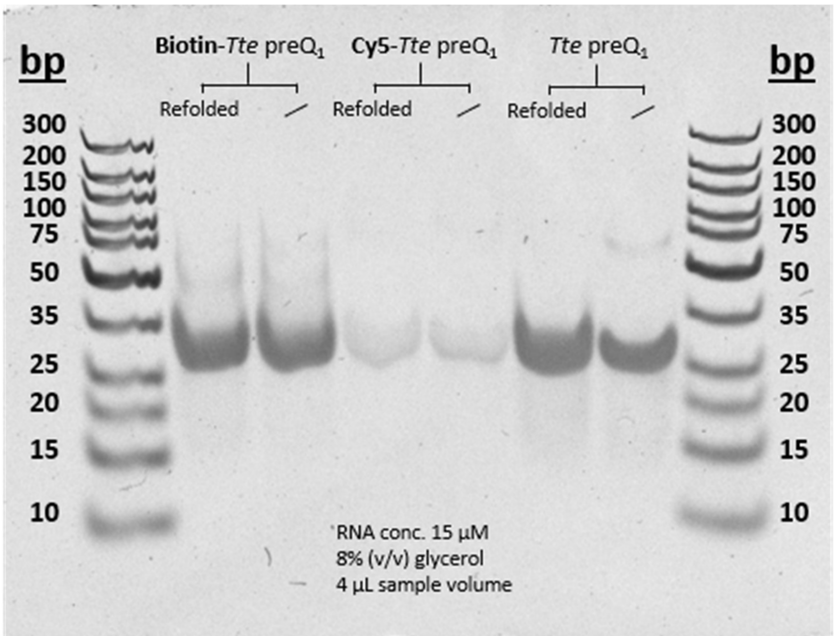

Figure S24: 10% native polyacrylamide gel electrophoresis (PAGE) of the used *Tte* preQ$_1$ riboswitch aptamer constructs (biotinylated for SPR, Cy5-labelled for MST and not labelled for ITC) to compare target heterogeny with and without refolding. Refolding was performed with a 20 µM RNA stock. The RNA stocks were heated to 75 °C for 5 min in buffer (80 mM Tris-HCl, pH 7.5, 200 mM KCl, 50 mM $MgCl_2$) and cooled down to room temperature for 20 min. GeneRuler™ Ultra Low Range DNA Ladder from Thermo Scientific™ was used in the first and last lane. Electrophoresis was performed at 120 V for 40 min all in 1x TBE-buffer. For nucleic acids band visualization GelRed™ was used and scanned at ethidium bromide channel on a Typhoon TRIO+ Variable Mode Imager GE Healthcare, UK.

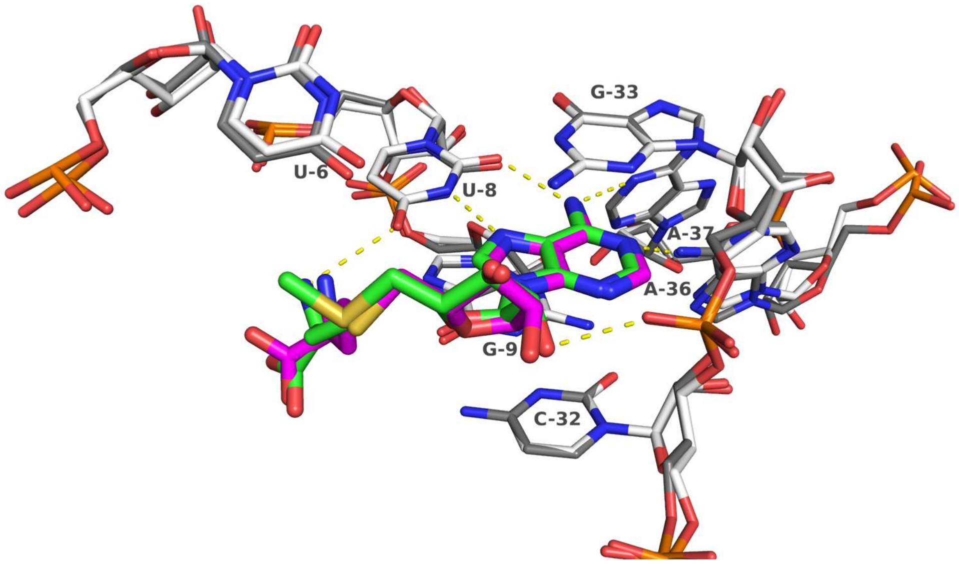

Figure S25. Superposition of the *Ba* SAM-VI riboswitch in complex with SAM (PDB-ID: 6LAS, green SAM carbon atoms, white RNA carbon atoms) and SAH (PDB-ID: 6LAU, magenta SAH carbon atoms, grey RNA carbon atoms).

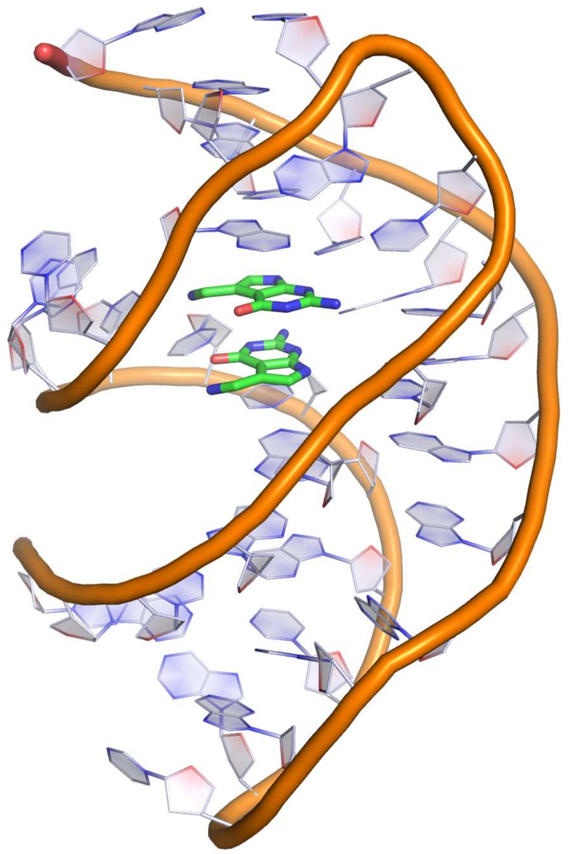

Figure S26. Crystal structure of *Carnobacterium antarcticum* $preQ_1$ type 1 riboswitch in complex with two $preQ_1$ molecules (green carbon atoms) (PDB-ID: 8FB3).
